# Genomic Determinants Encode the Reactivity and Regioselectivity of Flavin-Dependent Halogenases in Bacterial Genomes and Metagenomes

**DOI:** 10.1128/mSystems.00053-21

**Published:** 2021-05-27

**Authors:** Jehyun Jeon, Jaehee Lee, Se-Min Jung, Jae Hong Shin, Woon Ju Song, Mina Rho

**Affiliations:** a Department of Computer Science, Hanyang University, Seoul, Republic of Korea; b Department of Chemistry, Seoul National University, Seoul, Republic of Korea; c Department of Biomedical Informatics, Hanyang University, Seoul, Republic of Korea; Wageningen University

**Keywords:** halogenase, active sites, regioselectivity, profile hidden Markov model, genomic patterns, phylogeny, mutagenesis

## Abstract

Halogenases create diverse natural products by utilizing halide ions and are of great interest in the synthesis of potential pharmaceuticals and agrochemicals. An increasing number of halogenases discovered in microorganisms are annotated as flavin-dependent halogenases (FDHs), but their chemical reactivities are markedly different and the genomic contents associated with such functional distinction have not been revealed yet. Even though the reactivity and regioselectivity of FDHs are essential in the halogenation activity, these FDHs are annotated inaccurately in the protein sequence repositories without characterizing their functional activities. We carried out a comprehensive sequence analysis and biochemical characterization of FDHs. Using a probabilistic model that we built in this study, FDHs were discovered from 2,787 bacterial genomes and 17 sediment metagenomes. We analyzed the essential genomic determinants that are responsible for substrate binding and subsequent reactions: four flavin adenine dinucleotide-binding, one halide-binding, and four tryptophan-binding sites. Compared with previous studies, our study utilizes large-scale genomic information to propose a comprehensive set of sequence motifs that are related to the active sites and regioselectivity. We reveal that the genomic patterns and phylogenetic locations of the FDHs determine the enzymatic reactivities, which was experimentally validated in terms of the substrate scope and regioselectivity. A large portion of publicly available FDHs needs to be reevaluated to designate their correct functions. Our genomic models establish comprehensive links among genotypic information, reactivity, and regioselectivity of FDHs, thereby laying an important foundation for future discovery and classification of novel FDHs.

**IMPORTANCE** Halogenases are playing an important role as tailoring enzymes in biosynthetic pathways. Flavin-dependent tryptophan halogenases (Trp-FDHs) are among the enzymes that have broad substrate scope and high selectivity. From bacterial genomes and metagenomes, we found highly diverse halogenase sequences by using a well-trained profile hidden Markov model built from the experimentally validated halogenases. The characterization of genotype, steady-state activity, substrate scope, and regioselectivity has established comprehensive links between the information encoded in the genomic sequence and reactivity of FDHs reported here. By constructing models for accurate and detailed sequence markers, our work should guide future discovery and classification of novel FDHs.

## INTRODUCTION

Halogen-containing molecules are ubiquitous in the environment. An increasing number of organohalogen products have been isolated and characterized in recent decades (1). Organic halides produced as secondary metabolites are considered potential pharmaceuticals and agrochemicals, constituting an important class of antibiotics and antitumor drugs represented by rebeccamycin, chloramphenicol, and vancomycin derivatives (2). Because dehalogenation of these molecules often lowers binding affinity to the target (3) and biological activities (4), halogenation is one of the essential reactions in the functionalization of natural products. Halogenated molecules can also engage in specific noncovalent interactions with heteroatom-containing functional groups or aromatic rings by halogen bonds (5). The highly directional and specific halogen bonds have been exploited to enhance ligand binding affinities of drugs and inhibitors, with minimal disruption of other important intermolecular interactions (6).

Organohalogens are often generated by halogenases that belong to biosynthetic gene clusters. Depending on their native cofactors, cosubstrates, and reaction mechanisms, halogenases are categorized into four to five classes (2, 7–10). Among these, flavin-dependent halogenases (FDHs) have been extensively investigated due to their broad substrate scope and high selectivity. In particular, regioselective formation of the carbon-halogen bond represents an important class of reactions that produce aromatic compounds for feedstock. Consequently, artificial FDHs have emerged as viable surrogates to toxic halogenating chemical reagents (11–16).

Recently, numerous FDHs have been discovered and characterized in several directions. Sequence analysis of various FDHs were focused primarily on four strictly conserved motifs of the FAD-binding domain (17–19). Diverse halogenating activities of FDHs were investigated experimentally instead, resulting in FDHs that react with tryptophan (Trp-FDHs) and other aromatic functional groups, such as tyrosinyl (20), tryptophanyl (21, 22), and pyrrolyl moieties (23, 24). Phenolic halogenases, such as Rdc2 (25, 26), Gsfl (26, 27), and PltM (28), on the other hand, are reactive toward phenols and anilines. In addition, extensive activity profiling has been further carried out, expanding the substrate repertoire of FDHs (19, 29). Recent studies suggested that Trp-FDH enzymes have a subtle yet discrete substrate scope, revealing that not all react with tryptophan. MibH from Microbispora corallina (30) reacts with a lanthipeptide bearing a tryptophan moiety but not directly with tryptophan. BrvH from *Brevundimonas* sp. strain BAL3 (31) is inactive toward tryptophan but halogenates indole. KrmI from Theonella swinhoei WA (22) reacts with tryptophan but not with indole.

In addition to the substrate scope, regioselectivity is another key element in the identification of Trp-FDH reactivities. Based on the site of halogenation on the indole ring, Trp-FDHs can be further categorized into 5-, 6-, and 7-tryptophan halogenases (5-, 6-, and 7-Trp-FDHs, respectively) (7, 32, 33). Previous studies demonstrated that the regioselectivity of carbon-halogen bond formation is determined by the relative positioning of the substrate with respect to the catalytically critical lysine residue at the active site in conjunction with a glutamate located between the flavin adenine dinucleotide (FAD)-binding and tryptophan-binding domains (34, 35). Additionally, site-directed mutagenesis and directed evolution studies revealed that tryptophan-binding sites are critical to determine the substrate scope and regioselectivity of selected enzymes (16, 32, 33, 36–39).

The reactivity and regioselectivity of FDH are essential in halogenation activity and determined by the active sites that recognize aromatic substrates. However, no definitive set of genomic markers was analyzed from the large-scale sequence analysis to distinguish their reactivities at atomic accuracy. This is presumably due to the high variability in the amino acid composition of the substrate-binding pocket (40), and FDH sequence motifs that underpin such functional diversity are yet to be elucidated (41).

Recent studies have shown that whole genomes as well as metagenomes can serve as an immense source of halogenase genes. Using PCR-based screening, Liao et al. found halogenase genes from *Streptomyces* and *Nocardiopsis* in Artic marine actinomycete isolates (42). From forest soil microbiome and 11 Botany Bay metagenomes, Weigold et al. (43) and Neubauer et al. (31) discovered putative FDHs. The increasing amount of metagenome data provides more opportunities to find novel halogenases with different substrate specificities.

Here, we report a comprehensive profiling of FDHs from bacterial genomes and metagenomes. A well-trained profile hidden Markov model (pHMM), built from experimentally validated halogenases, allowed us to find highly diverse halogenase genes from bacterial genomes and metagenomes. To discover remotely homologous genes, our sequence models include information for conserved class-specific cofactors and binding sites. Structural analysis on FDHs was integrated to identify key genomic determinants that are responsible for substrate binding and subsequent reactions. Using the isolated bacterial and metagenomic halogenases, we determined the steady-state activity, substrate scope, and regioselectivity of FDHs, thereby establishing comprehensive links between genotypic information and functional sites. Our work thus lays an important foundation for the future discovery and classification of novel FDHs by constructing accurate and detailed sequence models.

## RESULTS

### Genomic patterns of halogenases in bacterial genomes and metagenomes.

We collected the experimentally validated FDH sequences to build a pHMM and searched homologous genes from 2,787 bacterial genomes and 17 sediment microbiomes (see Table S1 in the supplemental material). In total, 103 and 68 halogenases were identified using the pHMM search from the bacterial genomes and metagenomes, respectively (see Fig. S1). The 103 halogenases from bacterial genomes were further characterized into two groups: 20 located in the biosynthetic gene clusters (BGCs) and 83 that were not. Additionally, 109 halogenases were obtained from the NCBI protein sequence database by keyword and pHMM search.

10.1128/mSystems.00053-21.1 FIG S1 Summary of sequence collection and homology search for halogenases from bacterial genomes and metagenomes. The number of sequences is represented in parenthesis. Thirteen known Trp-FDHs which used to construct the pHMM were excepted from the results of pHMM search. Download FIG S1, TIF file, 0.6 MB. Copyright © 2021 Jeon et al. 2021 Jeon et al. https://creativecommons.org/licenses/by/4.0/ This content is distributed under the terms of the Creative Commons Attribution 4.0 International license.

10.1128/mSystems.00053-21.6 TABLE S1 Information for the metagenomic samples and halogenases that were used in training and identified in this study. (A) List of 17 metagenome samples of sediment microbiome. (B) The 33 known halogenases previously identified. (C) The 109 putative halogenases identified from the NCBI protein database. (D) The 20 putative halogenases that were identified from bacterial complete genomes and inside BGCs. (E) The 83 putative halogenases that were identified from bacterial complete genomes and outside BGCs. (F) The 68 putative halogenases identified from 17 sediment microbiomes. Download Table S1, DOCX file, 0.1 MB. Copyright © 2021 Jeon et al. 2021 Jeon et al. https://creativecommons.org/licenses/by/4.0/ This content is distributed under the terms of the Creative Commons Attribution 4.0 International license.

**FIG 1:**
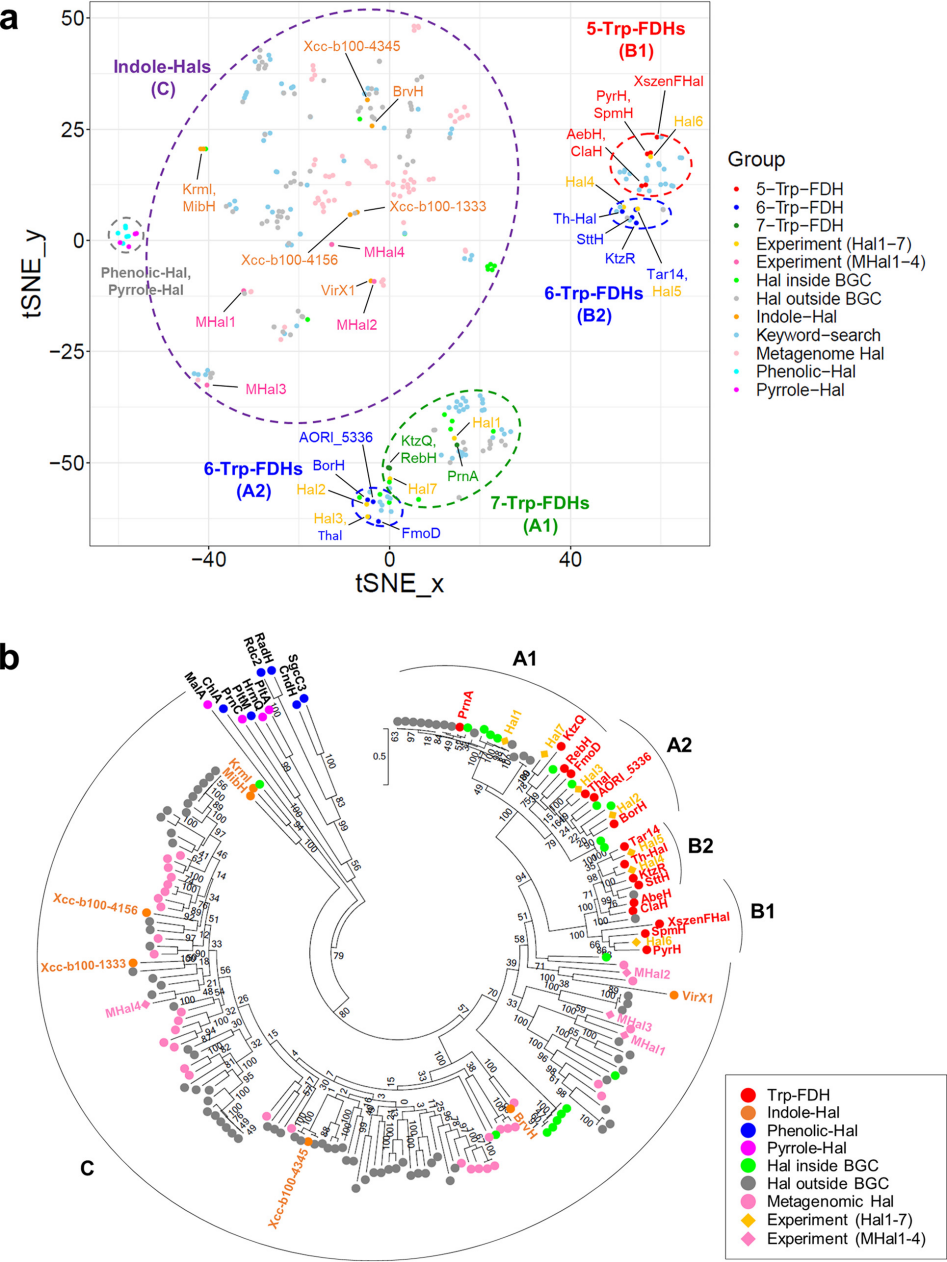
Known and putative halogenase sequences represented using t-SNE and a phylogenetic tree. (a) Embedding of the putative halogenases identified from the bacterial genomes and marine metagenomes, in conjunction with the known halogenases based on genomic patterns. (b) Phylogenetic tree of the halogenases.

To investigate the genomic patterns of halogenases, the 280 putative and 33 known halogenases were represented on embedding space (Fig. 1a). The amino acid conservation against the profiles of six different halogenase groups, 5-, 6-, and 7-Trp-FDHs, indole-Hals, phenolic-Hals, and pyrrole-Hals, was used to calculate the distance among halogenase genes using *t*-distributed stochastic neighbor embedding (t-SNE). Halogenases represented as the points were grouped according to their substrate types and scopes when the known halogenases were compared. Especially, Trp-FDHs were clustered into four groups, 7-Trp-FDH (A1), 6-Trp-FDH (A2), 6-Trp-FDH (B2), and 5-Trp-FDH (B1), implying that the regioselectivity is reflected in the embedding space. Interestingly, 6-Trp-FDHs in the B2 group, including KtzR (44), SttH (45), Tar14 (46), and thermophilic Hal (Th-Hal) (47), were relatively close to 5-Trp-FDHs, while 6-Trp-FDHs in the A2 group, including AORI_5336 (48), BorH (49), FmoD (50), and Thal (51), were close to 7-Trp-FDHs. Halogenases identified from marine sediments were clustered with most of the indole halogenases such as BrvH (31), KrmI (22), MibH (30), VirX1 (52), Xcc-b100-1333, Xcc-b100-4156 (53), and Xcc-b100-4345 (54). Phenolic and pyrrole halogenases were clustered, which are apart from Trp-FDHs.

Similar patterns were also observed in the phylogenetic tree that was constructed with the same set of Trp-FDH and non-Trp-FDH sequences (Fig. 1b). The FDHs were grouped into three discrete groups, A, B, and C, in the tree. Consistently, the 17 known Trp-FDHs were clustered in groups A and B, whereas non-Trp-FDHs, such as MibH, BrvH, and KrmI, were found in group C, showing that the substrate scope is associated with the branch grouping. Regioselectivity appeared to be represented phylogenetically: 7-Trp-FDHs and 5-Trp-FDHs were found in groups A and B, respectively. Interestingly, 6-Trp-FDHs were found in both groups A and B, which was also observed in the embedding space in Fig. 1a.

Group A was further divided into two subgroups, A1 and A2, in the phylogenetic tree (Fig. 1b), which correspond to two close groups in the embedding space (Fig. 1a, bottom). Here, 7-Trp-FDHs, including pyrrolnitrin halogenase (PrnA) from Pseudomonas fluorescens (55), were found in group A1, and 6-Trp-FDHs, including ThdH (Thal) from Streptomyces albogriseolus (51), ThnH from *Streptomyces* sp. FXJ1.172 (56), BorH (49), and AORI_5336 (48), were found in group A2. Even though FmoD (50) was closely clustered with other 6-Trp-FDHs in the embedding space, it showed an ambiguous placement with KtzQ (44) and rebeccamycin halogenase (RebH) (57) of 7-Trp-FDHs in group A in the phylogenetic tree.

Group B was also divided into two subgroups, B1 and B2, in the phylogenetic tree. All 5-Trp-FDH genes, such as those encoding ClaH (58), AbeH (59), SpmH (60), XszenFHal (61), and PyrH (18), were included in group B1, whereas 6-Trp-FDH genes, such as those encoding Th-Hal (47), KtzR (44), Tar14 (46), and SttH (45), were in group B2. Therefore, Trp-FDHs showed distinctive sequence conservation patterns that impart regioselectivity to tryptophan. Even though ClaH (58) and AbeH (59) ambiguously exist between branch B1 and B2 in the phylogenetic tree, they were clustered more closely with 5-Trp-FDH genes in the embedding space according to the classification method.

### Structure-guided analysis of FAD- and halide-binding regions in FDHs.

**FIG 2:**
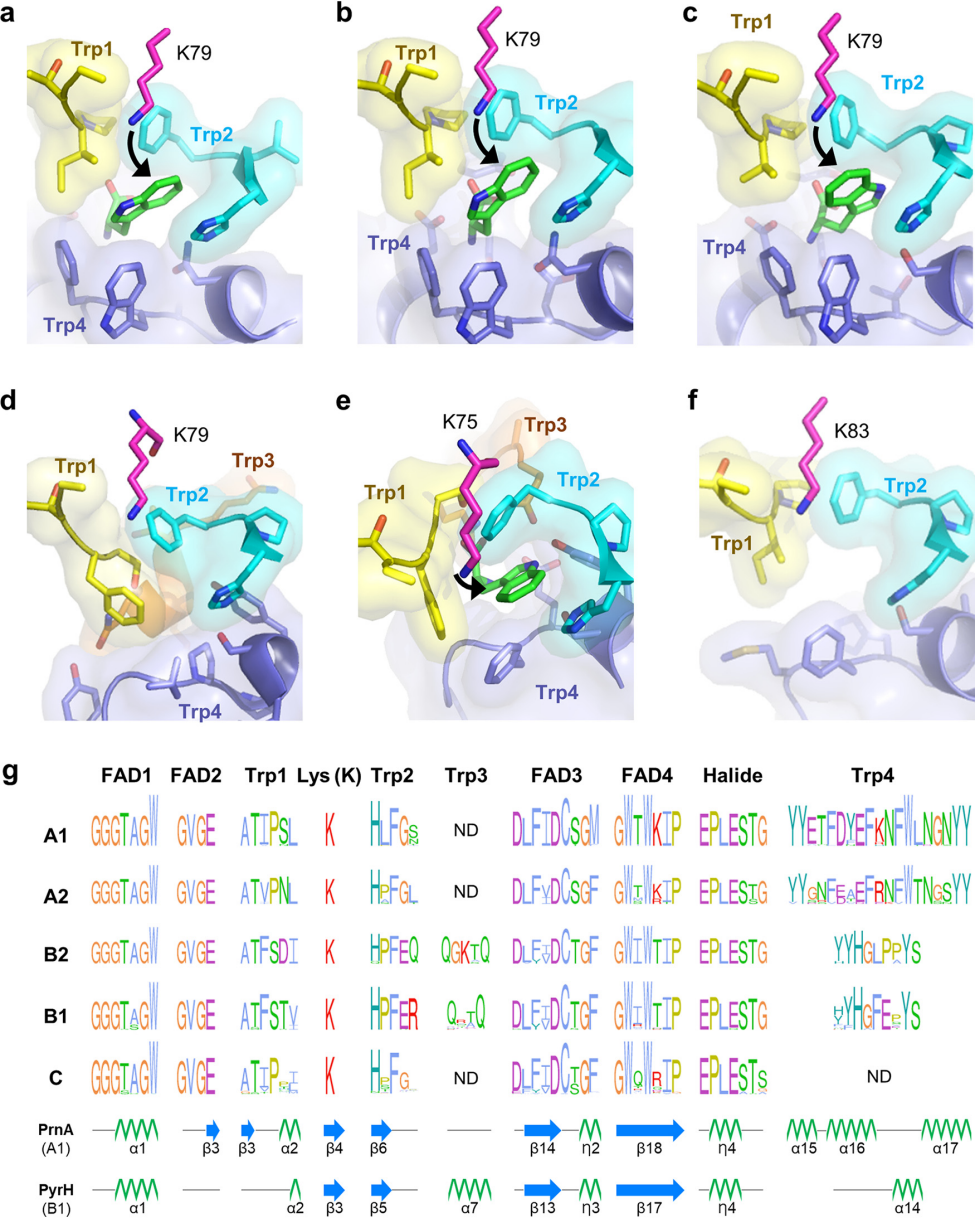
Integrated structure and sequence analysis of tryptophan and nontryptophan halogenases. Tryptophan-binding sites determined in this study are denoted for each group in Fig. 1. (a) PrnA in A1 (2AQJ); (b) RebH in A1 (2OA1); (c) Thal in A2 (6H44); (d) SttH in B2 (5HY5); (e) PyrH in B1 (2WET); (f) BrvH in C (6FRL). PDB codes are in parentheses. Residues comprising Trp1 to -4 are colored yellow, cyan, orange, and purple, respectively. Tryptophan molecules and catalytic lysine residues are shown in green and magenta bars, respectively. (g) Multiple-sequence alignment and conservation sites of FAD1 to -4, Lys (K), Trp1 to -4, and halide sites of FDHs. Secondary structure for each motif site is represented as α-helices, β-sheets, and loops using PrnA and PyrH.

To elucidate protein sequence-structure-activity relationships among FDHs, conserved regions were investigated. We screened 3-mers (i.e., sequences of three amino acids) using a multiple sequence alignment of 313 sequences (see Fig. S2A). Regions of low entropy were investigated using X-ray crystal structures of FDHs that are complexed with FAD, chloride, and tryptophan (Fig. 2a to f; Fig. S2B to H). We defined substrate-binding sites as the residues that make direct contacts with the molecule. As a result, 10 putative functional regions, FAD1 to -4, Lys (K), halide, and Trp1 to -4, were determined as well-conserved regions and subjected to further investigation (Fig. 2g).

10.1128/mSystems.00053-21.2 FIG S2 Functional sites of FDHs and indole halogenases. (A) Entropy of 3-mers through MSA for five groups (A1, A2, B1, B2, and C). The red lines in the graphs indicate functional sites of halogenases. Each functional site is represented by the following order: FAD1, FAD2, Trp1, Trp2, FAD3, FAD4, halide, and Trp4 in A1-A2; FAD1, FAD2, Trp1, Trp2, Trp3, FAD3, FAD4, halide, and Trp4 in B1-B2; FAD1, FAD2, Trp1, Trp2, FAD3, FAD4, and halide in C. (B) Flavin (FAD) and halide-binding sites in the overlaid structures of Trp-FDHs. PyrH (PDB 2WET), SttH (5HY5), and PrnA (2AQJ) are colored light magenta, light blue, and gray, respectively. FAD1 to -4 motifs are colored in red, orange, green, blue, and cyan, respectively. The protein-bound FAD and tryptophan are depicted with yellow sticks and magenta spheres. Chloride ion encapsulated by the halide-binding site is shown with a green sphere. (C to F) Structural analysis of enzyme-substrate interactions between the residues in the tryptophan binding sites and bound tryptophan substrates. (C) 7-Trp-FDH PrnA (PDB 2AQJ); (D) 7-Trp-FDH RebH (PDB 2OA1); (E) 6-Trp-FDH Thal (PDB 6H44); (F) 5-Trp-FDH PyrH (PDB 2WET). Tryptophan molecules and catalytic lysine residues are shown with green and magenta sticks, respectively. Hydrogen-bonding interactions and water molecules are shown with black dots and red spheres, respectively. The numbers in parentheses indicate the tryptophan binding motifs described for Fig. 2. (G to H) Crystal structures of MibH and BrvH. MibH (PDB 5UAO, colored green) (G) or BrvH (PDB 6FRL, colored purple) (H)was overlaid with PrnA (PDB 2AQJ, gray). The cofactor or substrate bound in PrnA is colored magenta. Trp4 motif derived from PrnA (colored darker gray) is absent in MibH and BrvH. Download FIG S2, TIF file, 1.1 MB. Copyright © 2021 Jeon et al. 2021 Jeon et al. https://creativecommons.org/licenses/by/4.0/ This content is distributed under the terms of the Creative Commons Attribution 4.0 International license.

We found four highly conserved regions related to FAD-binding (FAD1 to -4), as reported previously (18). We noted that FAD1 and FAD2 regions are consistently conserved in all identified FDHs in groups A to C. In contrast, FAD3 and -4 are less conserved than FAD1 and -2, exhibiting slight sequence alterations among groups. However, it is unclear whether the sequence variation in FAD3 and -4 is directly related to regioselectivity, as FAD3 and FAD4 are located remotely from the tryptophan-binding motifs.

The halide-binding site is also highly conserved in the sequences and structures of genomic and metagenomic FDHs. A large number of Trp-FDHs, including RebH (32, 57) and PyrH (18), react with both Cl^−^ and Br^−^; the yields are either comparable to or slightly higher with Cl^−^. In contrast, the recently identified halogenase BrvH exhibits catalytic reactivity only with Br^−^ (31), indicating that the halogenation activity is less likely determined by the halide-binding motif alone. Because the activated halogenating species, such as hypochlorite (ClO^−^) or hypobromite (BrO^−^), are likely to transfer from the halide-binding sites to tryptophan-binding regions, unidentified residues that are associated with the dynamic process might be related to halogen-specific reactivity.

### Identification of tryptophan-binding regions in Trp-FDHs.

In addition to the FAD- and halide-binding sites, the interactions with tryptophan are essential for halogenation (Fig. 2a to f). We determined Trp1 to -4 regions with low entropy of amino acid sequences (Fig. 2g): Trp1 (amino acids [aa] 50 to 55 in PrnA), Trp2 (aa 101 to 105 in PrnA), Trp3 (aa 160 to 163 in PyrH), and Trp4 (aa 443 to 461 in PrnA). Notably, different groups showed different patterns of tryptophan-binding regions. Trp1 and Trp2 motifs are found in all genes of groups A and B, whereas Trp3 is present only in group B, and Trp4 is present differentially in groups A and B. The Trp4 motif was not observed in group C.

For halogenases that have different regioselectivity for tryptophan, the Trp1 to -4 regions show considerable sequence variations. In particular, the third and fourth positions in Trp1 have conservation with Ile (I), Val (V), and Phe (F) at 63%, 15%, and 14%, respectively, followed by Pro (P) and Ser (S) with 84% and 13%, respectively. They exhibit considerably high conservation within each subgroup, indicating that the regioselectivity could be related to the Trp1 motif. Structural analysis showed that the Trp1 motif undergoes considerable structural changes upon tryptophan binding (62). In particular, the third through fifth residues are involved in hydrophobic and hydrogen-bonding interactions with tryptophan (Fig. 2a to f), suggesting that Trp1 determines the regioselectivity (see the experiment results in the next section).

The Trp2 region also exhibits high sequence conservation. Here, hydrophobic residues constitute the first and third positions with 95% and 98% conservation, respectively, and engage in π-π stacking interactions with the indole ring portion of tryptophan. Although the side chain of the second residue in the HxF motif is not directed toward the substrate, noticeable sequence conservation is observed depending on phylogeny: Leu (L) in branch A1, and Pro (P) in branches A2, B1, and B2 (Fig. 2g). These observations imply that the least-conserved second residue in Trp2 might relate to altered orientations of the hydrophobic residues for π-π stacking interactions with tryptophan. Indeed, hydrogen-bonding interactions are observed between the indole N-H and the carbonyl group of Pro in the Trp2 motif in PyrH, suggesting its potential roles in regioselectivity (see the experiment results in the next section).

The Trp3 region occurs only in branch B, whereas variations are observed in a number of nonconserved residues between two Gln (Q)s as QxxQ and QxxxQ in groups B1 and B2, respectively (Fig. 2g). The enzyme PyrH belongs to group B1 and exhibits hydrogen bonds between tryptophan and the residues in Trp3, along with Trp1, -2, and Trp4 motifs (Fig. 2e). Notably, the residues positioned ahead of Trp3 (A153 to A158 in PyrH) become ordered upon complexation with tryptophan, indicating that these regions interact during substrate binding (62). Such a Trp3 region of PyrH (Q160 to Q163) exists in the alpha helix (α7) (Fig. 2g), which follows a longer loop than that observed in PrnA of group A. The side chains of Q160 and Q163 in PyrH interact with the carboxylate oxygen atom of tryptophan, and they are 3.21 and 2.88 Å apart from tryptophan, respectively (62). While Trp3 regions were not observed in the halogenases in group A, a strong signal of Trp4 was observed in alpha-helix regions (e.g., α15, α16, and α17 in PrnA; α14, α15, and α16 in Thal), which plays an important role in substrate binding. To verify the Trp3 functional site, we performed experimental characterizations (see the experiment results in the next section).

The region Trp4 shows high sequence and structural variations relative to Trp1 and Trp2. The Trp4 motif is assigned as YYxxF(E|D)(A|Y)EF(R|K)NFW(L|T)N(G|S)(N|S)YY in group A and (Y|H)YHG(L|F)(P|E)PYS in group B, whereas no analogous motif is found in group C (Fig. 2g). The structures of Trp-FDHs indicate that Trp4 motifs form enzyme-substrate interactions with tryptophan, which suggests their critical roles for substrate binding (Fig. 2a to e). This observation is consistent with the altered substrate scope and regioselectivity upon mutation of Trp4 motifs (33, 37, 39, 63). The structure of BrvH (31) is in good agreement with the observation that halogenases lacking the Trp4 motifs do not interact with tryptophan. As such, it is likely that halogenases in group C might have a different substrate scope than those in groups A and B.

### Characterization of putative Trp-FDHs.

To validate the classification of FDHs, we isolated and characterized 11 putative halogenases: 7 bacterial genomic FDHs (Hal1 to -7) and 4 metagenomic FDHs (MHal1 to -4) (Fig. 1, Table 1, and Table S2). One or two sequences were randomly selected from each phylogenetic group (groups A to C). Both Hal1 and Hal7 are located close to 7-Trp-FDHs, such as PrnA and KtzQ, respectively, whereas Hal7 is located slightly apart from the majority of 7-Trp-FDHs. This pattern is also observed in the phylogenetic tree (Fig. 1b). In contrast, Hal2 and Hal3 are tightly clustered with multiple 6-Trp-FDHs in the embedding space and reside in group A2 with 6-Trp-FDHs such as ThdH (Thal), BorH, and AORI_5336 (Fig. 1). Notably, Hal3, which was previously named ThnH and reported as a part of the gene cluster for thienodolin synthesis (56), is clustered with a 6-Trp-FDH. Hal4 and Hal5 are tightly clustered with multiple 6-Trp-FDHs in the embedding space, which are close to 6-Trp-FDHs and belong to group B2 with 6-Trp-FDHs such as SttH, KtzR, and Th-Hal (Fig. 1). Lastly, Hal6 is close to 5-Trp-FDHs in the embedding space and is in group B1 with 5-Trp-FDHs such as PyrH, SpmH, and XszenFHal in the tree (Fig. 1). Notably, the selected enzymes possess strictly conserved residues involved in FAD and halide binding, whereas Hal1 to -7 genes exhibit the representative Trp1 to -4 motifs of each group shown in the conservation graph (Fig. 2g and Table 1). MHal1 to -4 exhibit an analogous Trp1 and -2 motif but no Trp4 motif.

**TABLE 1:** Experimentally characterized FDHs^*a*^

Group	Enzyme	Regioselectivity for Trp	Trp1	Trp2	Trp3	Trp4
A1	PrnA	7	AT**IP**SL	H**L**FGN		YYETFDFEFKNFWLN**GN**YY
	Hal1	7	AT**IP**SL	H**L**FGN		YYETFDFEFKNFWLN**GN**YY
	KtzQ	7	AT**IP**NL	H**L**FGQ		YYGNFEAEFRNFWTN**SN**YY
	RebH	7	AT**IP**NL	H**S**FGL		YYGNFEEEFRNFWNN**SN**YY
	Hal7	7	ATI**P**NL	H**L**FGL		YYGNFEAEFRNFWSN**AN**YY
A2	ThdH (Thal)	6	AT**VP**NL	H**P**FGL		YYGNFEA EFRNFWTN**GS**YY
	FmoD	6	AT**VP**NL	H**P**FGL		YYGNFEAEFRNFWTN**GS**YY
	Hal3 (ThnH)	6	AT**IP**NL	H**P**FGL		YYGNFEAEFRNFWTN**GS**YY
	BorH	6	AT**VP**NL	H**P**FGL		YYGRFEAEFRNFWTN**GS**YY
	AORI_5336	6	AT**VP**NL	H**P**FGL		YYGNFDAEFRNFWTN**GS**YY
	Hal2	6	AT**VP**NL	H**P**FGL		YYDNFDVEFRNFWTN**GS**YY
B2	Thermophilic Hal	6	AT**FSDI**	H**P**FE**Q**	**Q**GKT**Q**	YYHG**LP**PYS
	KtzR	6	AT**FSDI**	Q**P**FE**Q**	**Q**GKS**Q**	YYHG**LP**PYS
	SttH	6	AT**FSDI**	H**P**FE**Q**	**Q**GKT**Q**	YYHG**LP**PYS
	Tar14	6	AT**FSDI**	H**P**FE**Q**	**Q**GA**Q**	YYHG**LP**PYS
	Hal4	6	AT**FSDI**	H**P**FE**Q**	**Q**GKT**Q**	YYHG**LP**AYS
	Hal5	6	AT**FSDI**	H**P**FE**Q**	**Q**GT**Q**	HYHG**LP**PYS
B1	ClaH	5	AT**FSTV**	H**P**FE**R**	**Q**GT**Q**	HYHG**FE**AYS		
	AbeH	5	AT**FSTV**	H**P**FE**R**	**Q**TT**Q**	HYHG**FE**SYS		
	PyrH	5	AT**FSTV**	H**P**FE**R**	**Q**RA**Q**	YYHG**FE**TYS		
	SpmH	5	AT**FSTV**	H**P**FE**R**	**Q**RA**Q**	YYHG**FE**PYS		
	XszenFHal	5	AT**FSTV**	H**P**FE**R**	**Q**RA**Q**	FYHG**FE**EYS		
	Hal6	5	AT**FSTV**	H**P**FE**R**	**Q**RA**Q**	YYHG**FE**SYS		
C	KrmI	6	ATTPSL	YPFGA		
	MibH	NA^*b*^	ATVSYM	APFDW		
	BrvH	NA	ATIPTI	HPFGL		
	VirX1	NA	STLGHF	YPFGP		
	Xcc-b100-1333	NA	STVPPI	HPFGS		
	Xcc-b100-4156	NA	ATIPSL	HSFGH		
	Xcc-b100-4345	NA	ATVPHI	HGFGT		
	MHal1	NA	ATMPNI	HSFDS		
	MHal2	NA	ATIIQM	NPFQP		
	MHal3	NA	ATVPGM	HPFNS		
	MHal4	NA	ATIPPL	HSFGD		

a Residues proposed to be important in the determination of reactivity and regioselectivity of tryptophan are shown in bold.

b NA, not applicable.

10.1128/mSystems.00053-21.7 TABLE S2 Gene sequences and primer sequences used for site-directed mutagenesis of the selected putative FDHs (Hal1 to -7 and MHal1 to -4). The homologous proteins of MHals were identified using BLASTp against a nonredundant protein database. NA, not applicable. Download Table S2, DOCX file, 0.1 MB. Copyright © 2021 Jeon et al. 2021 Jeon et al. https://creativecommons.org/licenses/by/4.0/ This content is distributed under the terms of the Creative Commons Attribution 4.0 International license.

**FIG 3:**
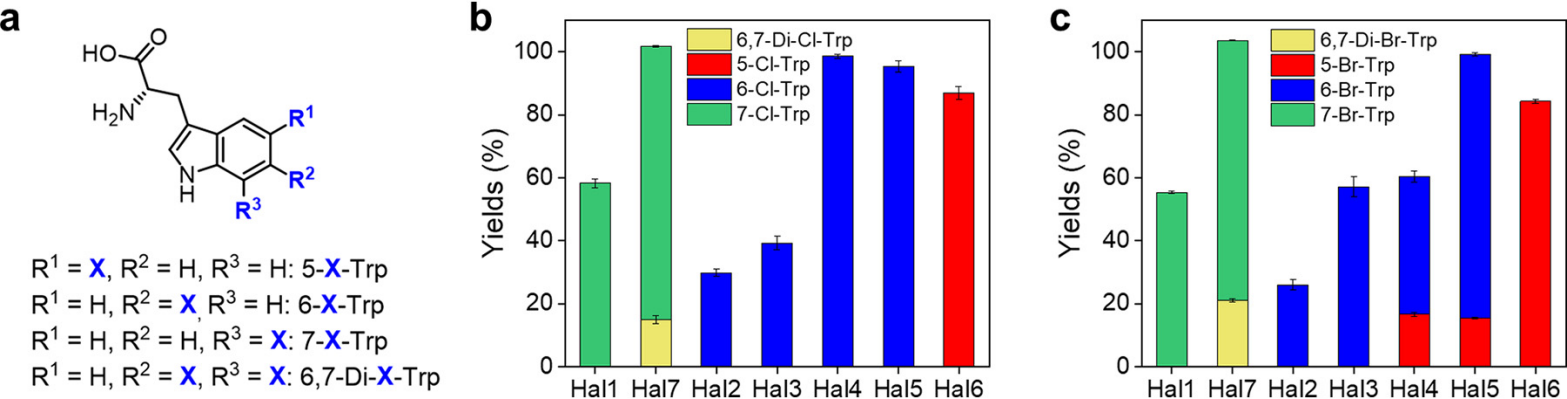
Halogenation of Trp-FDHs with tryptophan. (a) Chemical structures of the halogenated products. Product yields in chlorination (b) and bromination (c) reactions.

The putative FDHs that we identified were heterologously expressed in Escherichia coli using the methods described in reference 32. We measured steady-state activities of putative Trp-FDHs from bacterial genomes (Hal1 to -7). The disappearance of the substrate, tryptophan, and the concurrent formation of new peaks were observed for all reactions (see Fig. S3). The products were analyzed by liquid chromatography mass spectrometry (LC-MS) and ^1^H and ^13^C nuclear magnetic resonance (NMR) (Table S3; Fig. S3; Text S1). Hal1 and Hal7 primarily produced 7-chlorotryptophan (7-Cl-Trp), while Hal7 also produced 6,7-dichlorotryptophan (6,7-Di-Cl-Trp) as a minor product. Hal2 to -5 produced 6-chlorotryptophan (6-Cl-Trp), and Hal6 produced 5-chlorotryptophan (5-Cl-Trp), which is consistent with the genomic patterns and the position in the embedding space (Fig. 3a and b). Notably, these results are inconsistent with the annotations in NCBI gene database, where all selected genes, except for Hal3, are described as 7-Trp-FDHs. Such incorrect annotations might be due to the use of incorrect functional assignments based on homology transfer. Instead, the experimentally determined regioselectivity is consistent with the phylogenetic analysis (Fig. 1b), demonstrating that our bioinformatics approach correctly establishes the relationship between tryptophan-binding motifs and regioselectivity in halogenation.

10.1128/mSystems.00053-21.3 FIG S3 Characterization of halogenated products. (A) HPLC analysis of chlorination (left) and bromination (right) with (A1) tryptophan, (A2) indole, (A3) 1-naphthol, (A4) 2-naphthol, (A5) benzothiazole, (A6) benzimidazole, (A7) tyrosine, (A8) phenylalanine, and (A9) phenol. The reactions proceeded overnight. For the substrates with no reactivity, the traces from chlorination and bromination were overlaid for comparison. The standard samples are shown with asterisks. (B) ^1^H/^13^C NMR and LC/GC-MS spectra of halogenated products: (B1) 5-chloro-l-tryptophan, (B2) 5-bromo-l-tryptophan, (B3) 6-chloro-l-tryptophan ,(B4) 6-bromo-l-tryptophan, (B5) 7-chloro-l-tryptophan, (B6) 7-bromo-l-tryptophan, (B7) 6,7-dichloro-l-tryptophan, (B8) 6,7-dibromo-l-tryptophan, (B9) 3-chloroindole, (B10) 3-bromoindole, (B11) 2,3-dichloroindole, (B12) 2,3-dibromoindole, (B13) 1-chloro-2-naphthol, (B14) 1-bromo-2-naphthol, (B15) 4-chloro-1-naphthol isolated from Hal1, (B16) 4-chloro-1-naphthol isolated from Hal3, (B17) 4-bromo-1-naphthol, (B18) 4-bromophenol. Download FIG S3, PDF file, 2.9 MB. Copyright © 2021 Jeon et al. 2021 Jeon et al. https://creativecommons.org/licenses/by/4.0/ This content is distributed under the terms of the Creative Commons Attribution 4.0 International license.

10.1128/mSystems.00053-21.8 TABLE S3 Conversion and yield in the halogenation of l-tryptophan (Trp) with Hal 1 to -7, MHal1 to -4, and the variants. Numbers in parentheses indicate standard deviations. Asterisks (*) indicate inserted amino acids. The enzyme activities of Hal2 might have been underestimated due to protein instability. Download Table S3, DOCX file, 0.1 MB. Copyright © 2021 Jeon et al. 2021 Jeon et al. https://creativecommons.org/licenses/by/4.0/ This content is distributed under the terms of the Creative Commons Attribution 4.0 International license.

10.1128/mSystems.00053-21.10 TEXT S1 Preparation and biochemical characterization of putative halogenases from genomic and metagenomic sequences and characterization of halogenated products by ^1^H and ^13^C NMR spectroscopy and MS. Download Text S1, DOCX file, 0.1 MB. Copyright © 2021 Jeon et al. 2021 Jeon et al. https://creativecommons.org/licenses/by/4.0/ This content is distributed under the terms of the Creative Commons Attribution 4.0 International license.

Substitution of Cl^−^ with Br^−^ yielded comparable conversion (%) of tryptophan (Fig. 3a and c). LC-MS analysis indicated that the major products are monobrominated. The regioselectivity in bromination was maintained except for Hal4 to -5 in group B2, which produced mixtures of 5-Br-Trp and 6-Br-Trp. A lower regioselectivity here might indicate that Trp motifs in group B2 less stringently constrain the positioning of tryptophan to the catalytic lysine residue. Additionally, the more electrophilic and polarized nature of BrO^−^ over that of ClO^−^ might be partially responsible for the more reactive and less selective halogenation.

### Altered regioselectivity of Trp-FDHs by the modification of Trp1 to -4 motifs.

**FIG 4:**
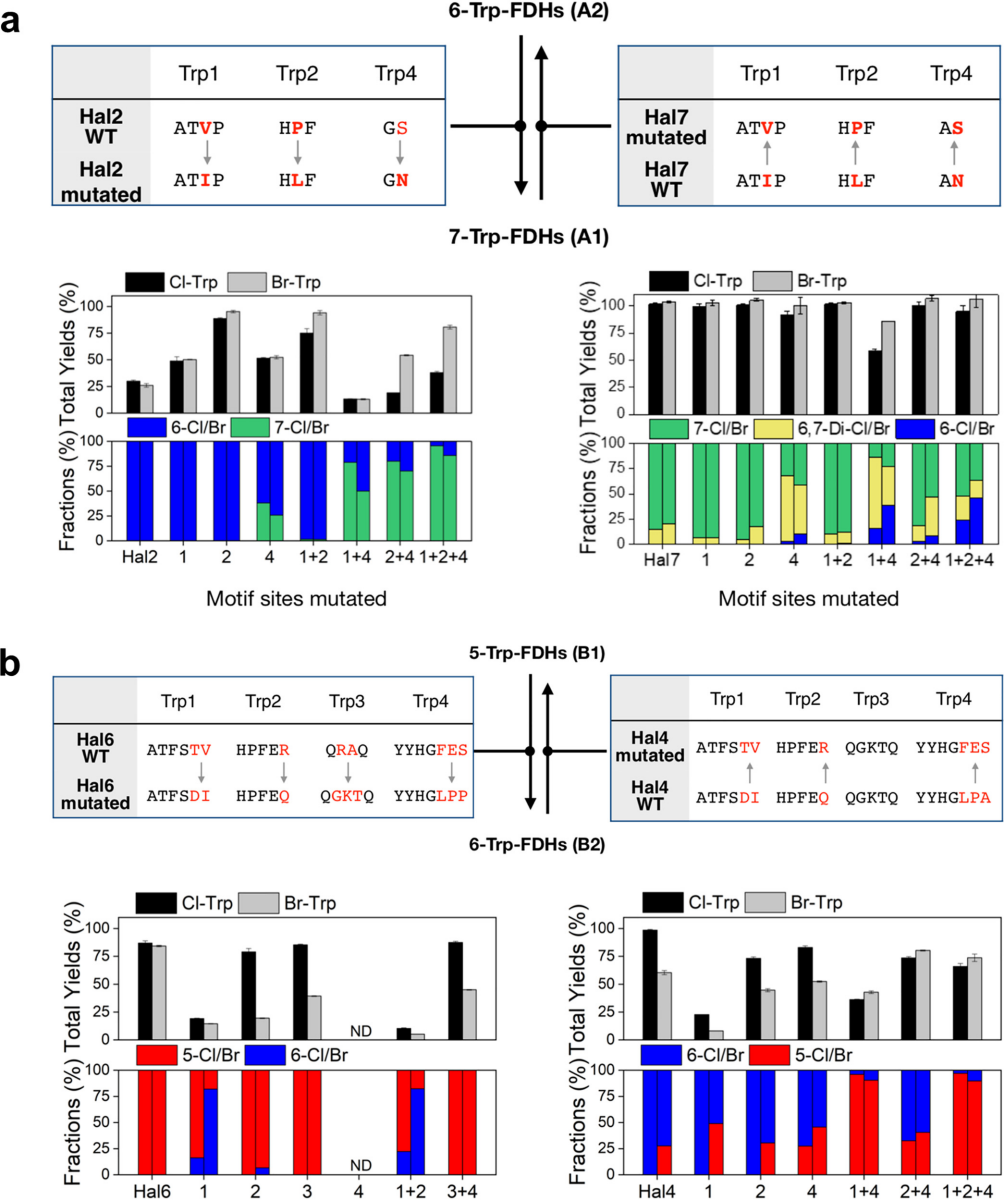
Altered regioselectivity of Trp-FDH by modification of Trp conservation sites. (a) Alteration between 6-Trp-FDH and 7-Trp-FDH. Total yields (top) and fractions (bottom) upon mutations of Hal2 to 7-Trp-FDH (left) and Hal7 to 6-Trp-FDH (right). (b) Alteration between 5-Trp-FDH and 6-Trp-FDH: Hal6 to 6-Trp-FDH (left) and Hal4 to 5-Trp-FDH (right). The fractions of the products in chlorination and bromination reactions are shown in left and right columns, respectively.

Our genomic analysis integrated with structural information indicated that the genomic patterns and the Trp1 to -4 motifs might determine the regioselectivity of tryptophan halogenation. We thus proceeded to investigate whether the discrete noncovalent interactions could be changed by site-directed mutagenesis, thus altering the regioselectivity. Key amino acids in Trp1 to -4 motifs were replaced by comparing the motifs between two groups of halogenases that differ in the regioselectivity. The first mutagenesis study was performed between 6-Trp-FDH and 7-Trp-FDH in group A (Fig. 4a). The second study was performed between 5-Trp-FDH and 6-Trp-FDH in group B (Fig. 4b).

As shown in Fig. 4a, the first mutagenesis study was performed between 6-Trp-FDH and 7-Trp-FDH in group A. For the conversion of Hal2 (6-Trp-FDH in group A2) to 7-Trp-FDH, we carried out the following mutations: ATVP to ATIP (Trp1), HPF to HLF (Trp2), and YYx_13_GSYY to YYx_13_GNYY (Trp4) (Fig. 4a). Mutation of the Trp1 or Trp2 motif alone or together yielded no detectible changes in regioselectivity (Fig. 4a). Substitution of the Trp4 motif resulted in the 1:0.6 production of 6-Cl-Trp and 7-Cl-Trp. Inclusion of Trp1 or Trp2 mutation in the Trp4 variant inverted the product distribution to 1:4. Finally, when all three mutations were combined, the ratio became 1:21, which is consistent with the recent work on the quintuple mutations of Thal (40). These data indicate that seemingly subtle mutations, such as Val to Ile (Trp1), Pro to Leu (Trp2), and Ser to Asn (Trp4), are responsible for regioselectivity, and the orchestration of these motifs is necessary to alter the positioning of tryptophan exclusively. These results also indicate that Trp1, Trp2, and Trp4 motifs can be utilized as effective sequence markers to distinguish 6-Trp-FDH from 7-Trp-FDH proteins. Similar effects of these mutations were observed in the bromination with Hal2 (Fig. 4a). Again, the Trp4 motif makes the largest contribution, and the highest conversion to 7-Br-Trp is observed when Trp1, Trp2, and Trp4 mutations were combined, producing 6-Br-Trp and 7-Br-Trp in a 1:6 ratio. The distribution of the brominated products was not as prominent as that with Cl^−^, which is consistent with our earlier observation that BrO^−^ is less selective than ClO^−^.

The reverse mutagenesis was performed with Hal7 (7-Trp-FDH in group A1) toward 6-Trp-FDH. The effects of swapping the Trp motifs were similar to those with the A2 protein (Fig. 4a). While Trp1 and/or Trp2 mutations yielded no or slight perturbation, the addition of the Trp4 mutation drastically produced monohalogenated products at the 6 position in detectible amounts. When Trp1, Trp2, and Trp4 mutations were combined, the relative fraction of 6-Cl/Br-Trp was maximized, yielding 7-Br-Trp, 6,7-Di-Br-Trp, and 6-Br-Trp in a 1:0.46:1.23 ratio. We suspect that the 6,7-dihalogenated products were formed presumably due to the loosely regulated regioselectivity. Subsequent growth and decay upon the series of mutations might indicate that more selective interactions are made with tryptophan.

The second mutation study was carried out between 5-Trp-FDH and 6-Trp-FDH in group B (Fig. 4b). They possess identical Trp1 and Trp2 motifs as ATFS and HPF, respectively. However, the tilted orientations of the conserved phenylalanines and a histidine were markedly different in the structures of PyrH (5-Trp-FDH in B1) and SttH (6-Trp-FDH in B2), thereby altering the directionality of the π-π stacking interactions. We speculated that the amino acids subsequent to the Trp1 and Trp2 motifs might be responsible for these distinct interactions. We thus extended the Trp1 and Trp2 motifs to include two additional amino acids: ATFSTV and HPFER in group B1 and ATFSDI and HPFEQ in group B2 (Fig. 2g and Table 1). Additionally, two glutamine residues in Trp3 (QxxQ in group B1 and QxxxQ in group B2) and Trp4 motifs (FE[T|S] in group B1 and LP[P|A] in group B2) were taken into consideration.

Since the role of Trp3 regions has not been investigated thus far, we first carried out experiments to confirm their involvement in the halogenation reaction. A double mutant was prepared with Hal6 by replacing both glutamines in Trp3 with alanines (from QxxQ to AxxA). Significantly lower conversion of tryptophan was observed in both chlorination and bromination reactions (see Table S3). The results suggest that two glutamine residues in Trp3 play an essential role in the reaction with tryptophan, possibly by the formation of hydrogen bonds with the amino and carboxyl moieties of the substrate.

To show the conversion from 5-Trp-FDH to 6-Trp-FDH in group B, the following mutations were made with Hal6 (5-Trp-FDH in group B1): ATFSTV to ATFSDI (Trp1), HPFER to HPFEQ (Trp2), QRAQ to QGKTQ (Trp3), or FES to LPP (Trp4) (Fig. 4b). Modification of Trp1 slightly altered the product distribution of 5-Cl-Trp and 6-Cl-Trp, whereas the ratio of brominated products was substantially changed to 1:4.6 (Fig. 4b). However, mutations of either the Trp2 or Trp3 motif reduced the overall yields without considerable changes in regioselectivity. Trp4 mutation alone or combined with a Trp1 or Trp2 mutation completely inactivated the enzyme, which is consistent with previous studies on PyrH (33). However, enzymatic activity was restored by the introduction of a Trp3 mutation without altering regioselectivity. When Trp1 and Trp2 mutations were combined, the ratios of 5-Cl-Trp to 6-Cl-Trp and of 5-Br-Trp to 6-Br-Trp were 1:0.3 and 1:4.7, respectively.

In the reverse mutagenesis performed with Hal4 (6-Trp-FDH in group B2) toward 5-Trp-FDH in group B1, the regiochemical preference was improved. While Trp1 or Trp2 mutations of Hal4 (6-Trp-FDH in group B2) alone yielded no alternation in the distribution of chlorinated products, the Trp4 mutation by itself altered the ratio of 6-Cl-Trp and 5-Cl-Trp to 1:0.4 (Fig. 4b), which is consistent with a previous report on the triple mutant of SttH (33). Substitutions on both Trp2 and Trp4 motifs resulted in negligible changes, whereas substitutions on Trp1 and Trp4 enhanced the ratio to 1:25. When Trp1, Trp2, and Trp4 motifs were altered simultaneously, the ratio was increased to 1:34 in chlorination, indicating that the combined interactions of these three motifs with tryptophan dictate regioselectivity in a nearly exclusive manner. The effects of this mutation were conserved in the bromination reaction except that Trp1 mutation alone induced considerable changes in the regioselectivity. Trp4 mutation produced mixed products at the ratio of 1:1; the combination of Trp1, Trp2, and Trp4 mutations maximized the ratio to 1:9.

### Biochemical characterization of metagenomic FDHs.

Steady-state activity assays were carried out on MHal1 to -4. MHal2 to -4 actively halogenated indole, but no conversion was observed for tryptophan (Tables S3 and S4; Fig. S3; Text S1). The discrete reactivity indicates that Trp-binding site Trp4, which is absent in MHal1 to -4, is critical for the halogenation activity toward tryptophan. Notably, MHal2 to -4 were considerably more reactive in bromination than in chlorination, although there is no significant difference in the sequences and structures of FAD- and halide-binding sites in genomic and metagenomic FDHs. Similarly to that for genomic FDHs, the molecular basis for halide selectivity observed in MHals needs further investigation. The discrete chemical nature of BrO^−^ and ClO^−^ and/or kinetic and/or thermodynamic parameters in transfer reactions of hypohalite to tryptophan or the indole-binding region via catalytic lysine might be in part responsible for the determination of the reactivities. The latter is consistent with the molecular dynamics (MD) simulation studies of PrnA and PyrH (64), where significant conformational changes occur in the transfer of hypohalite. MHal1 was inactive even with indole, which is presumably related to the substantially altered sequence nearby the Trp-binding region (see Fig. S4).

10.1128/mSystems.00053-21.4 FIG S4 Structure and sequence analysis of metagenomic halogenases (MHals). (A) Structure modeling of MHal1 and MHal2. Tryptophan-binding site in MHal1 is possibly occluded by the presence of amino acids, colored blue. (B) Sequence alignments of metagenomic halogenases. The additional sequences shown in panel A are indicated with a blue dotted box. Download FIG S4, TIF file, 2.2 MB. Copyright © 2021 Jeon et al. 2021 Jeon et al. https://creativecommons.org/licenses/by/4.0/ This content is distributed under the terms of the Creative Commons Attribution 4.0 International license.

10.1128/mSystems.00053-21.9 TABLE S4 Substrate scopes of the selected genomic and metagenomic FDHs. Chemical structures of chlorinated (a) and brominated (c) products. Conversion (%) and yield (%) in the chlorination of indole and other aromatic substrates with NaCl (b) and NaBr (d). Numbers in parentheses indicate standard deviations. (e) No conversion of benzothiazole, benzimidazole, phenylalanine, and tyrosine was observed for all putative FDHs. Due to poor stability of Hal2, conversion and yield other than indole were not determined. ND, not determined. Download Table S4, DOCX file, 0.1 MB. Copyright © 2021 Jeon et al. 2021 Jeon et al. https://creativecommons.org/licenses/by/4.0/ This content is distributed under the terms of the Creative Commons Attribution 4.0 International license.

**FIG 5:**
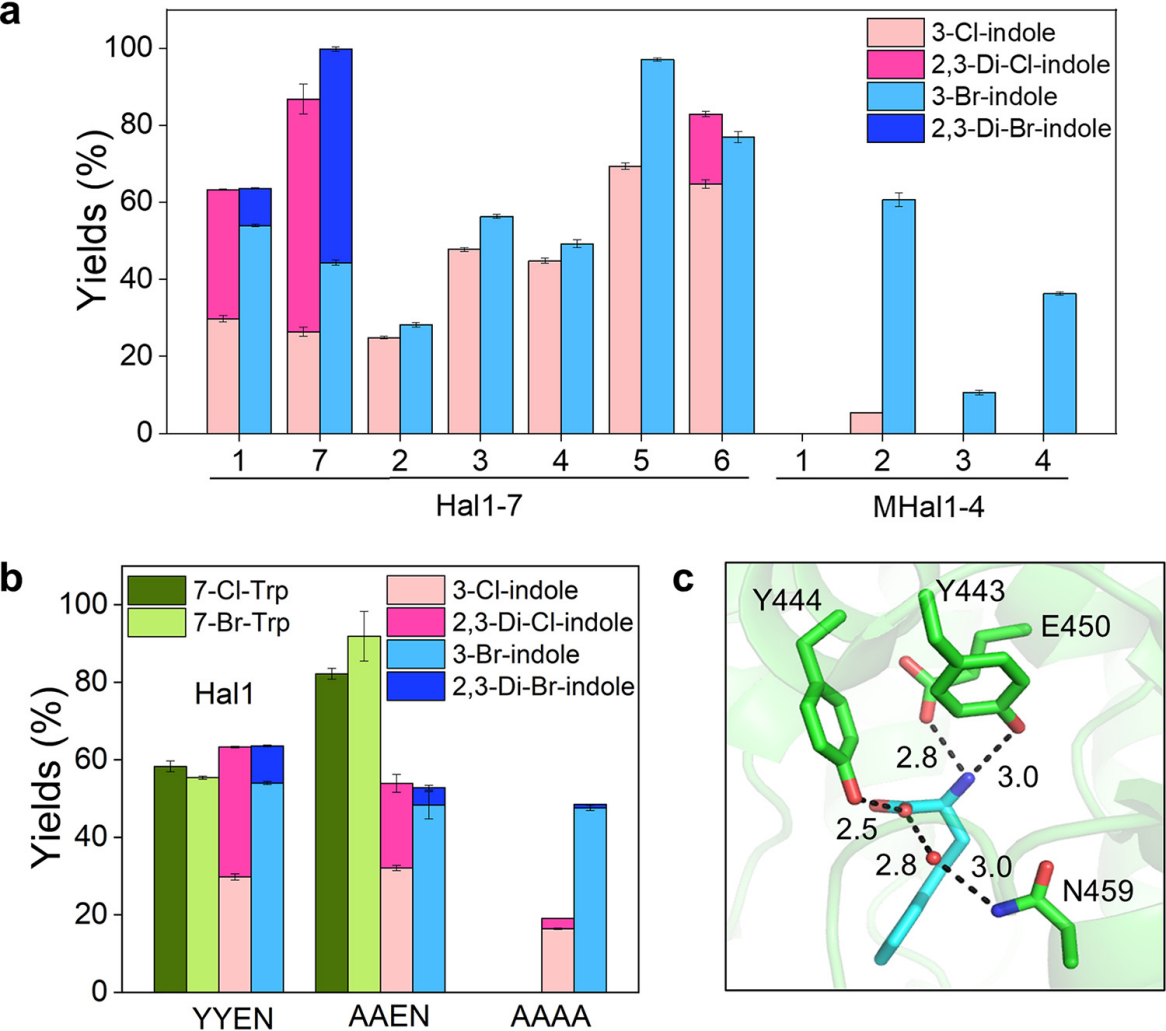
Halogenation of bacterial and metagenomic FDHs with indole. (a) Yields (%) of chlorinated and brominated indoles. (b) Reactivities of Hal1 variants at the Trp4 motif (YYEN) with tryptophan and indole. (c) Hydrogen-bonding interactions between Trp4 residues (YYEN) and a tryptophan shown in the structure of PrnA in A1 (2AQJ).

For the reactions of MHal2 to -4 with indole, they exhibited identical regioselectivity, exclusively producing 3-Cl and 3-Br-indoles. We also measured the reactivities of Hal1 to -7 with indole. They also produced 3-Cl and 3-Br-indoles, displaying the regioselectivity identical to that of MHal2 to -4 (Fig. 5a). Additionally, Hal1, Hal6, and Hal7 yielded doubly halogenated products from indole, i.e., 2,3-dichloroindole or 2,3-dibromoindole. The formation of doubly halogenated products indicates that the binding affinity and additional halogenation reactivity of 3-Cl-indole or 3-Br-indole might be at least comparable to those of indole.

As the distinction between Hal1 to -7 and MHal2 to -4 is related to the Trp4 motif, we also explored whether the sequences in Trp4 may determine the reactivity with tryptophan versus that with indole. When two tyrosine residues (YY) at the beginning of Trp4 motif in Hal1 were mutated to alanines (Fig. 5b and c), the mutant yielded even higher conversion of tryptophan. When the other conserved residues in Trp4, glutamate and asparagine (EN), were mutated to alanines, the quartet mutant became completely inactive to tryptophan. Double or quartet mutants produced halogenated indoles with comparable or lower yields than Hal1, indicating that the absence of these four residues might severely weaken substrate-binding affinity, leading to lower catalytic activities, particularly for tryptophan but not as much as with indole. Therefore, the presence of the Trp4 motif might be critical to determining the substrate scopes, such as tryptophan versus indole, and the product yields.

**FIG 6:**
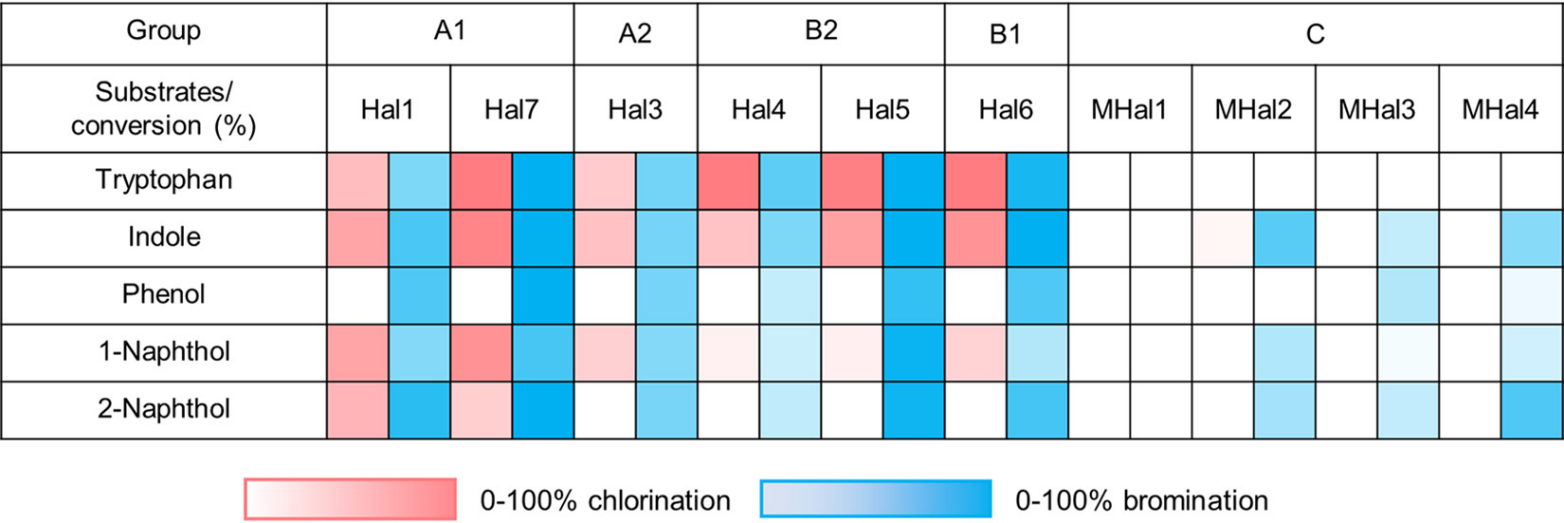
Catalytic activities of Trp-FDH identified from the bacterial genomes and metagenomes on diverse aromatic substrates.

We further investigated the substrate scopes of Hal1, Hal3 to -7, and MHal1 to -4 beyond indole (Fig. 6; Table S4; Fig. S3; Text S1). Hal2 was excluded due to protein instability. First, we observed whether other aromatic amino acids, such as tyrosine and phenylalanine, can be halogenated. No reactivity was observed, implying that the sp^2^ carbon of aromatic rings might not be closely placed to the catalytic lysine. It is possible that the relatively smaller size of aromatic rings might have altered the mode of binding and interactions with halogenases. When indole derivatives, such as benzimidazole and benzothiazole having a heteroatom such as S and N at the C-3 position of indole, respectively, were added, no halogenated product was again observed, indicating that an alternative mode of binding of the indole moiety is disallowed, which contrasted with the discrete regioselectivity of tryptophan. Phenol was brominated by most of the proteins that exhibited activity with indole (Hal1, Hal3 to -7, and MHal3 and -4). The genomic and metagenomic FDHs were also active with 1-naphthol and 2-naphthol. Hal1 and Hal3 to -7 yielded both chlorinated and brominated products (11% to 83% and 21% to 100% conversion, respectively), whereas MHal2 to -4 reacted with 1- and 2-naphthols only in the presence of Br^−^ (4% to 69% conversion). Notably, irrespective of the phylogenetic positions of the genes, only the *ortho* and *para* positions of phenol and naphthols were halogenated, which reflects a strong intrinsic electronic bias for regioselectivity not overridden by substrate-protein interactions (Fig. 1b). Our results reveal that FDHs can act on diverse substrates, including a 6-membered benzene ring (phenol), 5-membered ring fused with 6-membered ring (indole), and two fused 6-membered rings (naphthol), with substantial variations in conversion and product distribution.

## DISCUSSION

Many studies have performed computational analyses to estimate the functions of the genes in newly assembled genomes. For this purpose, sequence searches have been extensively used to find homologous genes of the same function. However, there exists limitations in finding subtle differences in biochemical reactivities such as regioselectivity in functional group transformation and selectivity of substrates. Since halogenation is an important chemical reaction in pharmaceutical or agrochemical applications, we have carried out an integrated genomic and protein structural analysis to identify the genotypes that are critical to the biochemical functions of FDHs. From both genome and metagenome mining, halogenases with similar genomic patterns of active sites are clustered in the phylogenetic tree as well as embedding space.

In this study, we found primarily genomic patterns that determine the enzymatic activity toward tryptophan (Trp-FDH versus non-Trp-FDH) and the site of halogenation for tryptophan (5-, 6-, and 7-Cl/Br-Trp-FDH). Recent studies found that the sequences with different substrate scope were clustered based on the sequence similarity in the network analysis (19) and screened the halogenation with various substrates (65). Structure analysis of the known proteins identified the residues that bind to FAD and substrates (66–69). Compared with such studies, our work utilizes large-scale genomic information to propose a comprehensive set of active sites. Such sequence motifs for the active sites showed different patterns for the discrete regioselectivity of Trp-FDHs. Notably, the sequence motifs of Trp-FDHs that belong to A1 and A2 differ only moderately while they exhibit discrete regioselectivity. The results suggest that the halogenating reactivities are determined by the actions of several nonsequential and subtly different sequence motifs in concerted and complicated manners. In conjunction with biochemical assays, mutagenesis experiments suggest that the sequence motifs can be exchanged to create altered product distributions, implicating potential biotechnological applications in protein engineering and synthetic biology for the synthesis of novel natural products.

The correlation of the substrate specificity and regioselectivity with source organisms and their habitats is also an interesting and challenging question to characterize FDHs. In terms of taxonomic distribution, the FDHs were found in *Actinobacteria*, *Cyanobacteria*, and *Proteobacteria* (see Table S1 in the supplemental material). Since we do not have enough FDHs identified, it is hard to determine the correlation between substrate specificity/regioselectivity and taxonomic distribution. In this study, we used seawater microbiome data to find more diverse FDHs and found that most of the sequences were indole-FDHs. Screening of FDHs in more diverse environments could provide a better chance to characterize FDHs. The demonstration of discrete and diverse substrate scopes of genomic and metagenomic FDHs suggests that the FDHs annotated in the last decade are only a small portion of the genes, and more FDH genes could be discovered.

As discussed above, many gene annotations are based primarily on the homology search, which is fast but less accurate to predict subtle functional differences. While the NCBI annotation reported six FDHs (Hal1 and -2 and Hal4 to -7) as 7-Trp-FDHs, we found that four genes are in fact 5- or 6-Trp-FDH. To assign subtle functions such as regioselectivity, the genomic sequences of halogenases in the public repository need additional confirmation procedures using more informative models such as motif patterns or phylogenetic characteristics.

## MATERIALS AND METHODS

### Construction of Trp-FDH pHMM and halogenase identification.

To search halogenase genes in the bacterial genomes and metagenomes, the pHMM was constructed using 13 Trp-FDHs experimentally validated in previous studies (see Fig. S1 in the supplemental material): AbeH (AEF32095.1), ClaH (AEO12707.1), PyrH (AAU95674.1), KtzR (ABV56598.1), SttH (ADW94630.1), Th-Hal (WP_023586065.1), ThdH (ABK79936.1), BorH (AGI62217.1), FmoD (BAP16687.1), AORI_5336 (AGM07919.1), KtzQ (ABV56597.1), PrnA (AAB97504.1), and RebH (CAC93722.1). A multiple-sequence alignment was conducted using Clustal Omega (v1.2.4) (70). The pHMM of halogenase genes was constructed using hmmbuild in HMMER3.0 (ver. 3.1b2) (71). To identify the genes that are homologous to the known Trp-FDHs in bacterial genomes and metagenomes, hmmscan in HMMER3.0 (71) was used with the −domtblout option. Halogenases identified in the BGC cluster were included regardless of the E value. Using an in-house Python script, homology search results were filtered. The models and script are available at GitHub site (https://github.com/DMnBI/Halogenase). The E value threshold was set to <10^−130^, and the model length covered >50%. A total of 351 sequences were collected from the NCBI repository by searching with the keyword “tryptophan n-halogenase [All Fields] AND bacteria [filter],” where *n* = 5, 6, and 7. Partial and redundant sequences were discarded, resulting in 333 sequences obtained (Fig. S1). In addition, we found three additional Trp-FDHs from the literature survey, which were also included in the motif analysis.

### Detection of halogenase genes in the BGCs.

To identify BGCs in 2,787 bacterial complete genomes obtained from the NCBI repository, antiSMASH3.0 (72) was used. In total, 10,166 BGCs were predicted; halogenases inside a BGC were characterized subsequently. In total, 20 putative halogenases inside BGCs and 83 outside BGCs were classified.

### Sample collection, preparation, and metagenome shotgun sequencing.

A total of 17 metagenomic samples were obtained from different marine environments, such as coastal and deep-sea sediments (Table S1). The metagenome data sets analyzed in this study were collected in our previous studies and downloaded from the European Nucleotide Archive with the accession number ERP107268 (Table S1). All reads were 151-bp paired-end reads. Low-quality reads, which have the ambiguous sequence “N,” were trimmed and filtered using Sickle (downloaded from https://github.com/najoshi/sickle) with the option of Phred quality score of >20. The metagenomic reads were assembled using MEGAHIT (v1.0.3) (73) with default k-mer options. The assembled contigs longer than 1,000 bp were translated into protein sequences using FragGeneScan (v1.19) (74).

### Embedding of halogenase genes and classifying halogenases.

To investigate the similarity of genomic patterns among FDHs, *t*-distributed stochastic neighbor embedding (t-SNE) was carried out using “Rtsne” in the R package (75) with the parameters of perplexity of 10 and iterations of 1,000. From multiple-sequence alignment of the known halogenase sequences, the profiles were constructed for six groups (5-, 6-, and 7-Trp-FDHs, indole-Hals, phenolic-Hals, and pyrrole-Hals). Using these profiles, the probability of the amino acid at each position of the halogenase genes was calculated as a feature value to learn their embedding. After obtaining embedding vectors for each halogenase, the class (5-Trp-FDHs in B1, 6-Trp-FDHs in B2 and A2, 7-Trp-FDHs in A1, and indole-Hals in C) was assigned using the k-nearest neighbor method with a k of 1.

### Phylogenetic analysis.

Multiple-sequence alignments were conducted using MUSCLE (v3.8.31) (76), and a phylogenetic tree was constructed using MEGA6.0 (77). The evolutionary distance was calculated using the maximum likelihood method based on a Jones-Taylor-Thornton (JTT) matrix-based model. In the phylogenetic analysis, a total of 180 sequences (of 313) were used for better representation of the tree (Fig. S1). Among the 109 sequences from the keyword search, 6 were included. Among the 68 sequences obtained from metagenome data, 38 sequences were included by random sampling.

### Examination of FDH structures.

Three-dimensional structures of FDHs cocrystallized with native substrates such as FAD, halide, and/or tryptophan were obtained from the RCSB database and inspected for structural analysis. The figures were created using PyMOL software (78). The residues involved in direct enzyme-substrate interactions with the native substrates were manually examined to correlate the structural data with the sequence analysis (Table 1). Residues that form enzyme-substrate interactions were identified as FAD1 to -4, halide, and Trp1 to -4 (Fig. S2B to H). To represent the conservation of functional regions, the sequence logo was constructed using WebLogo (v.3.6.0) (79) with default options.

### Biochemical characterization of putative halogenases from genomic and metagenomic sequences.

From the sequence analysis data, we selected 11 putative FDH genes, 7 from genome data (Hal1 to -7) and 4 from metagenome data (MHal1 to -4). For steady-state coupled assays, an NADH-dependent reductase (MR) was selected from the metagenome with MHal4. The MR is functionally analogous to NAD(P)H-dependent flavin oxidoreductase (Fre) found in Escherichia coli (80).

The 12 genes were synthesized after codon optimization for E. coli heterologous expression (General Biosystems, NC Morrisville). MHal1 to -3 were inserted into the pET-22b(+) vector using NdeI and XhoI as the cut sites. MR, Hal1 to -7, and MHal4 were inserted into the pET-28a(+) vector using NdeI and BamHI as the cut sites. A stop codon was inserted at the end of the sequence due to its native C-terminal His tag sequence in the pET-28a(+) vector. The plasmids were transformed to DH5α or BL21(DE3) strains of E. coli competent cells by heat shock method for sequencing or protein expression, respectively. All putative FDHs were coexpressed with GroES and GroEL chaperones using pGro7 plasmid (TaKaRa Bio) as reported previously (32). For site-directed mutagenesis of FDHs, custom-made primers were used for PCR (Table S2). Detailed experimental procedures for cell growth, protein isolation, purification, and characterization are available in Text S1 and Fig. S5.

10.1128/mSystems.00053-21.5 FIG S5 Preparation and characterization of FAD-dependent halogenases and NADH-dependent reductases from E. coli (Fre) and metagenomes (MR). (A) Mass spectrum of FAD as isolated with Hal1. Calculated for FAD [M + H]^+^, 786.1635; observed, 785. (B) Representative trace of Hal1 purified by Ni-nitrilotriacetic acid (NTA) affinity chromatography. (C) SDS-PAGE of Hal1 (60 kDa). (D) UV-visible (Vis) spectrum of Hal1. Hal1 is copurified with FAD cofactor. (E) Purification of Fre with anionic exchange (left) and size exclusion chromatography (right). The asterisk indicates the fraction of Fre. (F) Purification of MR with Ni-NTA affinity chromatography. (G) SDS-PAGE of Fre (26.1 kDa) and MR (19.6 kDa). (H) UV-Vis spectrum of MR as isolated. (I) LC-MS of flavin mononucleotide (FMN) as coisolated with MR. Calculated for FMN [M + H]^+^, 457.112; observed, 457.0. (J) Conversion (%) of indole when Fre or MR was employed as an NADH-dependent reductase for the bromination by MHal2. (K) Consumption of NADH monitored by the absorption changes at 340 nm when either Fre or MR was used as a reductase. Download FIG S5, TIF file, 0.8 MB. Copyright © 2021 Jeon et al. 2021 Jeon et al. https://creativecommons.org/licenses/by/4.0/ This content is distributed under the terms of the Creative Commons Attribution 4.0 International license.

### Characterization of an NADH-dependent reductase from arctic environments.

The catalytic activity of a newly discovered reductase (MR) was determined by monitoring the consumption rates of NADH by the addition of 5 mM NADH, 10 μM FAD, 5 μM reductase, and 65 mM halide source, such as NaCl and NaBr. The conversions (%) of indole were measured using high-pressure liquid chromatography (HPLC) by calculating the residual indole in the presence of 5 mM NADH, 10 μM FAD, 5 μM reductase, 65 mM NaBr, and 25 μM MHal2 in 25 mM HEPES (pH 7.4) buffer at room temperature. For preliminary studies, we measured the activity of another reductase, Fre (Fig. S5) (81).

### Activity assays of putative FDHs.

For steady-state coupled activity assays, NADH (5 mM, 20 eq to the aromatic substrate), FAD (10 μM, 0.04 eq), and aromatic substrates (250 μM, 1 eq), NaCl or NaBr (65 mM, 260 eq) in 25 mM HEPES buffer, pH 7.4, were prepared in a 1.5-ml microcentrifuge tube at final concentrations. The reaction was initiated by adding putative FDH (25 μM, 0.1 eq) and MR (5 μM, 0.02 eq) to the premixed solution to make the final volume 100 μl. The assays were carried out at room temperature in darkness. After 5 h, 100 μl of methanol was added, and the resulting precipitates were removed by centrifugation at 15,928 × *g* for 10 min at 4°C. After syringe filtration, the supernatant was analyzed by reverse-phase HPLC (Agilent 1260 Infinity II) with InfinityLab Poroshell column (120 EC-C_18_, 4.6 by 100 mm, 2.7 μm, or 120 EC-C_18_ 4.6 by 150 mm, 2.7 μm) with a linear gradient of H_2_O containing 0.05% trifluoroacetic acid (TFA) and acetonitrile (ACN) containing 0.1% TFA. A representative HPLC trace and the retention time are shown in Fig. S3. Averages and standard deviations of activity assays were obtained from triple replications. The conversions and yields are listed in Tables S3 and S4.

### Characterization of the halogenated products.

For NMR characterizations, we scaled up the reaction volumes to 90 ml without altering the concentrations or the ratios of the reaction components. We observed that increasing the volume of the reactions considerably increased the total yields of the products, presumably because the activities of coupled enzymatic reactions are dependent on the concentration of dissolved dioxygen in the buffer solution. To maximize the yields, the reaction mixtures were incubated overnight. Depending on the substrates, the workup procedure was slightly modified. For the reactions with tryptophan, 90 ml of methanol (MeOH) containing 0.1% TFA was added for quenching. After centrifugation at 2,935 × *g* for 10 min at 4°C and syringe filtration, the solution was directly injected to the semiprep HPLC (ZORBAX SB-C_18_ semipreparative 9.4- by 250-mm 5-μm column). For the reactions with substrates other than tryptophan, ca. 5 drops of concentrated HCl were added for quenching. Precipitates were removed after centrifugation at 2,935 × *g* for 10 min at 4°C, and the supernatants were extracted with dichloromethane (DCM). After vacuum evaporation, ACN-H_2_O (1:9 [vol/vol]) was added to the extracted fraction. The solution was filtered by a 0.25-μm syringe filter and purified by HPLC (ZORBAX SB-C_18_ semipreparative 9.4- by 250-mm 5-μm column).

The reaction products of non-amino acidic substrates (indole, phenol, 1-naphthol, and 2-naphthol) were characterized by gas chromatography-mass spectrometry (GC-MS) (Hewlett Packard HP 6890 series GC system equipped with Agilent 5973 Network mass selective detector or Agilent Technologies 7820A GC system equipped with Agilent Technologies 5977E MSD). The reaction products from tryptophan were analyzed by LC-MS (Agilent Technologies 1200 Infinity series equipped with Agilent Technologies 6120 quadrupole LC-MS). The incorporation of chlorine or bromine was determined by the isotope ratios of ^35^Cl to ^37^Cl of 3:1 or ^79^Br to ^81^Br of 1:1, respectively. The products were analyzed by ^1^H and ^13^C NMR spectroscopy (Fig. S3; Text S1).

### Synthesis of 3-chloroindole and 3-bromoindole.

To isolate large quantities of 2,3-dichloroindole and 2,3-dibromoindole for ^1^H and ^13^C NMR experiments, we synthesized 3-chloroindole or 3-bromoindole and used as the substrate for the reactions with Hal1. To a dried 50-ml round-bottom flask, 588 mg of indole (1 eq) and 377 μl of 1,4-dimethylpiperazine (0.558 eq) were dissolved into 5 ml DCM. The temperature was lowered by placing the flask in iced water. After 10 min, 800 mg of *N*-chlorosuccinimide or *N*-bromosuccinimide (0.9 eq) was added slowly and stirred for 15 min to yield 3-chloroindole or 3-bromoindole, respectively. After 15 min, the temperature was raised to room temperature, and the reaction mixture was stirred for 3 h. Volatile fractions were removed by rotary evaporation, and the product was isolated by silica chromatography (ethyl acetate-hexane, 5:95). The isolated yields of 3-chloroindole and 3-bromoindole were 55.5% and 57%, respectively.
