# Supplementary figures and images for: Genomic Determinants Encode the Reactivity and Regioselectivity of Flavin-Dependent Halogenases in Bacterial Genomes and Metagenomes
Source: mSystems. 2021 May 27;6(3):e00053-21. doi: 10.1128/mSystems.00053-21 (PMC8269204; doi:10.1128/mSystems.00053-21)

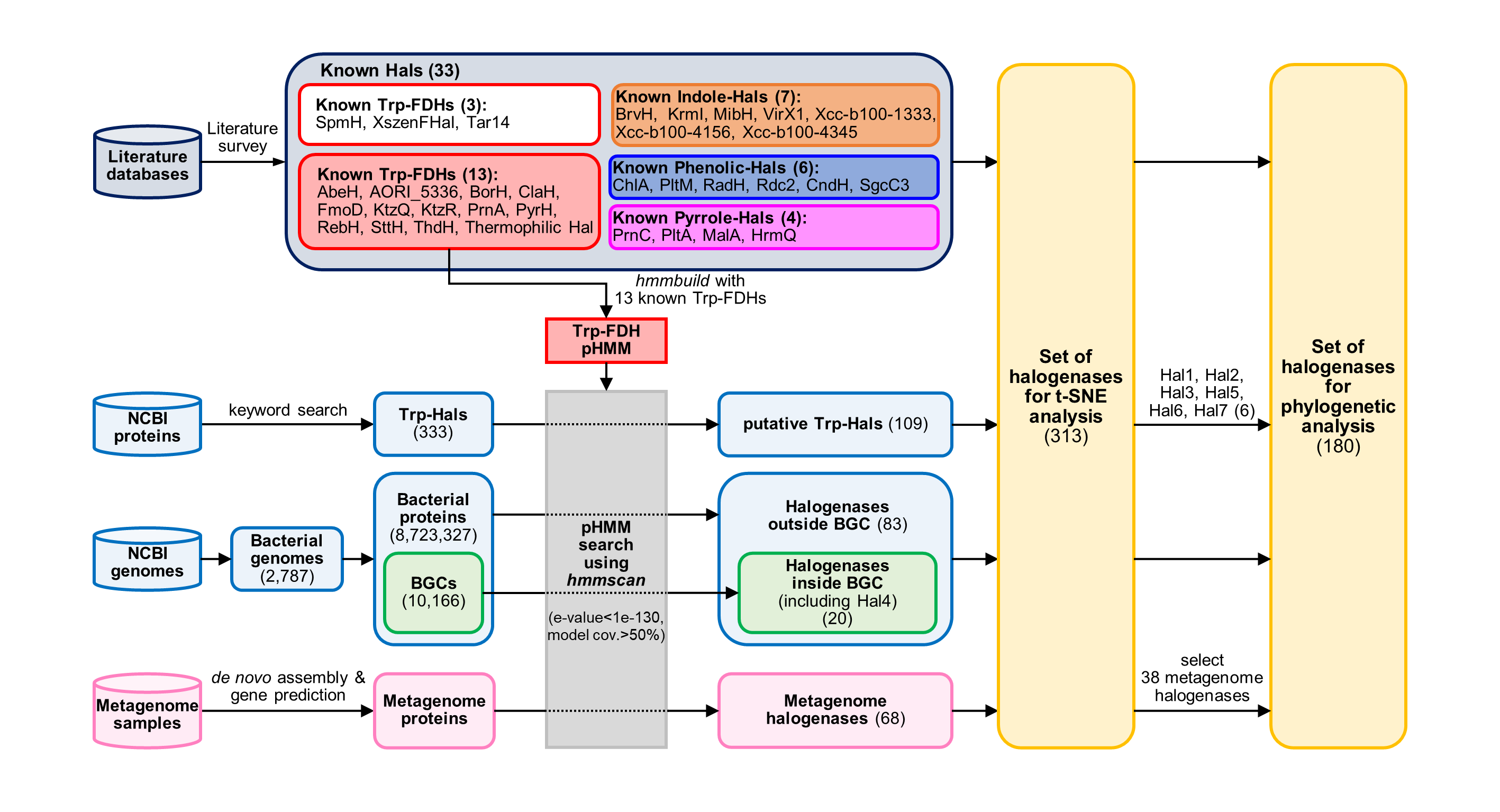

Supplement: FIG S1 [file msystems.00053-21-sf001.tif]

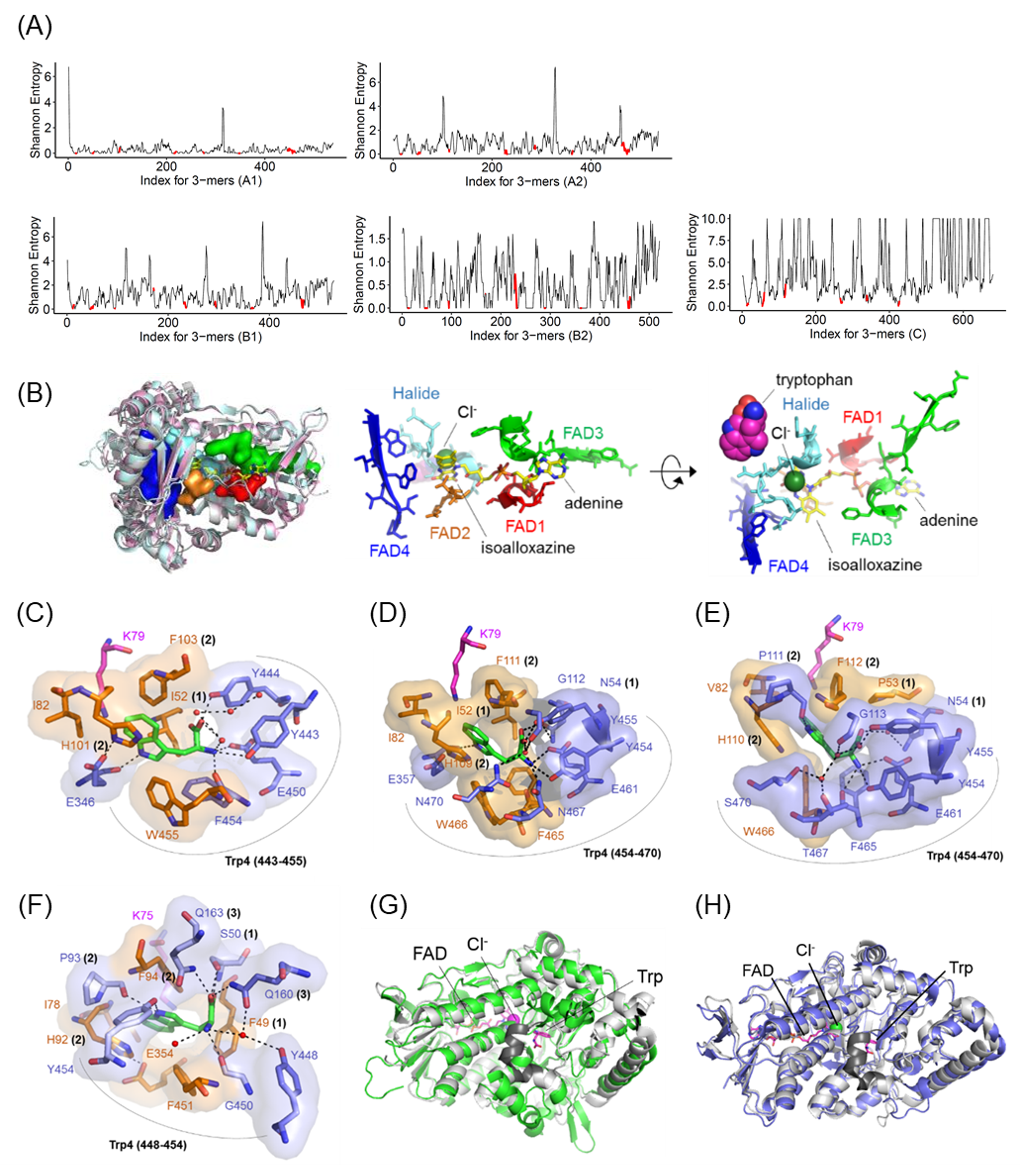

Supplement: FIG S2 [file msystems.00053-21-sf002.tif]

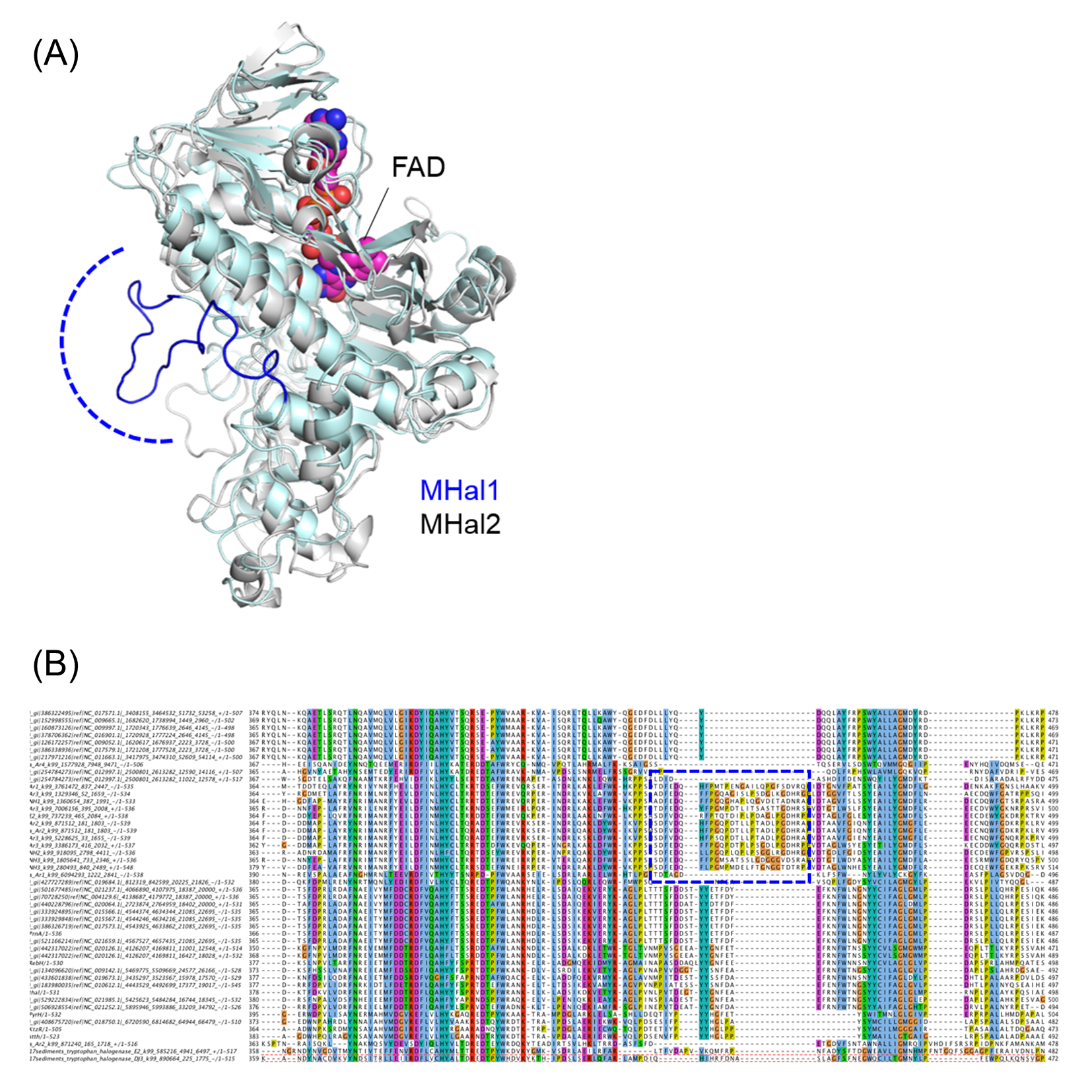

Supplement: FIG S4 [file msystems.00053-21-sf004.tif]

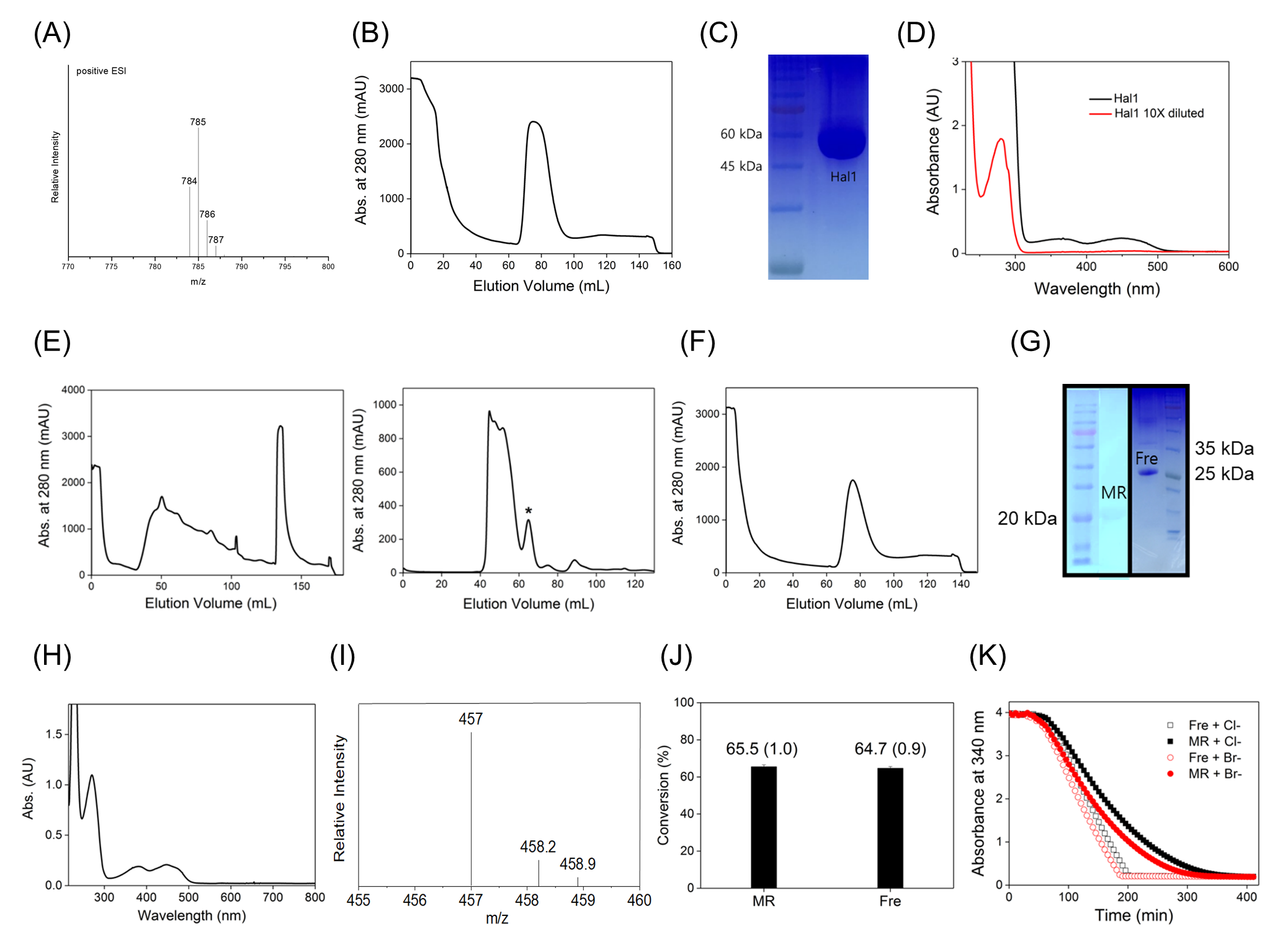

Supplement: FIG S5 [file msystems.00053-21-sf005.tif]
