# Supplementary material for: Genomic Determinants Encode the Reactivity and Regioselectivity of Flavin-Dependent Halogenases in Bacterial Genomes and Metagenomes
Source: mSystems. 2021 May 27;6(3):e00053-21. doi: 10.1128/mSystems.00053-21 (PMC8269204; doi:10.1128/mSystems.00053-21)
Supplement: TABLE S1 [file msystems.00053-21-st001.docx]

**Table S1.** Information for the metagenomic samples and halogenases that were used in training and identified in this study. (A) List of 17 metagenome samples of sediment microbiome. (B) The 33 known halogenases previously identified. (C) The 109 putative halogenases identified from the NCBI protein database. (D) The 20 putative halogenases that were identified from bacterial complete genomes and inside BGCs. (E) The 83 putative halogenases that identified from bacterial complete genomes and outside BGCs. (F) The 68 putative halogenases identified from 17 sediment microbiomes.

(A)

| Sample | Description | Depth (m) | Seq. Date | Latitude | Longitude |
| --- | --- | --- | --- | --- | --- |
| NH1 | Wando | 0 | 2014-09-22 | 34°21'30.9"N | 126°43'58.2"E |
| NH2 | Yeosu | 0 | 2014-09-22 | 34°41'14.6"N | 127°37'27.5"E |
| NH3 | Yeosu | 0 | 2014-09-22 | 34°47'24.4"N | 127°33'55.8"E |
| W2 | Udo Island | 0 | 2015-03-19 | 33°31'32.88"N | 126°57'10.022"E |
| W4 | Udo Island | 0 | 2015-03-19 | 33°29'52.663"N | 126°58'18.584"E |
| DJ3 | Jeju Island Soesokkak | 0 | 2015-03-20 | 33°19'7.864"N | 126°50'40.682"E |
| Ar1 | Beaufort Sea 420m | 420 | 2014-03-18 | 70°47′34″N | 135°34′04″W |
| Ar2 | Beaufort Sea 410m | 410 | 2014-03-18 | 70°42′34″N | 135°34′04″W |
| Ar3 | Beaufort Sea 277m | 277 | 2014-03-18 | 70°38′96″N | 135°56′75″W |
| Ar4 | Beaufort Sea 848m | 848 | 2014-03-18 | 70°51′40″N | 136°12′67″W |
| Ar6 | Beaufort Sea 1534m | 1534 | 2014-03-18 | 70°39′35″N | 139°02′20″W |
| Ar7 | Beaufort Sea 246m | 236 | 2014-03-18 | 70°05′62″N | 138°21′21″W |
| B1 | Hampyeong mud flat | 0 | 2017-04-28 | 35°06'07.7"N | 126°27'44.9"E |
| C1 | Hampyeong mud flat | 0 | 2017-04-28 | 35°08'24.6"N | 126°23'25.6"E |
| D1 | Muan mud flat | 0 | 2017-04-28 | 35°01'56.9"N | 126°23'24.4"E |
| E2 | Muan mud flat | 0 | 2017-04-28 | 35°03'42.0"N | 126°18'01.9"E |
| F | Muan mud flat | 0 | 2017-04-28 | 35°06'17.2"N | 126°20'01.8"E |

(B)

| Classification | Proteins | Accession number | Annotations (NCBI, UniProt) | Source organism |
| --- | --- | --- | --- | --- |
| Indole-Hal | VirX1 | M4SKV1 | Uncharacterized protein | Cyanophage Syn10 |
|  | Xcc-b100-1333 | B0RQE8 | Tryptophan halogenase, probable | Xanthomonas campestris pv. campestris (strain B100) |
|  | Xcc-b100-4156 | B0RXY9 | Putative tryptophan halogenase | Xanthomonas campestris pv. campestris (strain B100) |
|  | Xcc-b100-4345 | B0RZ29 | Putative tryptophan halogenase | Xanthomonas campestris pv. campestris (strain B100) |
|  | BrvH | B4WBL8 | Tryptophan halogenase superfamily | Brevundimonas sp. BAL3 |
|  | KrmI | N/A | N/A | marine sponge Theonella swinhoei WA |
|  | MibH | W2EQU4 | Tryptophane-5-halogenase | Microbispora sp. ATCC PTA-5024 |
| Phenolic-Hal | ChlA | A0A077JG66 | Flavin-dependent halogenase ChlA | Acytostelium subglobosum |
|  | Rdc2 | B3FWT7 | Non-heme halogenase rdc2 | Metacordyceps chlamydosporia |
|  | CndH | B9ZUJ5 | Halogenase | Chondromyces crocatus |
|  | RadH | C5H881 | Non-heme halogenase radH | Floropilus chiversii |
|  | PltM | Q4KCZ3 | Halogenase PltM | Pseudomonas fluorescens |
|  | SgcC3 | Q8GMG6 | Chlorophenol-4-monooxygenase | Streptomyces globisporus |
| Pyrrole-Hal | HrmQ | C1IHU5 | HrmQ | Streptomyces griseoflavus |
|  | MalA | L0E155 | Flavin-dependent halogenase malA | Malbranchea aurantiaca |
|  | PrnC | P95482 | Monodechloroaminopyrrolnitrin halogenase PrnC | Pseudomonas fluorescens |
|  | PltA | Q4KCZ0 | FADH2-dependent halogenase PltA | Pseudomonas fluorescens |
| 5-Trp-FDH | SpmH | A0A1W7D4T7 | Tryptophan halogenase | Streptomyces sp. SCSIO 03032 |
|  | PyrH | A4D0H5 | Tryptophan 5-halogenase | Streptomyces rugosporus |
|  | AbeH | F6LWA5 | Tryptophan 5-halogenase | uncultured bacterium AB1650 |
|  | ClaH | G3K6J6 | Halogenase | Streptomyces uncialis |
|  | XszenFHal | W1J423 | Tryptophan 5-halogenase | Xenorhabdus szentirmaii DSM 16338 |
| 6-Trp-FDH | FmoD | A0A077JCX4 | Tryptophan 6-halogenase | Streptomyces sp. Sp080513GE-23 |
|  | ThdH | A1E280 | Tryptophan 6-halogenase | Streptomyces albogriseolus |
|  | KtzR | A8CF74 | KtzR | Kutzneria sp. 744 |
|  | SttH | E9P162 | Tryptophan 6-halogenase | Streptomyces toxytricini |
|  | BorH | M9QSI0 | Tryptophan 6-halogenase | uncultured bacterium |
|  | AORI_5336 | R4SX80 | FADH2 O2-dependent halogenase I | Amycolatopsis keratiniphila |
|  | Tar14 | W5VG40 | Tryptophan halogenase | Saccharomonospora sp. CNQ490 |
|  | Th-Hal | WP_023586065.1 | tryptophan 7-halogenase | Streptomyces thermolilacinus |
| 7-Trp-FDH | KtzQ | A8CF75 | KtzQ | Kutzneria sp. 744 |
|  | PrnA | P95480 | Flavin-dependent tryptophan halogenase PrnA | Pseudomonas fluorescens |
|  | RebH | Q8KHZ8 | Flavin-dependent tryptophan halogenase RebH | Lentzea aerocolonigenes |

(C)

| Accession number | Annotations (NCBI, UniProt) | Source organism | Proposed annotation |
| --- | --- | --- | --- |
| WP_083633035.1 | tryptophan 7-halogenase | Bradyrhizobium sp. NAS96.2 | 5-Trp-FDH (B1) |
| WP_074989376.1 | tryptophan 7-halogenase | Pseudoalteromonas lipolytica | Indole-FDH (C) |
| WP_074988292.1 | tryptophan 7-halogenase | Pseudoalteromonas lipolytica | Indole-FDH (C) |
| WP_074735922.1 | tryptophan 7-halogenase | Ruegeria halocynthiae | Indole-FDH (C) |
| WP_074257951.1 | tryptophan 7-halogenase | Oceanicola litoreus | Indole-FDH (C) |
| WP_074056345.1 | tryptophan 7-halogenase | Xanthomonas gardneri | Indole-FDH (C) |
| WP_073977114.1 | tryptophan 7-halogenase | Porphyrobacter donghaensis | Indole-FDH (C) |
| WP_073319617.1 | tryptophan 7-halogenase | Aestuariibacter aggregatus | Indole-FDH (C) |
| WP_073317158.1 | tryptophan 7-halogenase | Aestuariibacter aggregatus | Indole-FDH (C) |
| WP_073316785.1 | tryptophan 7-halogenase | Aestuariibacter aggregatus | Indole-FDH (C) |
| WP_073274324.1 | tryptophan 7-halogenase | Microbulbifer donghaiensis | Indole-FDH (C) |
| WP_072558974.1 | tryptophan 7-halogenase | Sphingopyxis sp. LPB0140 | Indole-FDH (C) |
| WP_072505391.1 | tryptophan 7-halogenase | Phaeobacter sp. P97 | Indole-FDH (C) |
| WP_072384754.1 | tryptophan 7-halogenase | Novosphingobium sp. NDB2Meth1 | Indole-FDH (C) |
| WP_071959214.1 | tryptophan 7-halogenase | Alteromonas mediterranea | Indole-FDH (C) |
| WP_071951514.1 | tryptophan 7-halogenase | Alteromonas mediterranea | Indole-FDH (C) |
| WP_071939229.1 | tryptophan 7-halogenase | Shewanella sp. SACH | Indole-FDH (C) |
| WP_071817490.1 | tryptophan 7-halogenase | Alteromonas sp. V450 | Indole-FDH (C) |
| WP_071688710.1 | tryptophan 7-halogenase | Nioella sediminis | Indole-FDH (C) |
| WP_071118428.1 | tryptophan 7-halogenase | Xanthomonas alfalfae | Indole-FDH (C) |
| WP_070992342.1 | tryptophan 7-halogenase | Pseudoalteromonas byunsanensis | Indole-FDH (C) |
| WP_070959939.1 | tryptophan 7-halogenase | Hyphomonas sp. Mor2 | Indole-FDH (C) |
| WP_070959938.1 | tryptophan 7-halogenase | Hyphomonas sp. Mor2 | Indole-FDH (C) |
| WP_070693667.1 | tryptophan 7-halogenase | Xanthomonas campestris | Indole-FDH (C) |
| WP_070176958.1 | tryptophan 7-halogenase | Alteromonas lipolytica | Indole-FDH (C) |
| WP_070176908.1 | tryptophan 7-halogenase | Alteromonas lipolytica | Indole-FDH (C) |
| WP_070123441.1 | tryptophan 7-halogenase | Alteromonas confluentis | Indole-FDH (C) |
| WP_069945794.1 | tryptophan 7-halogenase | Microbulbifer sp. CCB-MM1 | Indole-FDH (C) |
| WP_069944321.1 | tryptophan 7-halogenase | Alteromonas macleodii | Indole-FDH (C) |
| WP_011470475.1 | tryptophan 7-halogenase | Saccharophagus degradans | Indole-FDH (C) |
| WP_008842873.1 | tryptophan 7-halogenase | Aliiglaciecola lipolytica | Indole-FDH (C) |
| WP_008304714.1 | tryptophan 7-halogenase | Paraglaciecola agarilytica | Indole-FDH (C) |
| WP_007987124.1 | tryptophan 7-halogenase | Paraglaciecola chathamensis | Indole-FDH (C) |
| WP_066377809.1 | tryptophan 7-halogenase | Anabaena | Indole-FDH (C) |
| WP_020913738.1 | tryptophan 7-halogenase | Shewanella piezotolerans | Indole-FDH (C) |
| WP_007638844.1 | tryptophan 7-halogenase | Paraglaciecola psychrophila | Indole-FDH (C) |
| WP_007638311.1 | tryptophan 7-halogenase | Paraglaciecola psychrophila | Indole-FDH (C) |
| WP_007621036.1 | tryptophan 7-halogenase | Paraglaciecola arctica | Indole-FDH (C) |
| WP_007375483.1 | tryptophan 7-halogenase | Pseudoalteromonas sp. Bsw20308 | Indole-FDH (C) |
| WP_007375375.1 | tryptophan 7-halogenase | Pseudoalteromonas sp. Bsw20308 | Indole-FDH (C) |
| WP_007106872.1 | tryptophan 7-halogenase | Paraglaciecola polaris | Indole-FDH (C) |
| WP_007103734.1 | tryptophan 7-halogenase | Paraglaciecola polaris | Indole-FDH (C) |
| WP_007102985.1 | tryptophan 7-halogenase | Paraglaciecola polaris | Indole-FDH (C) |
| WP_006994929.1 | tryptophan 7-halogenase | Paraglaciecola mesophila | Indole-FDH (C) |
| WP_006992568.1 | tryptophan 7-halogenase | Paraglaciecola mesophila | Indole-FDH (C) |
| WP_006990975.1 | tryptophan 7-halogenase | Paraglaciecola mesophila | Indole-FDH (C) |
| WP_006011328.1 | tryptophan 7-halogenase | Glaciecola pallidula | Indole-FDH (C) |
| ALO98531.1 | Tryptophan 5-halogenase | Streptomyces hygroscopicus subsp. limoneus | 6-Trp-FDH (B2) |
| AKQ20696.1 | tryptophan-5 halogenase | uncultured bacterium | 5-Trp-FDH (B1) |
| AJC55506.1 | tryptophan-5 halogenase | Streptomyces sp. 769 | 5-Trp-FDH (B1) |
| AHE14653.1 | tryptophan-5 halogenase | uncultured bacterium | 5-Trp-FDH (B1) |
| EQC00145.1 | tryptophan-5 halogenase | Photorhabdus temperata subsp. temperata M1021 | 5-Trp-FDH (B1) |
| EFL18155.1 | tryptophan-6 halogenase | Streptomyces sp. C | 6-Trp-FDH (A2) |
| EFK99576.1 | tryptophan-6 halogenase | Streptomyces sp. SPB78 | 6-Trp-FDH (A2) |
| AKQ20699.1 | tryptophan-6 halogenase | uncultured bacterium | 7-Trp-FDH (A1) |
| WP_073564144.1 | tryptophan-7 halogenase | Burkholderia ubonensis | 7-Trp-FDH (A1) |
| WP_071763247.1 | tryptophan-7 halogenase | Burkholderia ubonensis | 7-Trp-FDH (A1) |
| WP_071752929.1 | tryptophan-7 halogenase | Burkholderia ubonensis | 7-Trp-FDH (A1) |
| WP_071733414.1 | tryptophan-7 halogenase | Burkholderia ubonensis | 7-Trp-FDH (A1) |
| WP_085530711.1 | tryptophan-7 halogenase | Pseudomonas sp. NFIX51 | 7-Trp-FDH (A1) |
| WP_082417192.1 | tryptophan-7 halogenase | Actinobacteria bacterium OK006 | 5-Trp-FDH (B1) |
| WP_081361441.1 | tryptophan-7 halogenase | Pseudomonas chlororaphis | 7-Trp-FDH (A1) |
| WP_079142554.1 | tryptophan-7 halogenase | Streptomyces noursei | 5-Trp-FDH (B1) |
| WP_079081191.1 | tryptophan-7 halogenase | Streptomyces reticuli | 5-Trp-FDH (B1) |
| WP_079046400.1 | tryptophan-7 halogenase | Streptomyces thermoautotrophicus | 5-Trp-FDH (B1) |
| WP_078957590.1 | tryptophan-7 halogenase | Streptomyces glaucescens | 5-Trp-FDH (B1) |
| WP_078903284.1 | tryptophan-7 halogenase | Streptomyces toyocaensis | 5-Trp-FDH (B1) |
| WP_074993786.1 | tryptophan-7 halogenase | Streptomyces misionensis | 6-Trp-FDH (A2) |
| WP_074467923.1 | tryptophan-7 halogenase | Streptomyces sp. WMMB 714 | 5-Trp-FDH (B1) |
| WP_074002444.1 | tryptophan-7 halogenase | Streptomyces sp. CB02056 | 6-Trp-FDH (B2) |
| WP_073920510.1 | tryptophan-7 halogenase | Streptomyces sp. CB00455 | 6-Trp-FDH (A2) |
| WP_062768746.1 (Hal2) | tryptophan-7 halogenase | Streptomyces sp. NRRL S-1521 | 6-Trp-FDH (A2) |
| WP_060286148.1 | tryptophan-7 halogenase | Burkholderia cepacia | 7-Trp-FDH (A1) |
| WP_060174491.1 | tryptophan-7 halogenase | Burkholderia cepacia | 7-Trp-FDH (A1) |
| WP_060084749.1 | tryptophan-7 halogenase | Burkholderia cepacia | 7-Trp-FDH (A1) |
| WP_041221438.1 | tryptophan-7 halogenase | Burkholderia pseudomallei | 7-Trp-FDH (A1) |
| WP_038800614.1 | tryptophan-7 halogenase | Burkholderia pseudomallei | 7-Trp-FDH (A1) |
| WP_038800259.1 | tryptophan-7 halogenase | Burkholderia pseudomallei | 7-Trp-FDH (A1) |
| WP_038793108.1 | tryptophan-7 halogenase | Burkholderia pseudomallei | 7-Trp-FDH (A1) |
| WP_038791446.1 | tryptophan-7 halogenase | Burkholderia pseudomallei | 7-Trp-FDH (A1) |
| WP_038790924.1 | tryptophan-7 halogenase | Burkholderia pseudomallei | 7-Trp-FDH (A1) |
| WP_038790348.1 | tryptophan-7 halogenase | Burkholderia pseudomallei | 7-Trp-FDH (A1) |
| WP_038778498.1 | tryptophan-7 halogenase | Burkholderia pseudomallei | 7-Trp-FDH (A1) |
| WP_038766293.1 | tryptophan-7 halogenase | Burkholderia pseudomallei | 7-Trp-FDH (A1) |
| WP_038765329.1 | tryptophan-7 halogenase | Burkholderia pseudomallei | 7-Trp-FDH (A1) |
| WP_038750271.1 | tryptophan-7 halogenase | Burkholderia pseudomallei | 7-Trp-FDH (A1) |
| WP_038741479.1 | tryptophan-7 halogenase | Burkholderia pseudomallei | 7-Trp-FDH (A1) |
| WP_073817696.1 | tryptophan-7 halogenase | Streptomyces sp. CB02261 | 6-Trp-FDH (B2) |
| WP_073789365.1 | tryptophan-7 halogenase | Streptomyces sp. CB01580 | 6-Trp-FDH (A2) |
| WP_073776777.1 (Hal5) | tryptophan-7 halogenase | Streptomyces sp. TSRI0445 | 6-Trp-FDH (B2) |
| WP_073760485.1 | tryptophan-7 halogenase | Streptomyces sp. CB02923 | 5-Trp-FDH (B1) |
| WP_073755302.1 | tryptophan-7 halogenase | Streptomyces sp. CB03234 | 5-Trp-FDH (B1) |
| WP_073480550.1 | tryptophan-7 halogenase | Streptoalloteichus hindustanus | Indole-FDH (C) |
| WP_073450046.1 | tryptophan-7 halogenase | Streptomyces yunnanensis | 5-Trp-FDH (B1) |
| WP_073439179.1 (Hal1) | tryptophan-7 halogenase | Serratia plymuthica | 7-Trp-FDH (A1) |
| WP_072436881.1 | MULTISPECIES: tryptophan-7 halogenase | Burkholderia | 7-Trp-FDH (A1) |
| WP_072161934.1 | tryptophan-7 halogenase | Photorhabdus heterorhabditis | 5-Trp-FDH (B1) |
| WP_071961801.1 | tryptophan-7 halogenase | Streptomyces cinnamoneus | 6-Trp-FDH (B2) |
| WP_071812808.1 | tryptophan-7 halogenase | Lentzea guizhouensis | 5-Trp-FDH (B1) |
| WP_071811660.1 | tryptophan-7 halogenase | Nocardia seriolae | 5-Trp-FDH (B1) |
| WP_071803089.1 | tryptophan-7 halogenase | Couchioplanes caeruleus | 5-Trp-FDH (B1) |
| WP_070021923.1 | tryptophan-7 halogenase | Streptomyces sp. F-1 | 6-Trp-FDH (A2) |
| WP_069926446.1 (Hal7) | tryptophan-7 halogenase | Streptomyces agglomeratus | 7-Trp-FDH (A1) |
| WP_069768138.1 (Hal6) | tryptophan-7 halogenase | Streptomyces sp. LUP30 | 5-Trp-FDH (B1) |
| WP_026723863.1 | tryptophan-7 halogenase | Fischerella sp. PCC 9431 | 5-Trp-FDH (B1) |
| WP_024430541.1 | tryptophan-7 halogenase | Burkholderia pseudomallei | 7-Trp-FDH (A1) |
| WP_050043114.1 | tryptophan-7 halogenase | Burkholderia pseudomallei | 7-Trp-FDH (A1) |
| WP_003983578.1 | MULTISPECIES: tryptophan-7 halogenase | Streptomyces | 5-Trp-FDH (B1) |
| AMR44308.1 (Hal3) | tryptophan 6-halogenase | Streptomyces sp. FXJ1.172 | 6-Trp-FDH (A2) |

(D)

| Accession number | Annotation (NCBI) | Source organism | BGC type | BGC length (bp) | # of genes in BGC |
| --- | --- | --- | --- | --- | --- |
| WP_006080935.1 | tryptophan 7-halogenase | Shewanella baltica BA175 | otherks-PUFA | 56,378 | 49 |
| WP_006085195.1 | tryptophan 7-halogenase | Shewanella baltica OS195 | PUFA-otherks | 56,297 | 46 |
|  | tryptophan 7-halogenase | Shewanella baltica OS678 | PUFA-otherks | 56,297 | 46 |
| WP_006327996.1 | tryptophan 7-halogenase | Serratia plymuthica S13 | nrps-t1pks-PUFA-otherks | 89,909 | 58 |
| WP_009948842.1 | tryptophan 7-halogenase | Saccharopolyspora erythraea NRRL 2338 | terpene | 39,895 | 33 |
| WP_011061881.1 | tryptophan 7-halogenase | Pseudomonas fluorescens CHA0 | other | 41,086 | 35 |
|  | tryptophan 7-halogenase | Pseudomonas fluorescens Pf 5 | other | 41,086 | 36 |
| WP_011846317.1 | tryptophan 7-halogenase | Shewanella baltica OS117 | PUFA-otherks | 56,321 | 46 |
|  | tryptophan 7-halogenase | Shewanella baltica OS155 | PUFA-otherks | 56,321 | 46 |
| WP_012088699.1 | tryptophan 7-halogenase | Shewanella baltica OS185 | PUFA-otherks | 56,375 | 48 |
| WP_012395255.1 | tryptophan 7-halogenase | Mycobacterium marinum M | nrps | 49,171 | 31 |
| WP_012588159.1 | tryptophan 7-halogenase | Shewanella baltica OS223 | otherks-PUFA | 56,336 | 45 |
| WP_013814513.1 | tryptophan 7-halogenase | Serratia AS12 | nrps-t1pks-PUFA-otherks | 89,971 | 58 |
|  | tryptophan 7-halogenase | Serratia AS13 | nrps-t1pks-PUFA-otherks | 89,938 | 58 |
|  | tryptophan 7-halogenase | Serratia plymuthica AS9 | nrps-t1pks-PUFA-otherks | 89,971 | 58 |
| WP_015037410.1 (Hal4) | tryptophan 7-halogenase | Streptomyces venezuelae ATCC 10712 | nrps-ladderane | 94,093 | 87 |
| WP_015100660.1 | tryptophan 7-halogenase | Saccharothrix espanaensis DSM 44229 | nrps | 88,271 | 48 |
| WP_015137080.1 | tryptophan 7-halogenase | Nostoc PCC 7524 | bacteriocin | 30,281 | 22 |
| WP_015348860.1 | tryptophan 7-halogenase | Myxococcus stipitatus DSM 14675 | arylpolyene | 46,605 | 43 |
| WP_015348866.1 | tryptophan 7-halogenase | Myxococcus stipitatus DSM 14675 | arylpolyene | 46,605 | 43 |
| WP_015672691.1 | tryptophan 7-halogenase | Serratia marcescens FGI94 | other | 41,086 | 46 |
| WP_015817251.1 | tryptophan 7-halogenase | Teredinibacter turnerae T7901 | nrps | 112,482 | 41 |
| WP_015818892.1 | tryptophan 7-halogenase | Teredinibacter turnerae T7901 | nrps | 112,482 | 41 |
| WP_020941990.1 | tryptophan 7-halogenase | Streptomyces collinus Tu 365 | ladderane-nrps | 58,662 | 45 |
| WP_085960657.1 | hypothetical protein | Cylindrospermum stagnale PCC 7417 | thiopeptide | 35,022 | 24 |

(E)

| Accession number | Annotations (NCBI, UniProt) | Source organism | Proposed annotation |
| --- | --- | --- | --- |
| YP_008918758.1 | tryptophan halogenase | Alteromonas macleodii str. 'Aegean Sea MED64' | Indole-FDH (C) |
| YP_006747911.1 | tryptophan halogenase | Alteromonas macleodii ATCC 27126 | Indole-FDH (C) |
| YP_006802889.1 | tryptophan halogenase | Alteromonas macleodii str. 'Balearic Sea AD45' | Indole-FDH (C) |
| YP_006824823.1 | tryptophan halogenase | Alteromonas macleodii str. 'Black Sea 11' | Indole-FDH (C) |
| YP_004427040.1 | tryptophan halogenase | Alteromonas macleodii str. 'Deep ecotype' | Indole-FDH (C) |
| YP_006798963.1 | tryptophan halogenase | Alteromonas macleodii str. 'English Channel 673' | Indole-FDH (C) |
| YP_004466854.1 | putative tryptophan halogenase | Alteromonas sp. SN2 | Indole-FDH (C) |
| YP_004087707.1 | tryptophan halogenase | Asticcacaulis excentricus CB 48 | Indole-FDH (C) |
| YP_004088382.1 | tryptophan halogenase | Asticcacaulis excentricus CB 48 | Indole-FDH (C) |
| YP_004089166.1 | tryptophan halogenase superfamily protein | Asticcacaulis excentricus CB 48 | Indole-FDH (C) |
| YP_004089398.1 | tryptophan halogenase | Asticcacaulis excentricus CB 48 | Indole-FDH (C) |
| YP_366399.1 | tryptophan halogenase | Burkholderia lata | 7-Trp-FDH (A1) |
| YP_776610.1 | tryptophan halogenase | Burkholderia ambifaria AMMD | 7-Trp-FDH (A1) |
| YP_001811924.1 | tryptophan halogenase | Burkholderia ambifaria MC40-6 | 7-Trp-FDH (A1) |
| YP_001774579.1 | tryptophan halogenase | Burkholderia cenocepacia MC0-3 | 7-Trp-FDH (A1) |
| YP_001076763.1 | tryptophan halogenase PrnA | Burkholderia pseudomallei 1106a | 7-Trp-FDH (A1) |
| YP_336273.1 | tryptophan halogenase | Burkholderia pseudomallei 1710b | 7-Trp-FDH (A1) |
| YP_001063877.1 | tryptophan halogenase PrnA | Burkholderia pseudomallei 668 | 7-Trp-FDH (A1) |
| YP_006659544.1 | tryptophan halogenase PrnA | Burkholderia pseudomallei BPC006 | 7-Trp-FDH (A1) |
| YP_008329186.1 | tryptophan halogenase | Burkholderia pseudomallei MSHR305 | 7-Trp-FDH (A1) |
| YP_008741204.1 | prnB | Burkholderia pseudomallei NCTC 13179 | 7-Trp-FDH (A1) |
| NP_420563.1 | tryptophan halogenase | Caulobacter crescentus CB15 | Indole-FDH (C) |
| NP_421601.1 | tryptophan halogenase | Caulobacter crescentus CB15 | Indole-FDH (C) |
| NP_421603.1 | tryptophan halogenase | Caulobacter crescentus CB15 | Indole-FDH (C) |
| NP_421604.1 | tryptophan halogenase | Caulobacter crescentus CB15 | Indole-FDH (C) |
| YP_001681931.1 | tryptophan halogenase | Caulobacter sp. K31 | Indole-FDH (C) |
| YP_001681932.1 | tryptophan halogenase | Caulobacter sp. K31 | Indole-FDH (C) |
| YP_001682918.1 | tryptophan halogenase | Caulobacter sp. K31 | Indole-FDH (C) |
| YP_001683466.1 | tryptophan halogenase | Caulobacter sp. K31 | Indole-FDH (C) |
| YP_001683467.1 | tryptophan halogenase | Caulobacter sp. K31 | Indole-FDH (C) |
| YP_001683469.1 | tryptophan halogenase | Caulobacter sp. K31 | Indole-FDH (C) |
| YP_003592671.1 | tryptophan halogenase | Caulobacter segnis ATCC 21756 | Indole-FDH (C) |
| YP_003593136.1 | tryptophan halogenase | Caulobacter segnis ATCC 21756 | Indole-FDH (C) |
| YP_003593137.1 | tryptophan halogenase | Caulobacter segnis ATCC 21756 | Indole-FDH (C) |
| YP_003593138.1 | tryptophan halogenase | Caulobacter segnis ATCC 21756 | Indole-FDH (C) |
| YP_001980742.1 | tryptophan halogenase | Cellvibrio japonicus Ueda107 | Indole-FDH (C) |
| YP_267772.1 | tryptophan halogenase | Colwellia psychrerythraea 34H | Indole-FDH (C) |
| YP_270368.1 | tryptophan halogenase | Colwellia psychrerythraea 34H | Indole-FDH (C) |
| YP_270401.1 | tryptophan halogenase | Colwellia psychrerythraea 34H | Indole-FDH (C) |
| YP_002371582.1 | tryptophan halogenase | Cyanothece sp. PCC 8801 | 7-Trp-FDH (A1) |
| YP_003137148.1 | tryptophan halogenase | Cyanothece sp. PCC 8802 | 7-Trp-FDH (A1) |
| YP_003914201.1 | tryptophan halogenase | Ferrimonas balearica DSM 9799 | Indole-FDH (C) |
| YP_004434518.1 | tryptophan halogenase | Glaciecola sp. 4H-3-7+YE-5 | Indole-FDH (C) |
| YP_004435927.1 | tryptophan halogenase | Glaciecola sp. 4H-3-7+YE-5 | Indole-FDH (C) |
| YP_004436445.1 | tryptophan halogenase | Glaciecola sp. 4H-3-7+YE-5 | Indole-FDH (C) |
| YP_003060836.1 | tryptophan halogenase | Hirschia baltica ATCC 49814 | Indole-FDH (C) |
| YP_003060837.1 | tryptophan halogenase | Hirschia baltica ATCC 49814 | Indole-FDH (C) |
| YP_003061166.1 | tryptophan halogenase | Hirschia baltica ATCC 49814 | Indole-FDH (C) |
| YP_003146291.1 | tryptophan halogenase | Kangiella koreensis DSM 16069 | Indole-FDH (C) |
| YP_757947.1 | tryptophan halogenase | Maricaulis maris MCS10 | Indole-FDH (C) |
| YP_757948.1 | tryptophan halogenase | Maricaulis maris MCS10 | Indole-FDH (C) |
| YP_757958.1 | tryptophan halogenase | Maricaulis maris MCS10 | Indole-FDH (C) |
| YP_497156.1 | tryptophan halogenase | Novosphingobium aromaticivorans DSM 12444 | Indole-FDH (C) |
| YP_006574045.1 | tryptophan halogenase | Phaeobacter inhibens DSM 17395 | Indole-FDH (C) |
| YP_008974789.1 | Tryptophan halogenase | Phaeobacter gallaeciensis DSM 26640 | Indole-FDH (C) |
| YP_006563688.1 | tryptophan halogenase | Phaeobacter gallaeciensis 2.10 | Indole-FDH (C) |
| YP_663371.1 | tryptophan halogenase | Pseudoalteromonas atlantica T6c | Indole-FDH (C) |
| YP_663842.1 | tryptophan halogenase | Pseudoalteromonas atlantica T6c | Indole-FDH (C) |
| YP_004068464.1 | hypothetical protein PSM_A1379 | Pseudoalteromonas sp. SM9913 | Indole-FDH (C) |
| YP_526570.1 | tryptophan halogenase | Saccharophagus degradans 2-40 | Indole-FDH (C) |
| YP_526875.1 | tryptophan halogenase | Saccharophagus degradans 2-40 | Indole-FDH (C) |
| YP_007037755.1 | Tryptophan halogenase | Saccharothrix espanaensis DSM 44229 | 6-Trp-FDH (B2) |
| YP_008140156.1 | hypothetical protein y4xG | Serratia plymuthica 4Rx13 | 7-Trp-FDH (A1) |
| YP_006021697.1 | tryptophan halogenase | Shewanella baltica BA175 | Indole-FDH (C) |
| YP_006036484.1 | tryptophan halogenase | Shewanella baltica OS117 | Indole-FDH (C) |
| YP_001365353.1 | tryptophan halogenase | Shewanella baltica OS185 | Indole-FDH (C) |
| YP_001365391.1 | tryptophan halogenase | Shewanella baltica OS185 | Indole-FDH (C) |
| YP_001553644.1 | tryptophan halogenase | Shewanella baltica OS195 | Indole-FDH (C) |
| YP_002359089.1 | tryptophan halogenase | Shewanella baltica OS223 | Indole-FDH (C) |
| YP_002359125.1 | tryptophan halogenase | Shewanella baltica OS223 | Indole-FDH (C) |
| YP_750000.1 | tryptophan halogenase | Shewanella frigidimarina NCIMB 400 | Indole-FDH (C) |
| YP_750474.1 | tryptophan halogenase | Shewanella frigidimarina NCIMB 400 | Indole-FDH (C) |
| YP_002310992.1 | Tryptophan halogenase | Shewanella piezotolerans WP3 | Indole-FDH (C) |
| YP_003556267.1 | tryptophan halogenase | Shewanella violacea DSS12 | Indole-FDH (C) |
| YP_001759528.1 | tryptophan halogenase | Shewanella woodyi ATCC 51908 | Indole-FDH (C) |
| YP_006917054.1 | tryptophan halogenase | Simiduia agarivorans SA1 = DSM 21679 | Indole-FDH (C) |
| YP_006918525.1 | tryptophan halogenase | Simiduia agarivorans SA1 = DSM 21679 | Indole-FDH (C) |
| YP_615793.1 | tryptophan halogenase | Sphingopyxis alaskensis RB2256 | Indole-FDH (C) |
| YP_006249765.1 | tryptophan 5-halogenase | Streptomyces hygroscopicus subsp. jinggangensis 5008 | 6-Trp-FDH (B2) |
| YP_003072158.1 | tryptophan halogenase PrnA | Teredinibacter turnerae T7901 | Indole-FDH (C) |
| YP_004853680.1 | tryptophan halogenase | Xanthomonas axonopodis pv. citrumelo F1 | Indole-FDH (C) |
| YP_005639438.1 | tryptophan halogenase | Xanthomonas campestris pv. raphani 756C | Indole-FDH (C) |
| YP_366108.1 | tryptophan halogenase | Xanthomonas campestris pv. vesicatoria str. 85-10 | Indole-FDH (C) |

(F)

| Sequence name | Accession number of  homologous protein | Percent  identity (%) | Annotation (NCBI) | Source organism | Proposed annotation |
| --- | --- | --- | --- | --- | --- |
| Ar1_1222680_69_1637_- | NDF85594.1 | 68.67 | tryptophan 7-halogenase | Gammaproteobacteria bacterium | Indole-FDH (C) |
| Ar1_1273459_1548_3064_+ | PCJ50188.1 | 77.09 | tryptophan halogenase | Gammaproteobacteria bacterium | Indole-FDH (C) |
| Ar1_1374816_225_1709_- | NNE61453.1 | 75.26 | tryptophan 7-halogenase | Woeseia sp. | Indole-FDH (C) |
| Ar1_2283313_359_1873_+ | NDF85594.1 | 68.27 | tryptophan 7-halogenase | Gammaproteobacteria bacterium | Indole-FDH (C) |
| Ar1_2529987_2232_3716_+ | NNC58199.1 | 85.34 | tryptophan 7-halogenase | Woeseiaceae bacterium | Indole-FDH (C) |
| Ar1_3761472_837_2447_- | NQY97541.1 | 79.43 | tryptophan 7-halogenase | Henriciella sp. | Indole-FDH (C) |
| Ar1_6094293_1222_2841_- (MHal3) | WP_142899656.1 | 60.16 | tryptophan 7-halogenase | Denitrobaculum tricleocarpae | Indole-FDH (C) |
| Ar1_7075257_392_1882_- | NNE61453.1 | 70.04 | tryptophan 7-halogenase | Woeseia sp. | Indole-FDH (C) |
| Ar1_891060_213_1690_+ | NNC58199.1 | 87.06 | tryptophan 7-halogenase | Woeseiaceae bacterium | Indole-FDH (C) |
| Ar2_1639432_208_1692_- | NCF63038.1 | 78.83 | tryptophan halogenase | Gammaproteobacteria bacterium | Indole-FDH (C) |
| Ar2_4877749_103_1581_- | NCF20387.1 | 90.02 | tryptophan halogenase | Haliea sp. | Indole-FDH (C) |
| Ar2_6630624_173_1669_+ | NNC58199.1 | 85.54 | tryptophan 7-halogenase | Woeseiaceae bacterium | Indole-FDH (C) |
| Ar2_679057_1532_3079_+ | NEO47718.1 | 46.26 | tryptophan 7-halogenase | Moorea sp. SIO4A3 | Indole-FDH (C) |
| Ar2_871240_165_1718_+ (MHal2) | RCJ31433.1 | 43.03 | tryptophan halogenase | Nostoc punctiforme NIES-2108 | Indole-FDH (C) |
| Ar2_871512_181_1803_- (MHal1) | TDJ38333.1 | 67.23 | tryptophan 7-halogenase | Gammaproteobacteria bacterium | Indole-FDH (C) |
| Ar3_14288_361_1920_+ | QIS94332.1 | 41.14 | Tjp10 | Fischerella ambigua 108b | Indole-FDH (C) |
| Ar4_1577928_7948_9471_- (MHal4) | WP_152646774.1 | 82.7 | tryptophan 7-halogenase | Thalassomonas actiniarum | Indole-FDH (C) |
| Ar4_429396_3524_5101_- | WP_101226616.1 | 81.26 | tryptophan 7-halogenase | Colwellia sp. 75C3 | Indole-FDH (C) |
| Ar4_550878_322_1800_+ | WP_166163325.1 | 64.21 | tryptophan 7-halogenase | Pseudomaricurvus alcaniphilus | Indole-FDH (C) |
| Ar7_464686_162_1676_+ | NDF85594.1 | 68.41 | tryptophan 7-halogenase | Gammaproteobacteria bacterium | Indole-FDH (C) |
| B1_1147162_10926_12431_+ | NCF25318.1 | 90.89 | tryptophan halogenase | Gammaproteobacteria bacterium | Indole-FDH (C) |
| B1_180418_1931_3439_+ | NCF25318.1 | 91.72 | tryptophan halogenase | Gammaproteobacteria bacterium | Indole-FDH (C) |
| B1_453176_4451_5965_- | NDF85594.1 | 70.65 | tryptophan 7-halogenase | Gammaproteobacteria bacterium | Indole-FDH (C) |
| B1_531782_7174_8661_- | NNE61453.1 | 68.64 | tryptophan 7-halogenase | Woeseia sp. | Indole-FDH (C) |
| B1_758926_379_1887_- | NCF25318.1 | 92.12 | tryptophan halogenase | Gammaproteobacteria bacterium | Indole-FDH (C) |
| B1_794804_7734_9218_+ | NNC58199.1 | 85.25 | tryptophan 7-halogenase | Woeseiaceae bacterium | Indole-FDH (C) |
| B1_852963_634_2148_+ | NDF85594.1 | 70.22 | tryptophan 7-halogenase | Gammaproteobacteria bacterium | Indole-FDH (C) |
| C1_129573_654_2159_- | NCF25318.1 | 90.89 | tryptophan halogenase | Gammaproteobacteria bacterium | Indole-FDH (C) |
| D1_330689_510_2015_+ | NCF25318.1 | 90.28 | tryptophan halogenase | Gammaproteobacteria bacterium | Indole-FDH (C) |
| D1_976237_539_2047_- | NOR19735.1 | 83.81 | tryptophan halogenase | Xanthomonadales bacterium | Indole-FDH (C) |
| DJ3_890664_225_1775_- | NJM73989.1 | 41 | tryptophan 7-halogenase | Scytonema sp. RU_4_4 | Indole-FDH (C) |
| E2_124021_5936_7633_+ | WP_167228033.1 | 80.46 | tryptophan 7-halogenase | Pelagibius litoralis | Indole-FDH (C) |
| E2_1268410_131_1630_+ | KPJ79598.1 | 83.05 | tryptophan halogenase | Gammaproteobacteria bacterium SG8_30 | Indole-FDH (C) |
| E2_228729_317_1807_- | NNL51934.1 | 78.05 | tryptophan 7-halogenase | Woeseiaceae bacterium | Indole-FDH (C) |
| E2_301066_6789_8279_+ | NNL51934.1 | 78.05 | tryptophan 7-halogenase | Woeseiaceae bacterium | Indole-FDH (C) |
| E2_416528_1554_3068_- | RLA31701.1 | 61.59 | tryptophan halogenase | Gammaproteobacteria bacterium | Indole-FDH (C) |
| E2_416528_3071_4669_- | NIL93470.1 | 69.58 | tryptophan 7-halogenase | Woeseiaceae bacterium | Indole-FDH (C) |
| E2_435280_3233_4747_- | NDF85594.1 | 70.45 | tryptophan 7-halogenase | Gammaproteobacteria bacterium | Indole-FDH (C) |
| E2_482376_10089_11573_+ | NNE61453.1 | 73.52 | tryptophan 7-halogenase | Woeseia sp. | Indole-FDH (C) |
| E2_585216_4941_6497_+ | MBJ6640112.1 | 38.39 | tryptophan 7-halogenase | Streptomyces sp. DHE7-1 | Indole-FDH (C) |
| F_1114305_569_2068_+ | NCF25318.1 | 92.43 | tryptophan halogenase | Gammaproteobacteria bacterium | Indole-FDH (C) |
| F_251358_14711_16204_- | NNL46711.1 | 80.85 | tryptophan 7-halogenase | Woeseiaceae bacterium | Indole-FDH (C) |
| F_2554_5839_7323_- | NNC58199.1 | 85.86 | tryptophan 7-halogenase | Woeseiaceae bacterium | Indole-FDH (C) |
| F_473795_148_1653_- | NCF25318.1 | 91.09 | tryptophan halogenase | Gammaproteobacteria bacterium | Indole-FDH (C) |
| F_566696_267_1766_+ | WP_049630884.1 | 58.06 | tryptophan 7-halogenase | Cellvibrio sp. pealriver | Indole-FDH (C) |
| F_753061_34_1527_+ | KPJ79598.1 | 85.32 | tryptophan halogenase | Gammaproteobacteria bacterium SG8_30 | Indole-FDH (C) |
| F_928665_1795_3279_- | NNC58199.1 | 85.25 | tryptophan 7-halogenase | Woeseiaceae bacterium | Indole-FDH (C) |
| NH1_3098306_502_1974_+ | NNL46711.1 | 85.69 | tryptophan 7-halogenase | Woeseiaceae bacterium | Indole-FDH (C) |
| NH1_3672489_588_2072_+ | NIM69896.1 | 75.66 | tryptophan halogenase | Xanthomonadales bacterium | Indole-FDH (C) |
| NH2_199207_5380_6867_+ | WP_116808401.1 | 71.75 | tryptophan 7-halogenase | Steroidobacter cummioxidans | Indole-FDH (C) |
| NH2_3216005_68_1561_- | NNL51934.1 | 81.58 | tryptophan 7-halogenase | Woeseiaceae bacterium | Indole-FDH (C) |
| NH2_401775_7204_8718_- | NQY97535.1 | 56.51 | tryptophan 7-halogenase | Henriciella sp. | Indole-FDH (C) |
| NH2_483900_3915_5420_+ | NCF25318.1 | 91.3 | tryptophan halogenase | Gammaproteobacteria bacterium | Indole-FDH (C) |
| NH2_524061_5392_6906_- | NQY40856.1 | 58.12 | tryptophan 7-halogenase | Henriciella sp. | Indole-FDH (C) |
| NH2_774148_2434_3963_- | NQY40856.1 | 56.31 | tryptophan 7-halogenase | Henriciella sp. | Indole-FDH (C) |
| NH3_1269263_10303_11787_+ | NIM69896.1 | 75.51 | tryptophan halogenase | Xanthomonadales bacterium | Indole-FDH (C) |
| NH3_2469726_2595_4133_- | TDG15197.1 | 66.53 | tryptophan 7-halogenase | Halieaceae bacterium GH4-78 | Indole-FDH (C) |
| NH3_2641698_729_2237_- | NNL03666.1 | 77.46 | tryptophan 7-halogenase | Xanthomonadales bacterium | Indole-FDH (C) |
| NH3_3942581_717_2297_+ | WP_033078320.1 | 64.74 | tryptophan 7-halogenase | Thalassotalea sp. ND16A | Indole-FDH (C) |
| NH3_4374000_39_1553_+ | NDF85594.1 | 69.42 | tryptophan 7-halogenase | Gammaproteobacteria bacterium | Indole-FDH (C) |
| NH3_4392738_1657_3141_+ | WP_142930230.1 | 77.05 | tryptophan 7-halogenase | Exilibacterium tricleocarpae | Indole-FDH (C) |
| NH3_4680628_250_1737_- | RPH95316.1 | 82.76 | tryptophan 7-halogenase | Xanthomonadales bacterium | Indole-FDH (C) |
| NH3_4687895_214_1755_+ | RLA31701.1 | 67.13 | tryptophan halogenase | Gammaproteobacteria bacterium | Indole-FDH (C) |
| NH3_568524_201_1703_+ | NNJ78373.1 | 79.44 | tryptophan 7-halogenase | Xanthomonadales bacterium | Indole-FDH (C) |
| NH3_743163_7164_8651_- | NNJ64833.1 | 86.59 | tryptophan 7-halogenase | Xanthomonadales bacterium | Indole-FDH (C) |
| W4_185593_883_2367_+ | NNC58199.1 | 85.07 | tryptophan 7-halogenase | Woeseiaceae bacterium | Indole-FDH (C) |
| W4_2246360_834_2345_+ | WP_049630884.1 | 57.32 | tryptophan 7-halogenase | Cellvibrio sp. pealriver | Indole-FDH (C) |
| W4_2524533_987_2501_+ | NQY97535.1 | 57.11 | tryptophan 7-halogenase | Henriciella sp. | Indole-FDH (C) |
