# Supplementary material for: Genomic Determinants Encode the Reactivity and Regioselectivity of Flavin-Dependent Halogenases in Bacterial Genomes and Metagenomes
Source: mSystems. 2021 May 27;6(3):e00053-21. doi: 10.1128/mSystems.00053-21 (PMC8269204; doi:10.1128/mSystems.00053-21)
Supplement: TABLE S2 [file msystems.00053-21-st002.docx]

**Table S2.** The gene sequences and primer sequences used for site-directed mutagenesis of the selected putative FDHs (Hal1–7 and MHal1–4). The homologous proteins of MHals were identified using BLASTp against a non-redundant protein database. NA: not applicable

|  | Accession number | Source  organism | Amino acid sequence | BGC type | Mutations (**motifs**) | Primers |
| --- | --- | --- | --- | --- | --- | --- |
| Hal1 | WP_073439179.1 | *Serratia*  *plymuthica* | MSKPIKNIVIVGGGTAGWMSASYLVRALQQQANITLIESA AIPRIGVGEATIPSLQKVFFDFLGIPEQEWMPQVNGAFKA GIKFVNWRKSPDHSRNDYFYHLFGNVPSCDGVPLTHYWLR KREQGFQQSMAYACYPQPGALDGNLAPCLHDGTRQMSHAW HFDAHLVADFLQRWAVDRGVNRVVDEVMDVNLNDHGFISS LITKEGRKLEADLFIDCSGMRGLLINQALKEPFIDMSDYL LCDSAVASAVPNDDAQVGVEPYTSAIAMNSGWTWKIPMLG RFGSGYVFSSKFTSRDQATTDFLNLWGLSDNQPLNQIKFR VGRNKRAWVNNCVSIGLSSCFLEPLESTGIYFIYAALYQL VKHFPDTSFDPRLADAFNAEITYMFDDCRDFVQAHYFTSS REDTPFWLANRHDLRLSDSIKEKVERYKAGLPLTTTSFDD STYYETFDFEFKNFWLNGNYYCIFAGLGMLPDRSLPLLQL RPESIEKAEAMFARIQREAERLRASLPTNYDYLRSLREGD AGLFRPGPTAASPESL | NA | 443-YY to AA (**Trp4**) | 5'-GAC GAT AGT ACC GCG GCG GAA ACC TTT G-3' |
|  |  |  |  |  |  | 5'-CAA AGG TTT CCG CCG CGG TAC TAT CGT C-3' |
|  |  |  |  |  | E450A (**Trp4**) | 5'-CCT TTG ATT TCG CGT TCA AGA ACT TTT GG-3' |
|  |  |  |  |  |  | 5'-CCA AAA GTT CTT GAA CGC GAA ATC AAA GG-3' |
|  |  |  |  |  | N459A (**Trp4**) | 5'-GGC TGA ATG GTG CGT ATT ACT GTA TCT TTG-3' |
|  |  |  |  |  |  | 5'-CAA AGA TAC AGT AAT ACG CAC CAT TCA GCC-3' |
| Hal2 | WP_062768746.1 | *Streptomyces* sp. NRRL S-1521 | MDQRVKEIVILGGGTAGWMSAAYLAKKLGETVRVTVLEAP SIPRIGVGEATVPNLQRVFFDQLGLSEEEWMPECNAAYKV AVKFINWCTPGKGEARAREIDGRPDHFHHPFGLLPEHEGL PLSHYWVYNRKHGVTGKPFDYACFPGVAIMDALKAPRDHA GEPVTRYAWHFDAHLVADFLRRHATEKQNVTHVQDEMVEA RRDGRGFVTALRTKSGAVIEGDLFIDCSGFRGLLINQAME EPFVDMSDHLLCDRAVAYALPNDDEANGVEPYTSAIAMES GWTWKIPMLGRFGTGYVYSDAFADQDRATTDFCRMWGLDP DEVGDRLNHVRFRVGRNRRAWVKNVVSVGLASCFLEPLES TGIYFITAALHHLAKHFPDQRFDAVLADRFNAEIEAMFDD TRDFIQAHFALSPRTDTPFWKANKDLHLPEGIKEKVAMYR AGLPINQPASTEGAYYDNFDVEFRNFWTNGSYYCVFAGLG LEPDHPLPALMHHPDAAEGAQHLFDRVAEEQRKLADTLPS NHEYLRRLHGA | NA | V52I (**Trp1**) | 5’-AAG CAA CCA TCC CGA ATC TGC AGC G-3’ |
|  |  |  |  |  |  | 5’-CGC TGC AGA TTC GGG ATG GTT GCT T-3’ |
|  |  |  |  |  | P110L (**Trp2**) | 5'-GGA TCA TTT TCA TCA TCT GTT TGG TCT GCT GC-3' |
|  |  |  |  |  |  | 5'-GCA GCA GAC CAA ACA GAT GAT GAA AAT GAT CC-3' |
|  |  |  |  |  | S471N (**Trp4**) | 5'-CTT CTG GAC CAA TGG TAA CTA TTA TTG TGT G-3' |
|  |  |  |  |  |  | 5'-CAC ACA ATA ATA GTT ACC ATT GGT CCA GAA G-3' |
| Hal3 | AMR44308.1 | *Streptomyces* sp. FXJ1.172 | MSTRTDSHLDTHNDTRNDTRIQKVVILGGGTAGWMTAAYL GKALQNTVQITVLEAPSIPRIGVGEATIPNLQRAFFDYLG IAEEEWMRECNASYKMAVKFINWRTPGEGSPSPRTVDGHP DTFHHPFGLLPSADHIPLSHYWAAKTLRGETTEPYDHACF VDTAIMDARKAPRWLDSRRATNYAWHFDAQLVADFLRTFA VTKQAVEHVEDEMTEALTDERGYLTALRTKSGRLLDGDLF IDCSGFRGLLINQAMGEPFLDMSDHLLCDSAVATAVPHDD ETHGVEPYTSSIAMPAGWTWKIPTLGRFGSGYVFSSRFAD RDQATAEFARLWGLDPEQTPFNHIRFRVGRNRRAWVRNCV GIGLASCFVEPLESSGIYFVYAAIHMLAKHFPDRTFDPVL VDRFNREIQDMFDDTRDFLQAHYYFSPRTDTPFWRANKEL ELADGIKDKAETYRAGLPVNLPISDEGTYYGNFEAEFRNF WTNGSYYCIFAGLGMLPRHPLPSLAHKPESVARAEQLFAD IKRKQHEWVRSLPSTHQLLRHLHGAS | Thienodolin | NA | NA |
| Hal4 | WP_015037410.1 | *Streptomyces venezuelae* | MLNRVVIVGGGTAGWMTASYFKAAFGERIDITLVESGSVG AVGVGEATFSDIRHFFEFLGLKEKDWMPACNATYKLAVRF ENWRQPGHYFYHPFEQMRSVNGFPLTDWWLKNGPTDRFDK DCFVMASVIDAGLSPRHRDGTLIDQAFDEDGNEMQGLTMS EHQGKTQFPYAYQFEAALLAKYLTRYSVERGVKHIVDDVT KVELDERGWISAVKTAEHGDITGDVYIDCTGFRGVLINQA LEEPFISYQDTLPNDSAVALQVPMDMERRGIRPCTTATAQ DAGWIWTIPLTGRVGTGYVYAKDYLSPEDAERTLREFVGP AAADVEANHIKMRIGRTRNSWVKNCVAIGLSSGFVEPLES TGIFFIHHAIEQLAKNFPGEDWNPAHRDLYNNAIAHVMDG VREFLVLHYVAAKRSDTQYWRDTKTRAIPDSLAERIEKWK VQLPDSESIFPYYHGLPAYSYMCILLGMGGIDLAPSPALA LSDPSAALQEFELIREKTERLVKVLPKAYDYFTQLG | nrps-ladderane | 47-ATFSDI to ATFSTV (Trp1) | 5'-GCA ACC TTT AGC ACC GTG CGT CAT TTC TTT G-3' |
|  |  |  |  |  |  | 5'-CAA AGA AAT GAC GCA CGG TGC TAA AGG TTG C-3' |
|  |  |  |  |  | Q96R (**Trp2**) | 5'-CAT CCG TTT GAA CGT ATG CGC AGC G-3' |
|  |  |  |  |  |  | 5'-CGC TGC GCA TAC GTT CAA ACG GAT G-3' |
|  |  |  |  |  | 456-LPA to FES (**Trp4**) | 5'-GTA TTA TCA TGG CTT CGA AAG CTA TAG TTA TAT G-3' |
|  |  |  |  |  |  | 5'-CAT ATA ACT ATA GCT TTC GAA GCC ATG ATA ATA C-3' |
| Hal5 | WP_073776777.1 | *Streptomyces* sp. TSRI0445 | MTVDGAGKQRAGDPGRTKRVVVVGGGTAGWMTASYLAAAF GERIDVTVVESARVGTIGVGEATFSDIRHFFEFLKLSEPD WMPACNATYKLAVRFENWREPGHHFYHPFEQLGSVDGFPL SDWWLRNPTTSRFDKDCFVMASLCDAERSPRYLDGKLIDQ GFVEQQREETSARSTIVEYQGTQFPYAYHFEAALLAKFLT TYATQRGVRHITDDVTDVVLDQDGYIAQVQTAEHGRLEGD LFVDCTGFRALLLNKALDEPFVSYQDTLPNDSAVALQVPL DMDREPMRPCTTATAQEAGWIWTIPLISRIGSGYVYASDY TTPEEAEHTLRAFIGPGAEDVEANHIKMRIGRSRRSWVKN CVGIGLSSGFVEPLESTGIFFIHHAIEQIVKYFPSGREDH RLRDLYNESIAHVQDGVREFLVLHYVGAKRADNQYWKDTK TRRVPDELAERIENWKYKVPDAQTVFPHYHGLPPYSYNCI LLGTGGIEVRHSPALDMADERAAIAEFERIRLKADKLVQE LPTQNEYFAAMRAGAV | NA | NA | NA |
| Hal6 | WP_069768138.1 | *Streptomyces* sp. LUP30 | MIDSVVIVGGGTAGWMTASYLKAAFDDRLKVTLVESERVT RIGVGEATFSTVRHFFDYLGLDESEWLPKCAGGYKLGIRF ENWRGPGQHFYHPFERLRSADGFSLADWWLRIGDRSRPFD RQCFITTALCEAKRSPRLMDGSLFSMDLDGSLGRSTLEEQ RAQFPYAYHFDADLVAKFLAEYSTDRGVRHVIDDVVEVGQ DERGWLSHVTTKEHGRIEGDLFIDCTGFRGLLINKTLGET FESFNDVLPNNRAVALRVPREHATEMNPYTTATAMDAGWI WTIPLFRRNGNGYVYSDEYITPEEAEQTLRDFVGPGHEDL EANHIRMRIGRNDRSWVKNCVAIGLASAFVEPLESTGIFF IQHGIEQLVKHFPDEHWDPQLAADYNSRVAHVVDGVKEFL VLHYAAATREDTPYWKEAKVRAMPEGLKERLASARSHLLD EESIYPYYHGFESYSWNAMLMGLGLEPAGPRPALAHLDPA GAEREFARLKEEADQMVAALPSCYQYLASLHA | NA | 47-ATFSTV to ATFSDI (**Trp1**) | 5'-GCC ACC TTT AGT GAT ATT CGT CAT TTC TTT G-3' |
|  |  |  |  |  |  | 5'-CAA AGA AAT GAC GAA TAT CAC TAA AGG TGG C-3' |
|  |  |  |  |  | R96Q (**Trp2**) | 5'-CAT CCG TTT GAA CAG CTG CGT AGT GCA G-3' |
|  |  |  |  |  |  | 5'-CTG CAC TAC GCA GCT GTT CAA ACG GAT G-3' |
|  |  |  |  |  | Q160A (**Trp3**) | 5’-CCC TGG AAG AAG CGC GTG CAC AGT TTC CG-3’ |
|  |  |  |  |  |  | 5’-CGG AAA CTG TGC ACG CGC TTC TTC CAG GG-3’ |
|  |  |  |  |  | Q163A (**Trp3**) | 5’-GAA GCG CGT GCA GCG TTT CCG TAT GCA TAT C-3’ |
|  |  |  |  |  |  | 5’-GAT ATG CAT ACG GAA ACG CTG CAC GCG CTT C-3’ |
|  |  |  |  |  | 160-QRAQ to QGKTQ (Trp3) | 5'-GGA AGA ACA GGG CAA AAC CCA GTT TCC G-3' |
|  |  |  |  |  |  | 5'-CGG AAA CTG GGT TTT GCC CTG TTC TTC C-3' |
|  |  |  |  |  | 451-FES to LPP (**Trp4**) | 5'-CCG TAT TAT CAT GGT CTG CCG CCG TAT AGT TGG-3' |
|  |  |  |  |  |  | 5'-CCA ACT ATA CGG CGG CAG ACC ATG ATA ATA CGG-3' |
|  |  |  |  |  | 451-FES to LPA (**Trp4**) | 5'-CCG TAT TAT CAT GGT CTG CCG GCG TAT AGT TGG-3' |
|  |  |  |  |  |  | 5'-CCA ACT ATA CGC CGG CAG ACC ATG ATA ATA CGG-3' |
| Hal7 | WP_069926446.1 | *Streptomyces agglomeratus* | MSDNLIKKILVLGGGTAGWMTASYLGKALGSTVRITVLEA PAIPKIGVGEATIPNLQRVFFDYLGLTEEEWMPECNASYK MGIRFINWRTPGTGTARPRPYGQYGDQFDHLFGLLPNHDN LPLSHYWTYQKLNGLTDEPFDRACYPQPALFDRKLSPRFP DGRRVASYAWHFDADLVADFLRRFATARQGATHIEDKFIA AETDQRGHLVAVTTESGRRLEADLFIDCSGFRSLLINQVM KEPFLDMSDHLLNDRAVATRLEHDDEEHGVEPYTSAIAMS SGWAWKIPMLGRFGTGYVYSSRFTSQEEATREFCQMWGIS PDTHPMNHVRFRVGRNRRAWVKNCVGIGLSSCFLEPLEST GIYFTYAALYQLVKHFPDKRFDPMLTKSFNSEIEAMFDDT RDFIQGHFSFAPRDDTPFWRACKELELAPEFVRKVEMYKA GLGVDLPVTDESTYYGNFEAEFRNFWSNANYYCVFAGLGM MPEHDPTPLDYRPESVKSAEAVFAQVQRRRDELLDTLPPM HTYLRRLHGK | NA | I53V (**Trp1**) | GGT GAA GCC ACC GTG CCG AAT CTG CAG |
|  |  |  |  |  |  | CTG CAG ATT CGG CAC GGT GGC TTC ACC |
|  |  |  |  |  | L111P (**Trp2)** | GAT CAG TTT GAT CAT CCG TTT GGT CTG CTG |
|  |  |  |  |  |  | CAG CAG ACC AAA CGG ATG ATC AAA CTG ATC |
|  |  |  |  |  | N470S (**Trp4**) | GG AGC AAT GCC AGC TAT TAT TGC GTT TTT G |
|  |  |  |  |  |  | CAA AAA CGC AAT AAT AGC TGG CAT TGC TCC |
|  | Accession number | Source organism of homologous protein/ Accession number of homologous protein (perc. id.) | Amino acid sequence | BGC type | Mutations | Primers |
| MHal1 | MZ043106 | Gamma-proteobacteria bacterium/ TDJ38333.1  (67.23%) | MHMTPKRVLIVGGGASGWMAAAYLNAALKDNGQRPVEISL IESPDVPQIDVSEATMPNIKQFLAVVGIDEVDFLMRVDGT FKQAIKYVDWSHGHGDYFFHSFDSYRTQPIDRAAMRWLMS DRSIPFGETTSTQPIICELGLAPKPLNGQGAVKPLSYGYH MSALKFADLLREIATARGVKHYLDDVTDVEMAENGNIAAV VTEGGKHLEADLFIDCTGSTARLIEQQLGVDWVDCSQWLL CDRALTMNVGYEQHYSGSVRPYTTATAMSSGWVSEIPLQT QKALGYVYSSAHISEDEAERELRAFEGPHADSLATRTVHF KVGHRAKAWARNCVSIGPSSGFIEPLESTDLYLSSLAAVM LAEHFPFDDDMAPLAYRYNRIMANRFYEILDFINLHYCLT QRADTEFWREVRKSERINDRLQAKLDYWKIKVPSMSDFVD QHFPGQPDTLLPTADLPGDHRAPIDTAAVFGIQNYEAILY GMGFLEEECNQWFGDKRPKLRVPKYIVDELRRAPDALPAH DVWLQQLAGMPVYPVTPGASRQ | NA | NA | NA |
| MHal2 | MZ043107 | *Nostoc punctiforme* NIES-2108/ RCJ31433.1  (42.89%) | MQGQIKNILIVGGGTAGWMTACMLSKRLDDINITLIESSD IPTVGVGEATIIQMNFFLREMGLTENDWMPSCNATYKEGI YFENFYRNGEHYWNPFQPLGVESTDYWIHKYYKENLSPDS YFDYCFSNTVRNSNNRIDLETKINQDQIRNVFYTYHLDAS LFAQHLKAKVALPGGVKHIVDNVTEVNLAEDGSVSGIETG ANGTLNADLYVDCSGFRSLLLGDKLGEPFIDYNDRLPNDR AIAIRMPYEDRDKEMHPYTSATALEAGWVWNIPLWHRIGT GYVYSSQYKSEDDAEFEFRQHLGEDRVKDLQAHHIKMRIG KYECPWKKNVVGIGLAGGFVEPLESTGIELAQLGAGFLSW YLKKSPTNRAISQKLYNAKMQSVYDEVSDYIQLHYVLTDR EDTEYWRDQKYTEADIRTSVLHKLTRRDASFSFDETGDVF SNTAWNALLIGMRQIPVHDIFSRSRPIDPNKFAMANKAME DNYRKAKQQAVAVSGGVKNPTHHEYLAKTVYKGIEIE | NA | NA | NA |
| MHal3 | MZ043108 | *Denitrobaculum tricleocarpae/* WP_142899656.1  (60.16%) | MVAMNTAQSTDQEGAHGGGAVNRIDEVTIVGGGTAGWLTA MVLNTTLNIRRSGPPVRITLIESPNVPTIGVGEATVPGMP NLLRQLRIDESAFFKATNASFKLGVRFVDWQLDDAGRPTS YIHPFNSAPAVGGYLPAYHYHRFGRPEGCEDLADAVAPNS SVIKGYFGPRPIGGKEYEGLIGYAYHLDAALFAKFLRSIA LERGVRHVVDDVVDVRLAENGFVSALHLKDGGLMPVQFVV DCTGFRGLIIQKALGEPFESYGDQLLCDSALAVQLPHQDP EHLEPCTRSTALGAGWCWRVPLYTRVGTGYVFSSRFRSDD AAMDEFMEYLGDHGKGAEPRVIRMRVGRTRNAWVKNCAAI GLSGGFIEPLESTAIYMIEMAARWLSHYFPNREVSPALAE AFNGHMRNLTEEVRDFIVTHYYTSNRPDPFWVAARSEIEL SDRLRARLELWRHTLPGSTDTAGDKLFSFWNYLYVLYCKG YFKEASFPLAGSVDQGDWQDYTRRLNDTKADLRRQLPNHY ALLTSIRERADEAAVPLRE | NA | NA | NA |
| MHal4 | MZ043109 | *Thalassomonas actiniarum/* WP_152646774.1  (82.74%) | MQTTNQSLEQTGTTKKRQRVVIAGGGSAGWMTAASLSKLL GKSLDISLIESEEIGRIGVGEATIPPLRTFHELLGINEQD FMKATQATFKLGIEFKDWGKRENSYIHSFGDTGKICWAAE FQHFWLAGLKKGIKAEFGAYCPEHAAAKAGKVLGANDSRL HYAYHLDAGLYADYLKKMSIAKGVNRIEGKIIDVGIHPET GYIDTLTLDNGTLIEGDLFIDCTGFAARLIEGALNTGFES YGHLIPCDTAVAVQTEKAGLPRPYTQAMAHDFGWQWRIPL QHRVGNGLVFCSRYTSDEDAIAALMDNLESPAITEPKIFR YKTGRRLKGWNKNCIAIGLASGFVEPVESTAIHLIMASIL RLMKLFPHEEISQANIDEYNNQTQEEMERIRDFIILHYKA TERDDTAFWRYCQNMQVPQTLAHRMALFKKSAIGSSTQSE RVLSDSWTQVMMGQGIIPENYHQIVDQMSEQELSKFLQSI KAQVDGQVQQMPSHQEFIDHYCKAPLL | NA | NA | NA |
