# Supplementary material for: Genomic Determinants Encode the Reactivity and Regioselectivity of Flavin-Dependent Halogenases in Bacterial Genomes and Metagenomes
Source: mSystems. 2021 May 27;6(3):e00053-21. doi: 10.1128/mSystems.00053-21 (PMC8269204; doi:10.1128/mSystems.00053-21)
Supplement: FIG S3 [file msystems.00053-21-sf003.pdf]

(A1)

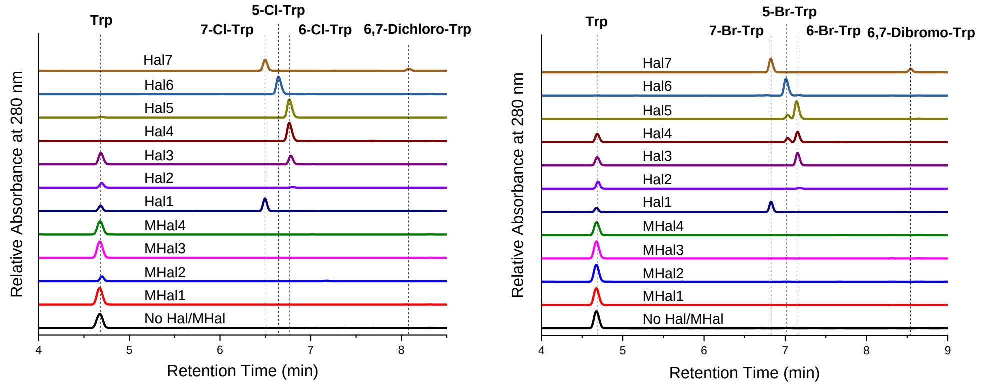
Trp
7-Cl-Trp
5-Cl-Trp
6-Cl-Trp
6,7-Dichloro-Trp
Hal7
Hal6
Hal5
Hal4
Hal3
Hal2
Hal1
MHal4
MHal3
MHal2
MHal1
No Hal/MHal
Relative Absorbance at 280 nm
Retention Time (min)
Trp
7-Br-Trp
5-Br-Trp
6-Br-Trp
6,7-Dibromo-Trp
Hal7
Hal6
Hal5
Hal4
Hal3
Hal2
Hal1
MHal4
MHal3
MHal2
MHal1
No Hal/MHal
Relative Absorbance at 280 nm
Retention Time (min)

(A2)

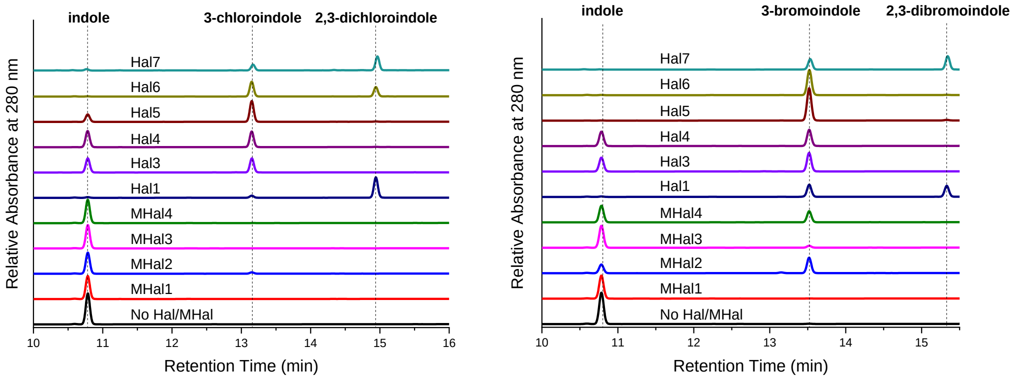
indole
3-chloroindole
2,3-dichloroindole
Hal7
Hal6
Hal5
Hal4
Hal3
Hal1
MHal4
MHal3
MHal2
MHal1
No Hal/MHal
Relative Absorbance at 280 nm
Retention Time (min)
indole
3-bromoindole
2,3-dibromoindole
Hal7
Hal6
Hal5
Hal4
Hal3
Hal1
MHal4
MHal3
MHal2
MHal1
No Hal/MHal
Relative Absorbance at 280 nm
Retention Time (min)

(A3)

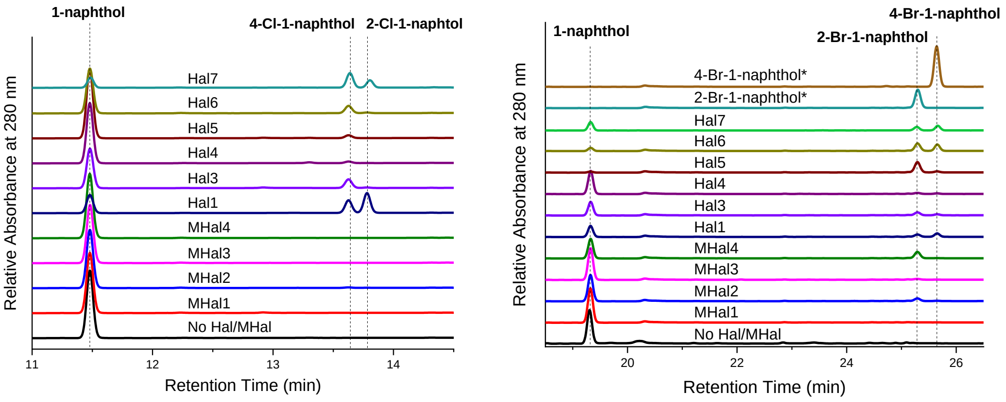
1-naphthol
4-Cl-1-naphthol
2-Cl-1-naphtol
Hal7
Hal6
Hal5
Hal4
Hal3
Hal1
MHal4
MHal3
MHal2
MHal1
No Hal/MHal
Relative Absorbance at 280 nm
Retention Time (min)
1-naphthol
2-Br-1-naphthol
4-Br-1-naphthol
4-Br-1-naphthol*
2-Br-1-naphthol*
Hal7
Hal6
Hal5
Hal4
Hal3
Hal1
MHal4
MHal3
MHal2
MHal1
No Hal/MHal
Relative Absorbance at 280 nm
Retention Time (min)

(A4)

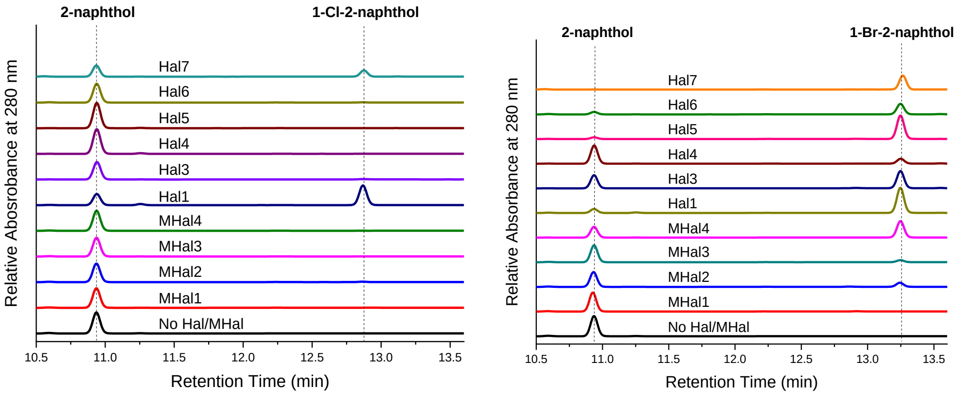
2-naphthol
1-Cl-2-naphthol
Hal7
Hal6
Hal5
Hal4
Hal3
Hal1
MHal4
MHal3
MHal2
MHal1
No Hal/MHal
Relative Absorbance at 280 nm
10.5
11.0
11.5
12.0
12.5
13.0
13.5
Retention Time (min)
2-naphthol
1-Br-2-naphthol
Hal7
Hal6
Hal5
Hal4
Hal3
Hal1
MHal4
MHal3
MHal2
MHal1
No Hal/MHal
Relative Absorbance at 280 nm
10.5
11.0
11.5
12.0
12.5
13.0
13.5
Retention Time (min)

(A5)

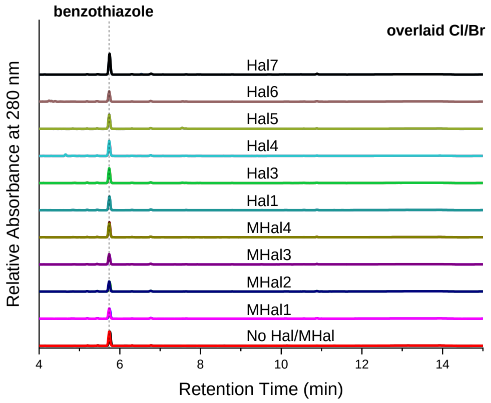
benzothiazole
overlaid Cl/Br
Hal7
Hal6
Hal5
Hal4
Hal3
Hal1
MHal4
MHal3
MHal2
MHal1
No Hal/MHal
Relative Absorbance at 280 nm
4
6
8
10
12
14
Retention Time (min)

(A6)

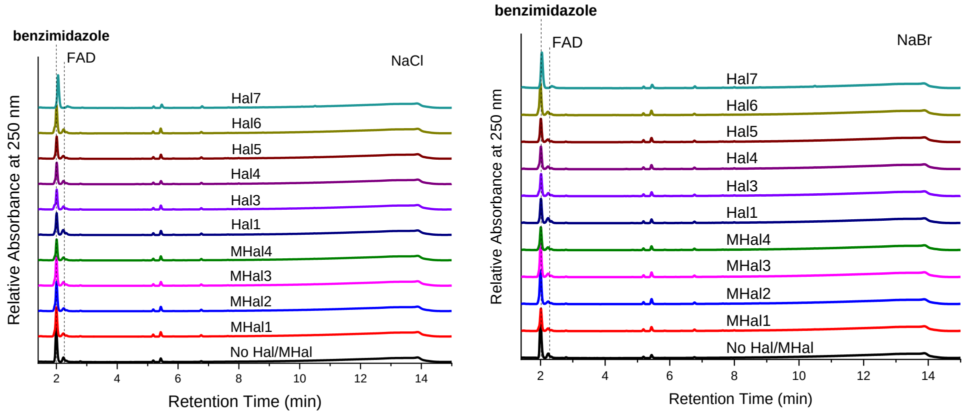
benzimidazole
FAD
NaCl
Hal7
Hal6
Hal5
Hal4
Hal3
Hal1
MHal4
MHal3
MHal2
MHal1
No Hal/MHal
Relative Absorbance at 250 nm
2
4
6
8
10
12
14
Retention Time (min)
benzimidazole
FAD
NaBr
Hal7
Hal6
Hal5
Hal4
Hal3
Hal1
MHal4
MHal3
MHal2
MHal1
No Hal/MHal
Relative Absorbance at 250 nm
2
4
6
8
10
12
14
Retention Time (min)

(A7)

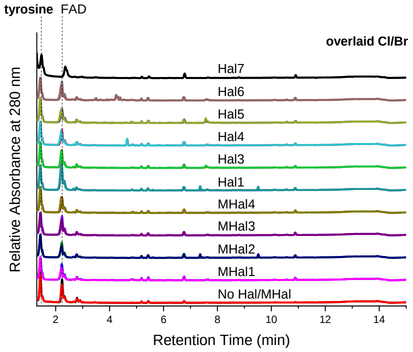
tyrosine
FAD
overlaid Cl/Br
Hal7
Hal6
Hal5
Hal4
Hal3
Hal1
MHal4
MHal3
MHal2
MHal1
No Hal/MHal
Relative Absorbance at 280 nm
Retention Time (min)

(A8)

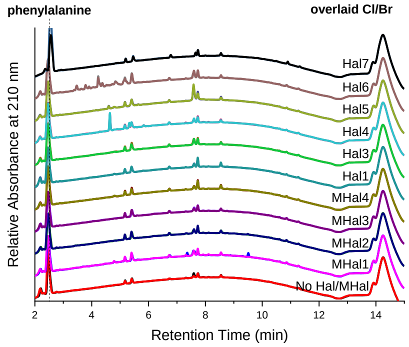
phenylalanine
overlaid Cl/Br
Hal7
Hal6
Hal5
Hal4
Hal3
Hal1
MHal4
MHal3
MHal2
MHal1
No Hal/MHal
Relative Absorbance at 210 nm
Retention Time (min)

(A9)

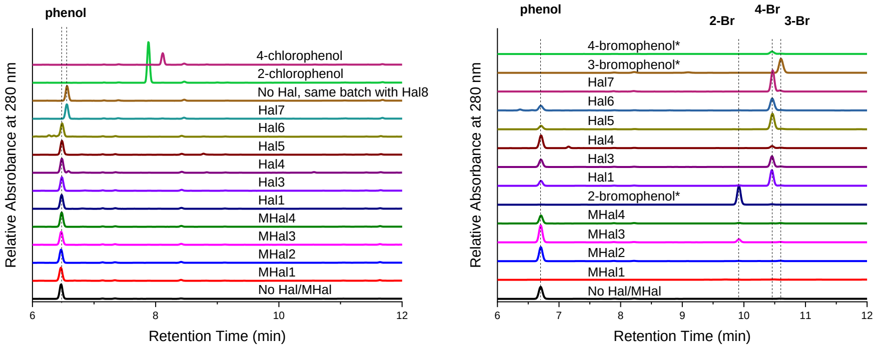
phenol
4-chlorophenol
2-chlorophenol
No Hal, same batch with Hal8
Hal7
Hal6
Hal5
Hal4
Hal3
Hal1
MHal4
MHal3
MHal2
MHal1
No Hal/MHal
Relative Absrobance at 280 nm
Retention Time (min)
phenol
2-Br
4-Br
3-Br
4-bromophenol*
3-bromophenol*
Hal7
Hal6
Hal5
Hal4
Hal3
Hal1
2-bromophenol*
MHal4
MHal3
MHal2
MHal1
No Hal/MHal
Relative Absorbance at 280 nm
Retention Time (min)

(B1)

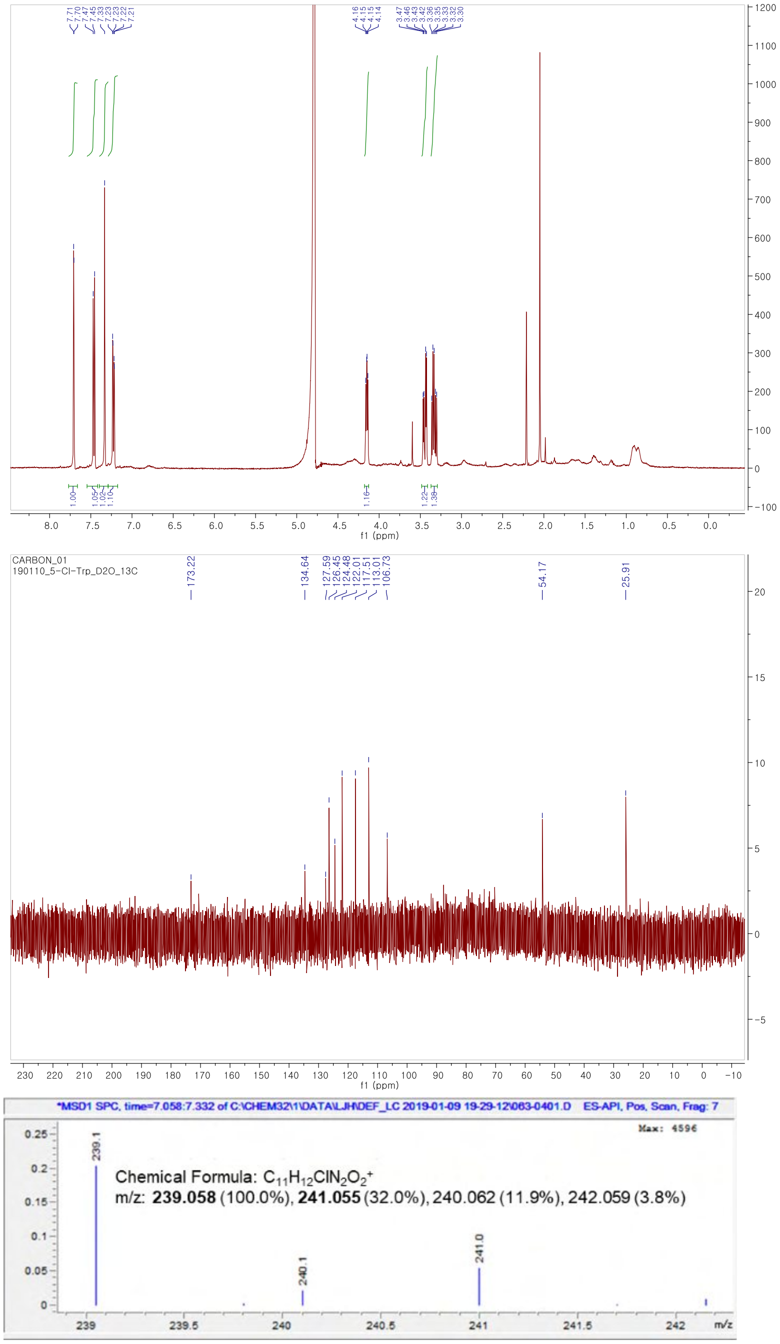
7.71
7.70
7.47
7.45
7.33
7.23
7.23
7.22
7.21
4.16
4.15
4.15
4.14
3.47
3.46
3.43
3.42
3.36
3.35
3.33
3.32
3.30
1.00
1.05
1.02
1.10
1.16
1.22
1.38
f1 (ppm)
CARBON_01
190110_5-Cl-Trp_D2O_13C
173.22
134.64
127.59
126.45
124.48
122.01
117.51
113.01
106.73
54.17
25.91
f1 (ppm)
*MSD1 SPC, time=7.058:7.332 of C:\CHEM32\1\DATA\LJH\DEF_LC 2019-01-09 19-29-12\063-0401.D ES-API, Pos, Scan, Frag: 7
Max: 4596
239.1
240.1
241.0
Chemical Formula: C11H12ClN2O2+
m/z: 239.058 (100.0%), 241.055 (32.0%), 240.062 (11.9%), 242.059 (3.8%)
m/z

(B2)

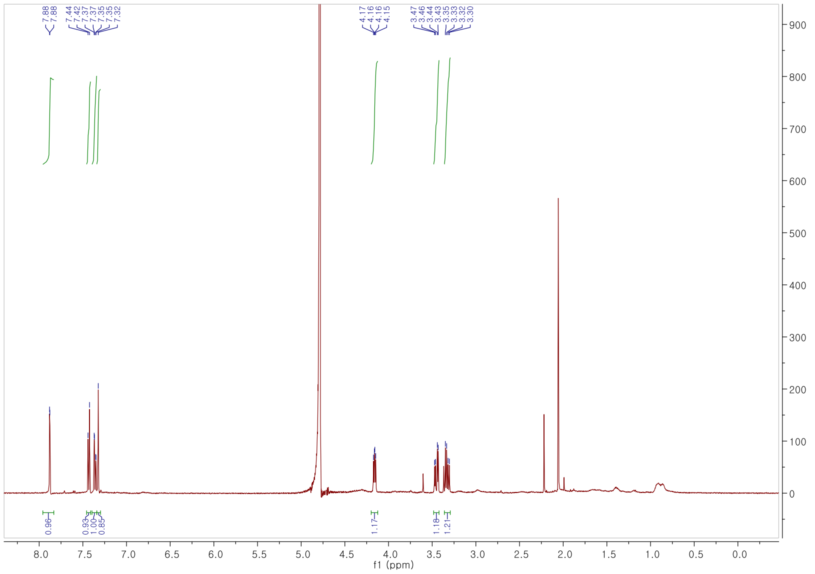
7.88
7.88
7.44
7.42
7.37
7.37
7.35
7.35
7.32
4.17
4.16
4.16
4.15
3.47
3.46
3.44
3.43
3.35
3.33
3.32
3.30
0.96
0.93
1.00
0.85
1.17
1.18
1.21
f1 (ppm)

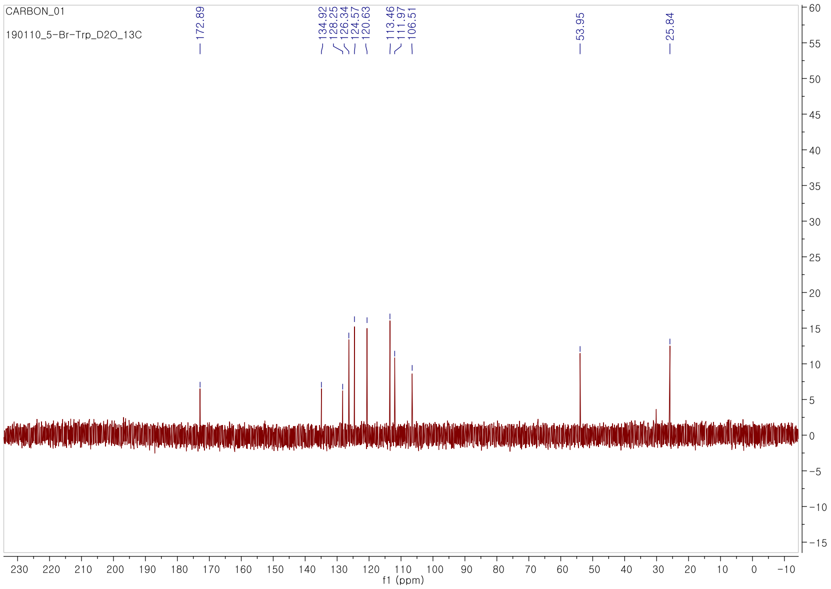
CARBON_01
190110_5-Br-Trp_D2O_13C
172.89
134.92
128.25
126.34
124.57
120.63
113.46
111.97
106.51
53.95
25.84
f1 (ppm)

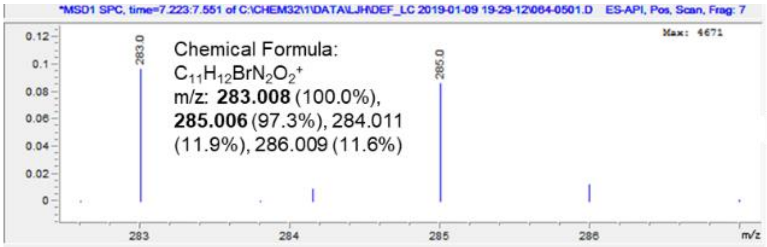
*MSD1 SPC, time=7.223:7.551 of C:\CHEM32\1\DATA\LJH\DEF_LC 2019-01-09 19-29-12\064-0501.D ES-API, Pos, Scan, Frag: 7
Max: 4671
283.0
285.0
Chemical Formula:
$C_{11}H_{12}BrN_2O_2^+$
m/z: **283.008** (100.0%),
285.006 (97.3%), 284.011
(11.9%), 286.009 (11.6%)
m/z

(B3)

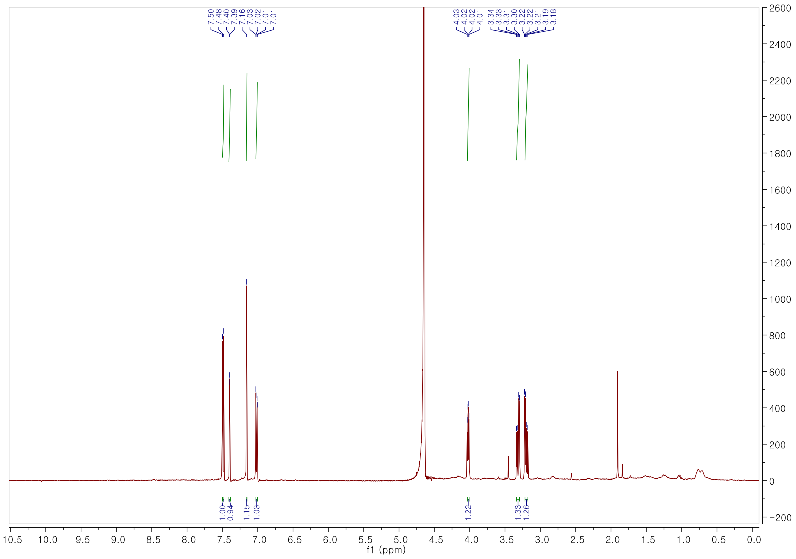

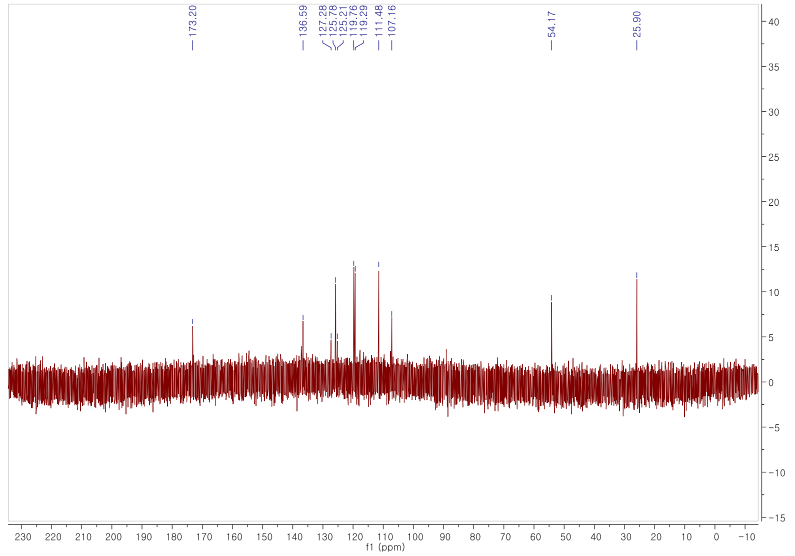

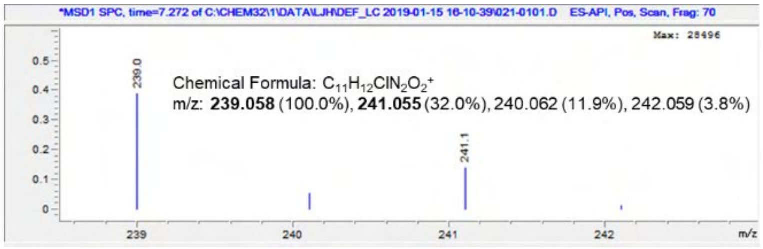
*MSD1 SPC, time=7.272 of C:\CHEM32\1\DATA\LJH\DEF_LC 2019-01-15 16-10-39\021-0101.D ES-API, Pos, Scan, Frag: 70
Max: 28496
Chemical Formula: $C_{11}H_{12}ClN_2O_2^+$
m/z: 239.058 (100.0%), 241.055 (32.0%), 240.062 (11.9%), 242.059 (3.8%)
239.0
241.1

(B4)

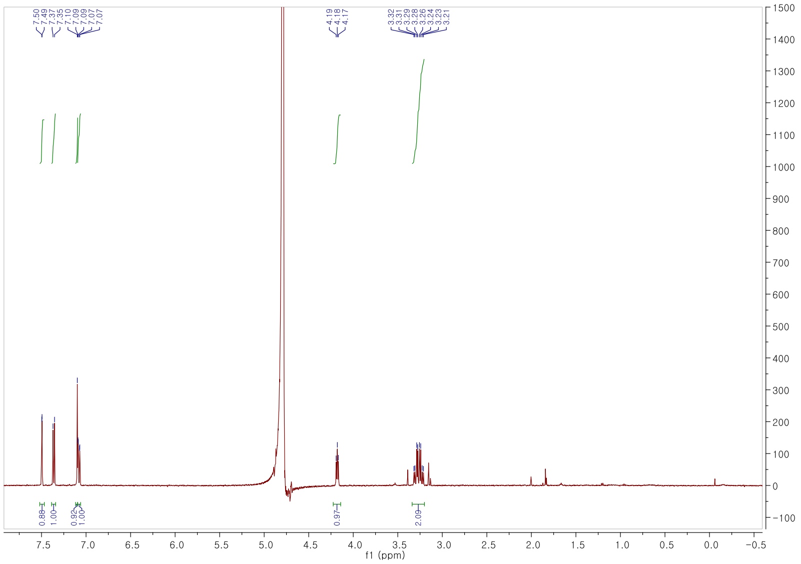
7.50
7.49
7.37
7.35
7.10
7.09
7.09
7.07
7.07
4.19
4.18
4.17
3.32
3.31
3.29
3.26
3.24
3.23
3.21
0.88
1.00
0.92
1.00
0.97
2.09
f1 (ppm)

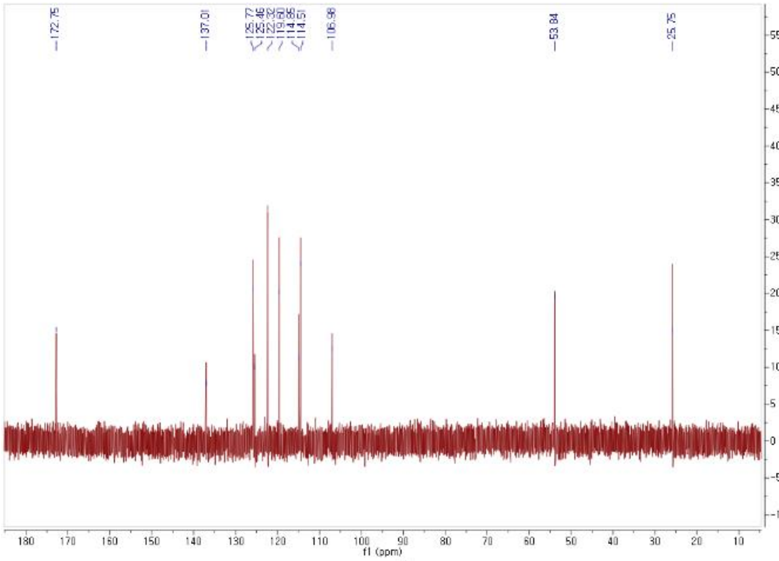
172.75
137.01
125.77
125.46
122.32
119.50
114.85
114.51
106.98
53.84
25.75
f1 (ppm)

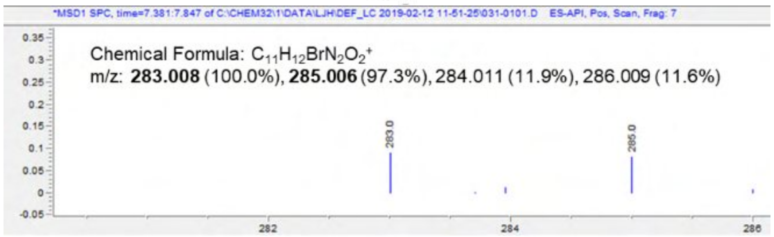
*MSD1 SPC, time=7.381:7.847 of C:\CHEM32\1\DATA\LJH\DEF_LC 2019-02-12 11-51-25\031-0101.D ES-API, Pos, Scan, Frag: 7
Chemical Formula: $C_{11}H_{12}BrN_2O_2^+$
m/z: **283.008** (100.0%), **285.006** (97.3%), 284.011 (11.9%), 286.009 (11.6%)
283.0
285.0

(B5)

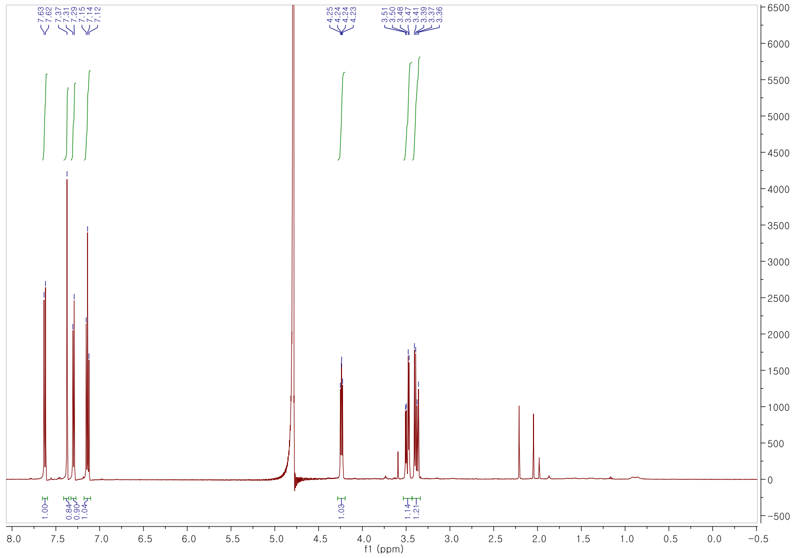

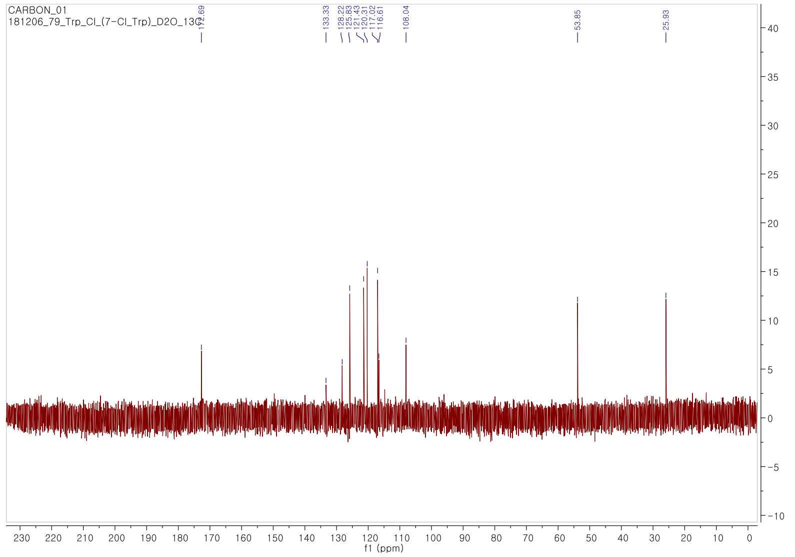
CARBON_01
181206_79_Trp_Cl_(7-Cl_Trp)_D2O_13C

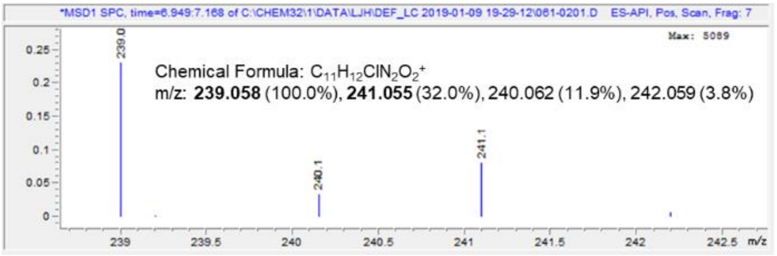
Chemical Formula: $C_{11}H_{12}ClN_2O_2^+$
m/z: **239.058** (100.0%), **241.055** (32.0%), 240.062 (11.9%), 242.059 (3.8%)

(B6)

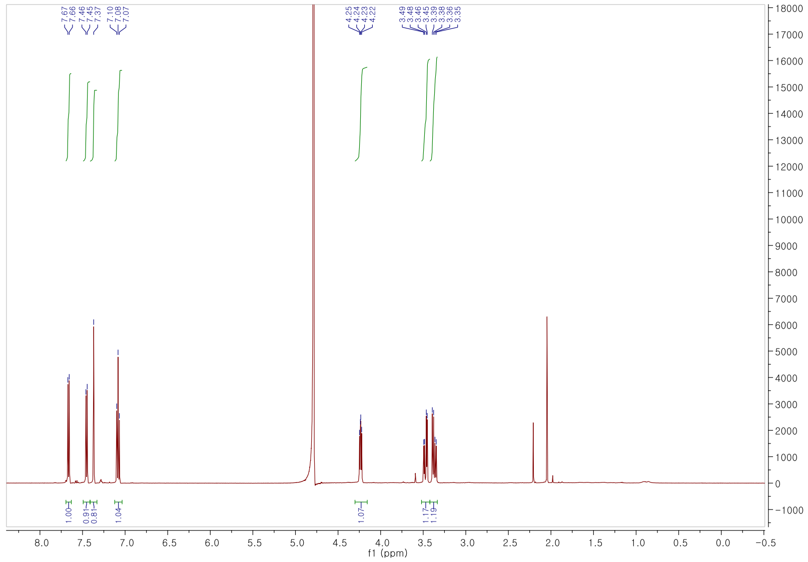

7.67
7.66
7.46
7.45
7.37
7.10
7.08
7.07
4.25
4.24
4.23
4.22
3.49
3.48
3.46
3.45
3.39
3.38
3.36
3.35
1.00
0.91
0.81
1.04
1.07
1.17
1.19
f1 (ppm)

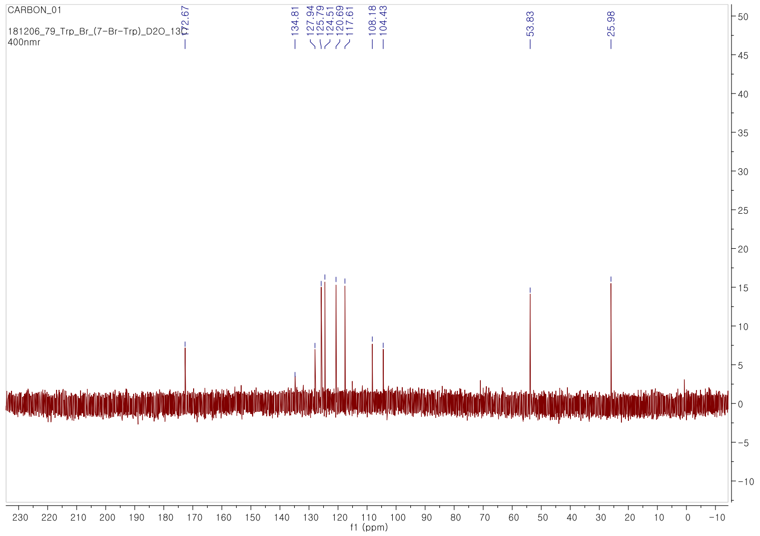

CARBON_01
181206_79_Trp_Br_(7-Br-Trp)_D2O_13C
400nmr
172.67
134.81
127.94
125.79
124.51
120.69
117.61
108.18
104.43
53.83
25.98
f1 (ppm)

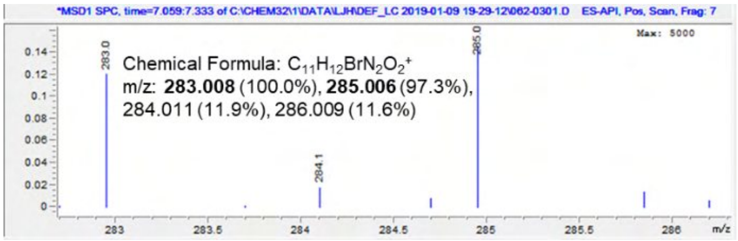

*MSD1 SPC, time=7.059:7.333 of C:\CHEM32\1\DATA\LJH\DEF_LC 2019-01-09 19-29-12\062-0301.D ES-API, Pos, Scan, Frag: 7
Max: 5000
283.0
285.0
284.1
Chemical Formula: $C_{11}H_{12}BrN_2O_2^+$
m/z: **283.008** (100.0%), **285.006** (97.3%), 284.011 (11.9%), 286.009 (11.6%)
m/z

(B7)

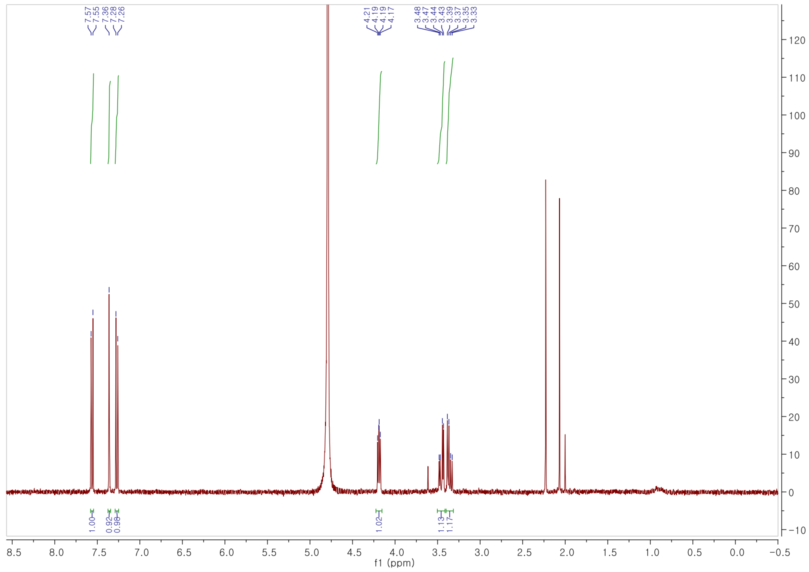
7.57
7.55
7.36
7.28
7.26
4.21
4.19
4.19
4.17
3.48
3.47
3.44
3.43
3.39
3.37
3.35
3.33
1.00
0.92
0.99
1.02
1.13
1.17
f1 (ppm)

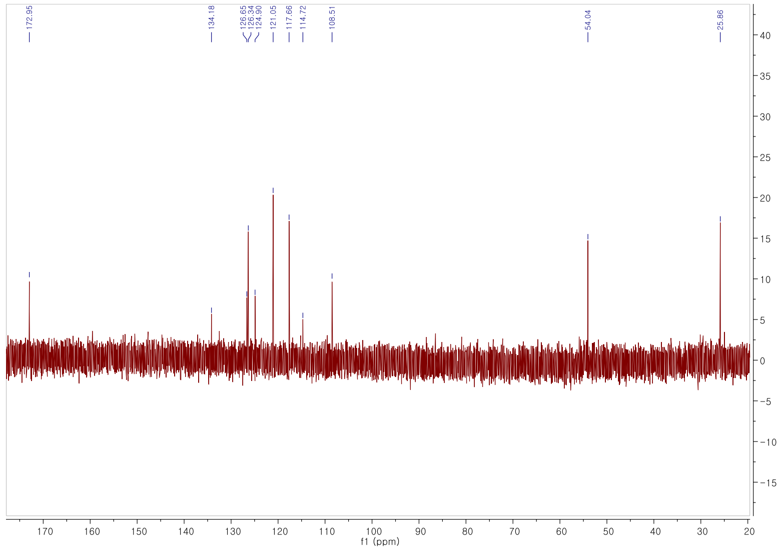
172.95
134.18
126.65
126.34
124.90
121.05
117.66
114.72
108.51
54.04
25.86
f1 (ppm)

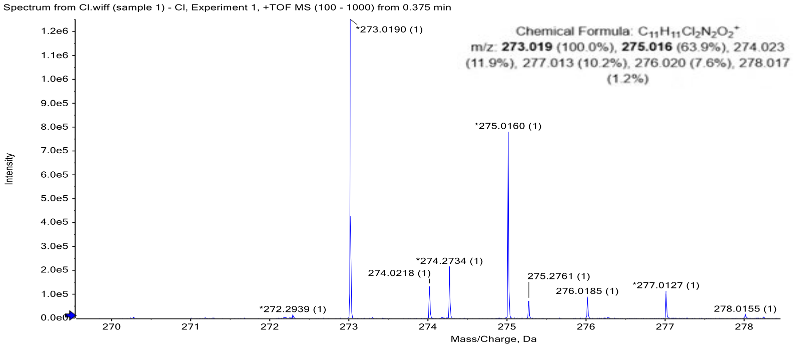
Spectrum from Cl.wiff (sample 1) - Cl, Experiment 1, +TOF MS (100 - 1000) from 0.375 min
Chemical Formula: $C_{11}H_{11}Cl_2N_2O_2^+$
m/z: **273.019** (100.0%), **275.016** (63.9%), 274.023 (11.9%), 277.013 (10.2%), 276.020 (7.6%), 278.017 (1.2%)
*273.0190 (1)
*275.0160 (1)
274.0218 (1)
*274.2734 (1)
275.2761 (1)
276.0185 (1)
*277.0127 (1)
278.0155 (1)
*272.2939 (1)
Intensity
Mass/Charge, Da

(B8)

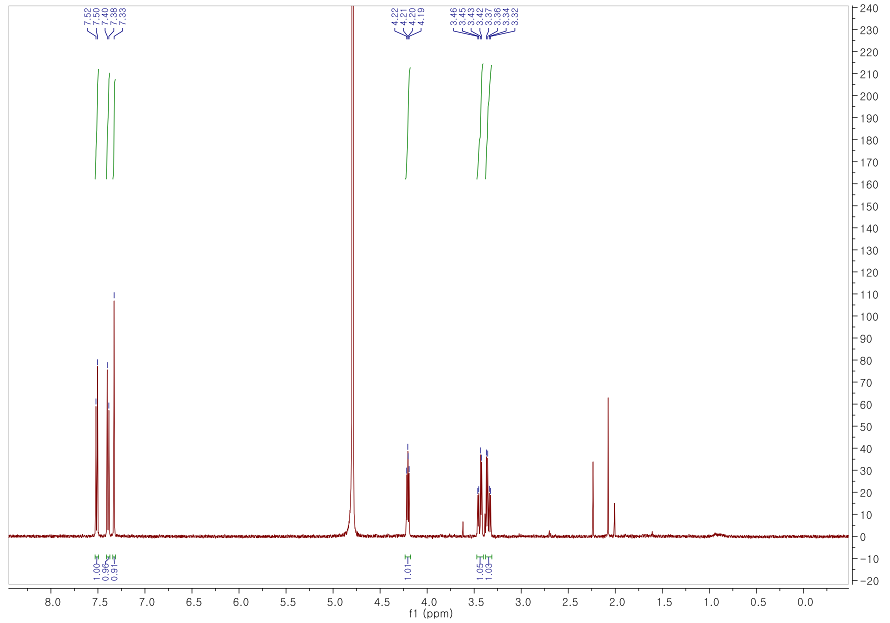

7.52
7.50
7.40
7.38
7.33
4.22
4.21
4.20
4.19
3.46
3.45
3.43
3.42
3.37
3.36
3.34
3.32
1.00
0.96
0.91
1.01
1.05
1.03
f1 (ppm)

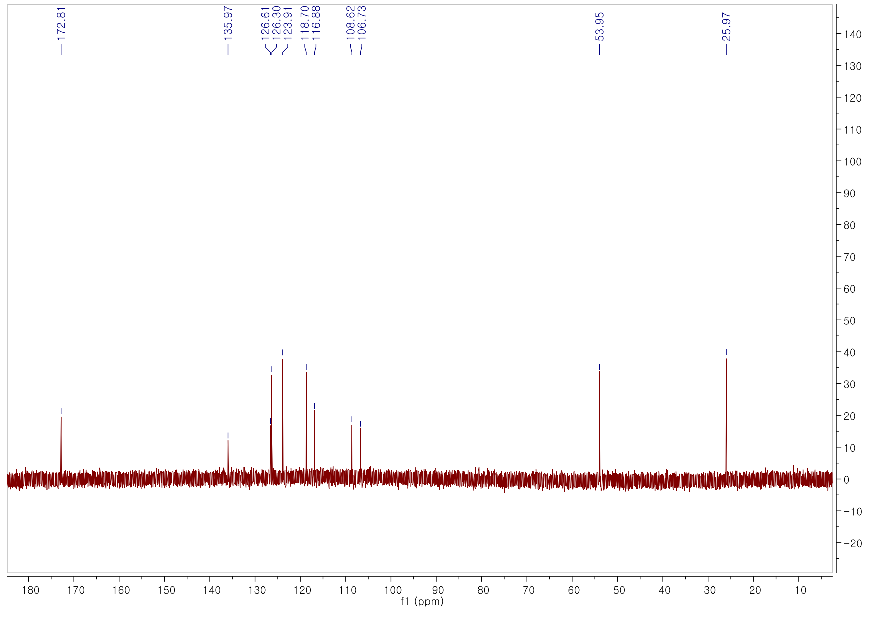

172.81
135.97
126.61
126.30
123.91
118.70
116.88
108.62
106.73
53.95
25.97
f1 (ppm)

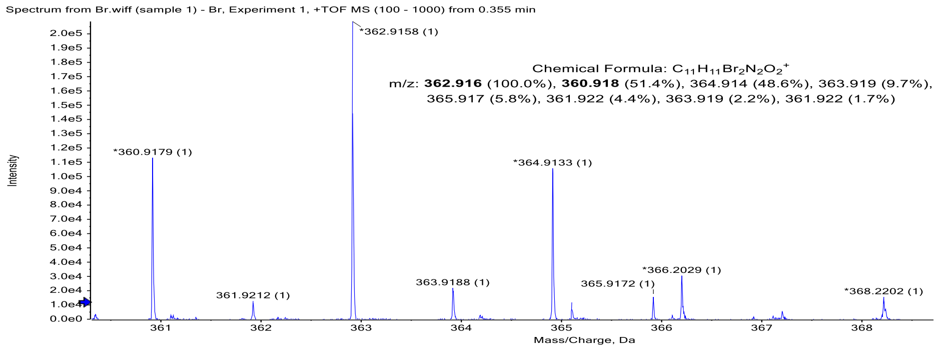
Spectrum from Br.wiff (sample 1) - Br, Experiment 1, +TOF MS (100 - 1000) from 0.355 min
Chemical Formula: $C_{11}H_{11}Br_2N_2O_2^+$
m/z: **362.916** (100.0%), **360.918** (51.4%), 364.914 (48.6%), 363.919 (9.7%), 365.917 (5.8%), 361.922 (4.4%), 363.919 (2.2%), 361.922 (1.7%)
*362.9158 (1)
*360.9179 (1)
361.9212 (1)
363.9188 (1)
*364.9133 (1)
365.9172 (1)
*366.2029 (1)
*368.2202 (1)
Intensity
Mass/Charge, Da

(B9)

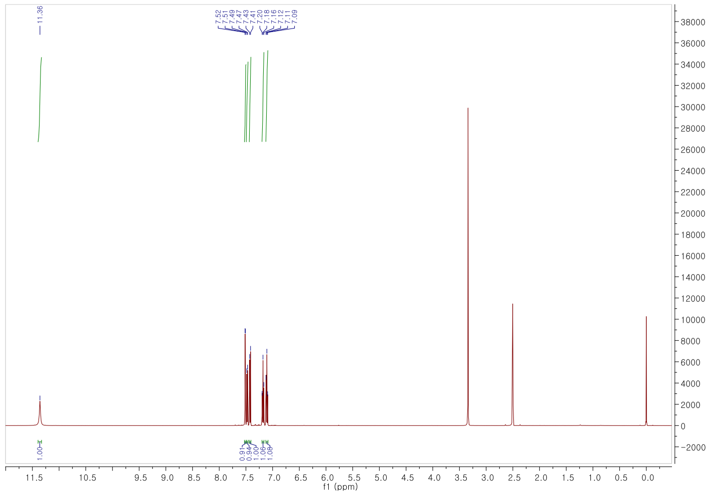
11.36
f1 (ppm)

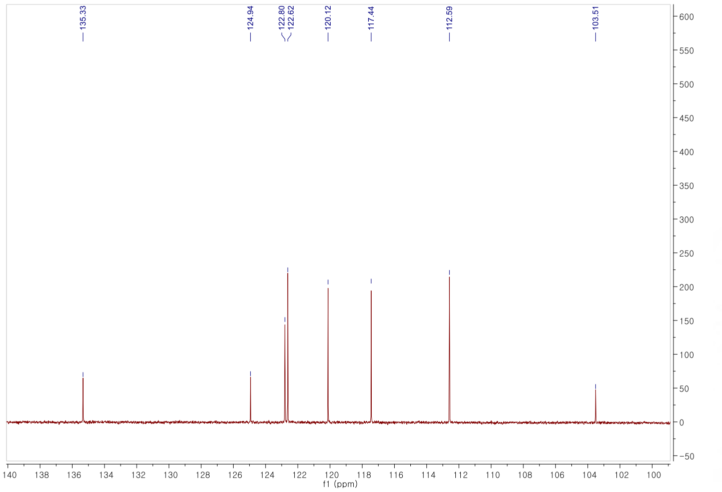
135.33
124.94
122.80
122.62
120.12
117.44
112.59
103.51
f1 (ppm)

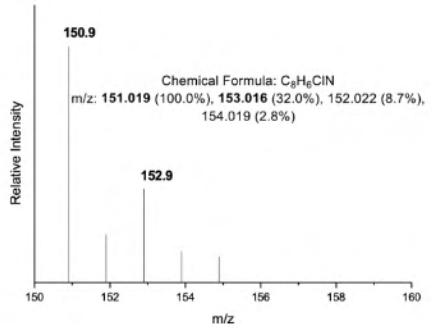
150.9
152.9
Chemical Formula: C_8H_6ClN
m/z: **151.019** (100.0%), **153.016** (32.0%), 152.022 (8.7%), 154.019 (2.8%)
Relative Intensity
m/z

(B10)

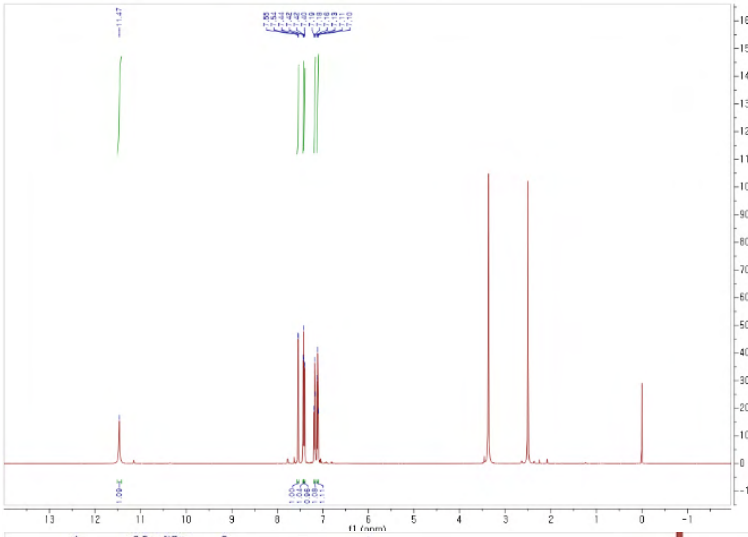

11.47
f1 (ppm)

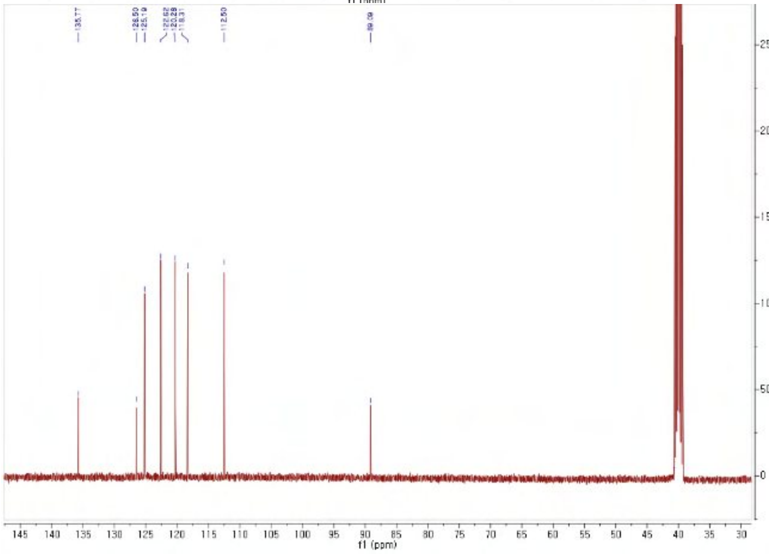

135.77
112.50
89.39
f1 (ppm)

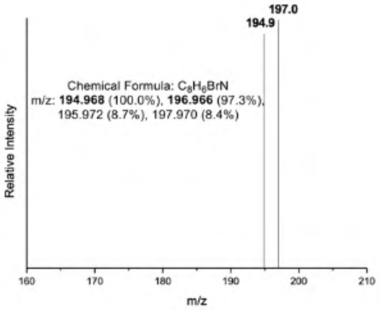

197.0
194.9
Chemical Formula: C_8H_6BrN
m/z: **194.968** (100.0%), **196.966** (97.3%),
195.972 (8.7%), 197.970 (8.4%)
Relative Intensity
m/z

(B11)

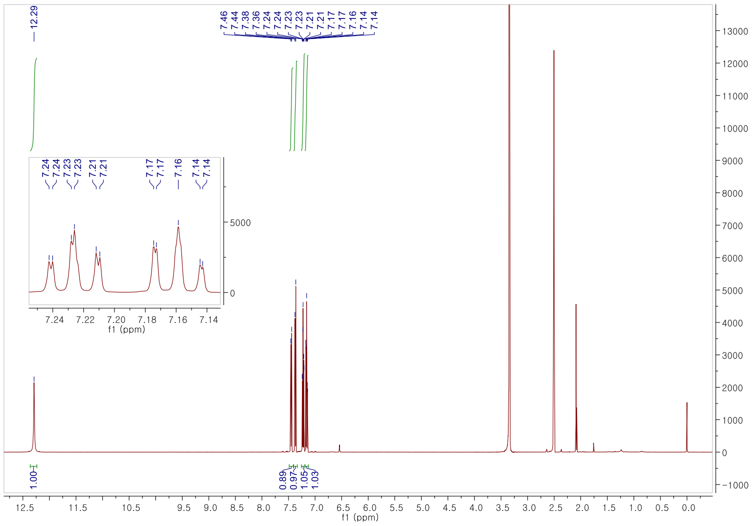

12.29
7.46 7.44 7.38 7.36 7.24 7.24 7.23 7.23 7.21 7.21 7.17 7.17 7.16 7.14 7.14
1.00
0.89 0.97 1.95 1.03
f1 (ppm)

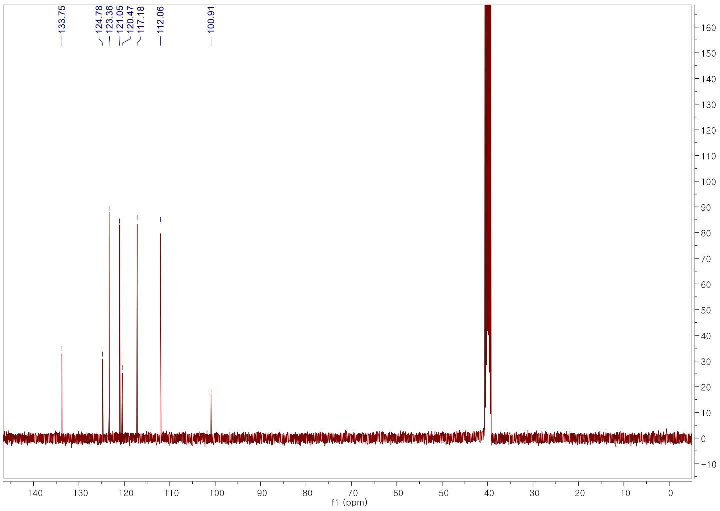

133.75
124.78 123.36 121.05 120.47 117.18
112.06
100.91
f1 (ppm)

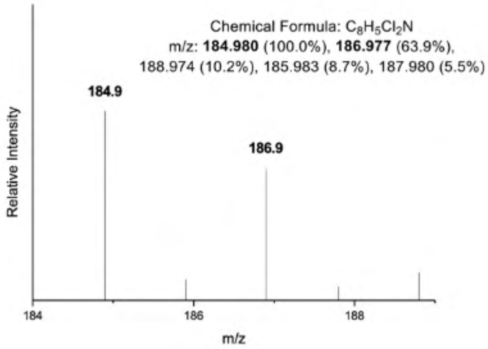

Chemical Formula: $C_8H_5Cl_2N$
m/z: **184.980** (100.0%), **186.977** (63.9%),
188.974 (10.2%), 185.983 (8.7%), 187.980 (5.5%)
184.9
186.9
Relative Intensity
m/z

(B12)

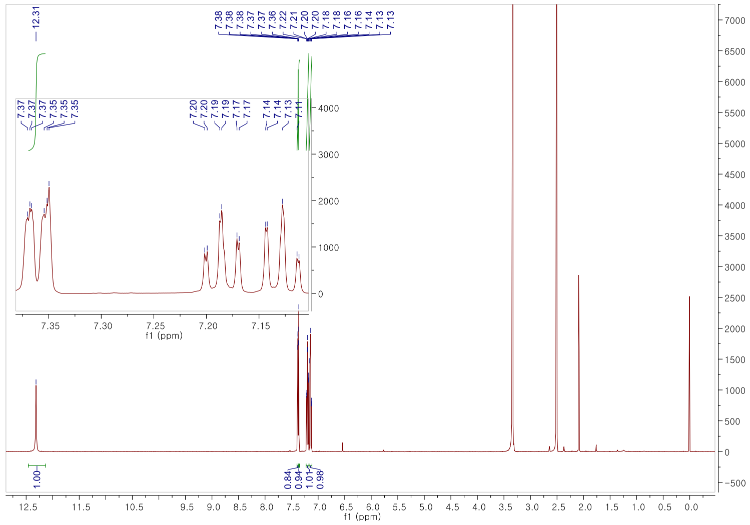
12.31
7.38 7.38 7.38 7.37 7.37 7.36 7.22 7.21 7.20 7.20 7.18 7.18 7.16 7.16 7.14 7.13 7.13
1.00
0.84 0.94 1.01 0.98
f1 (ppm)

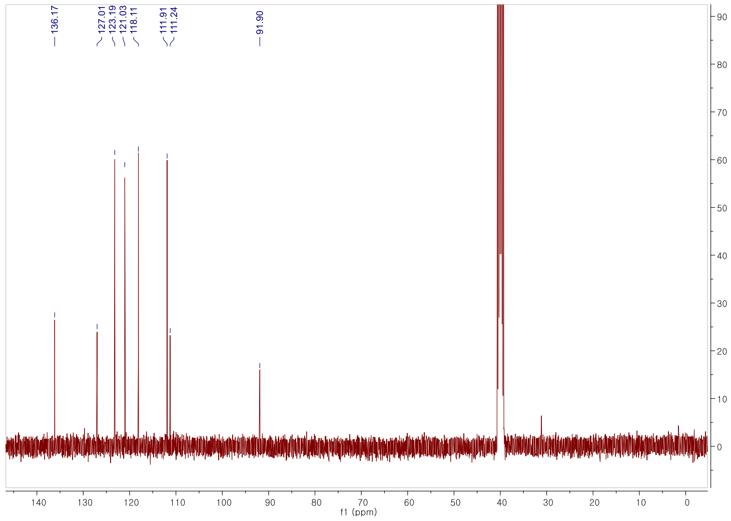
136.17
127.01 123.19 121.03 118.11
111.91 111.24
91.90
f1 (ppm)

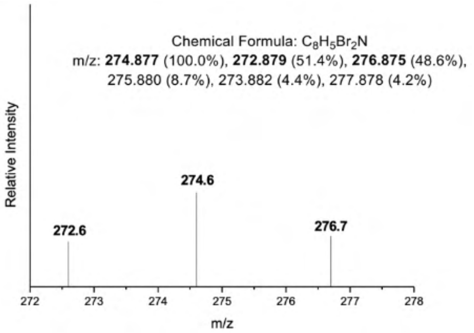
Chemical Formula: $C_8H_5Br_2N$
m/z: **274.877** (100.0%), **272.879** (51.4%), **276.875** (48.6%), 275.880 (8.7%), 273.882 (4.4%), 277.878 (4.2%)
Relative Intensity
272.6
274.6
276.7
m/z

(B13)

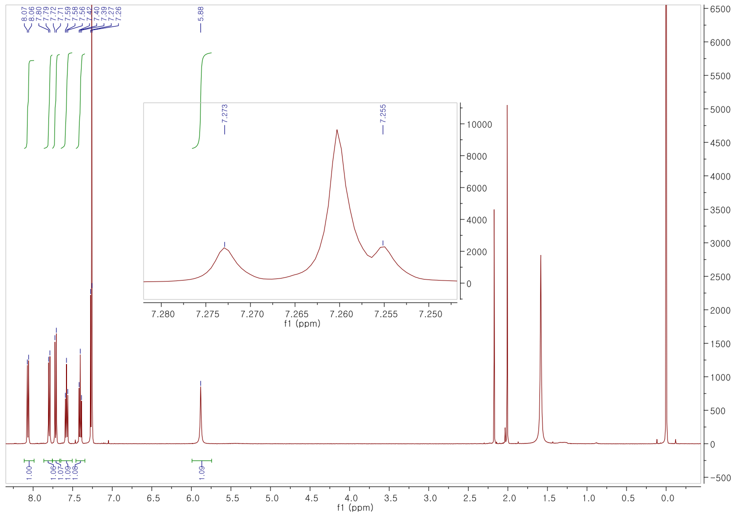

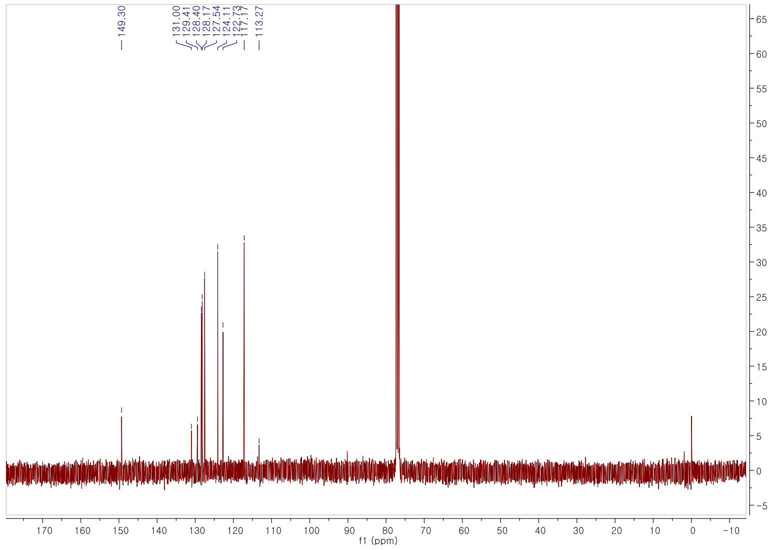

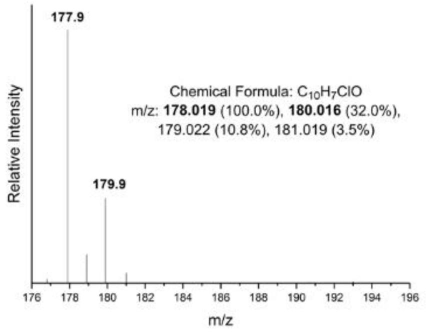
177.9
179.9
Chemical Formula: $C_{10}H_7ClO$
m/z: **178.019** (100.0%), **180.016** (32.0%),
179.022 (10.8%), 181.019 (3.5%)
Relative Intensity
m/z

(B14)

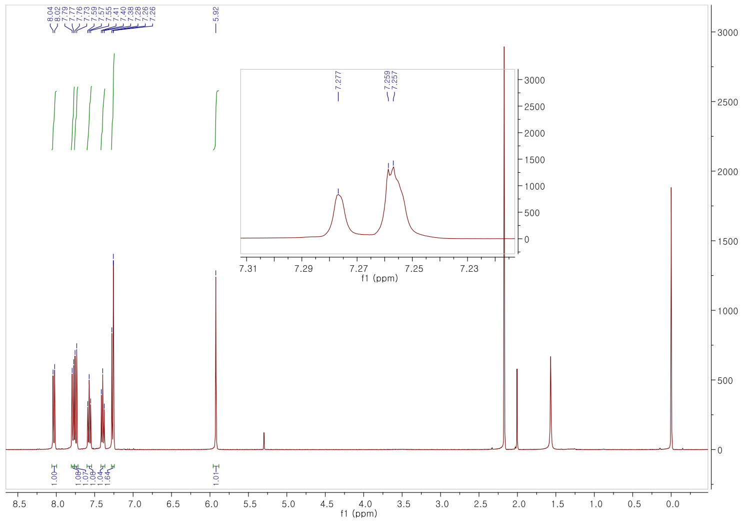
5.92
7.277
7.259
7.257
f1 (ppm)

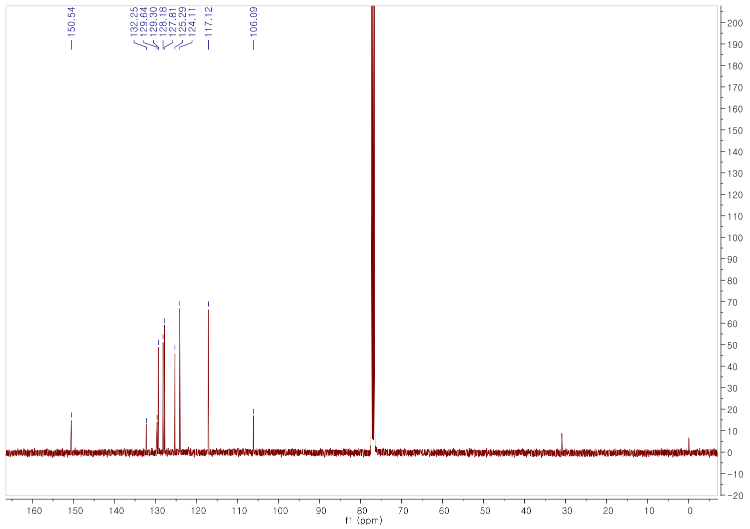
150.54
132.25
129.64
129.30
128.18
127.81
125.29
124.11
117.12
106.09
f1 (ppm)

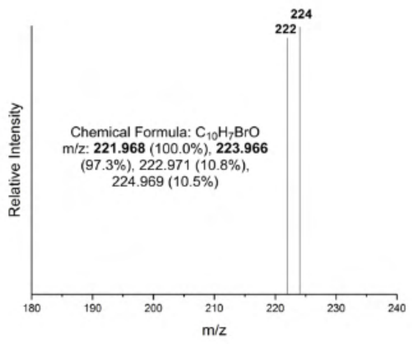
224
222
Chemical Formula: $C_{10}H_7BrO$
m/z: **221.968** (100.0%), **223.966** (97.3%), 222.971 (10.8%), 224.969 (10.5%)
Relative Intensity
m/z

(B15)

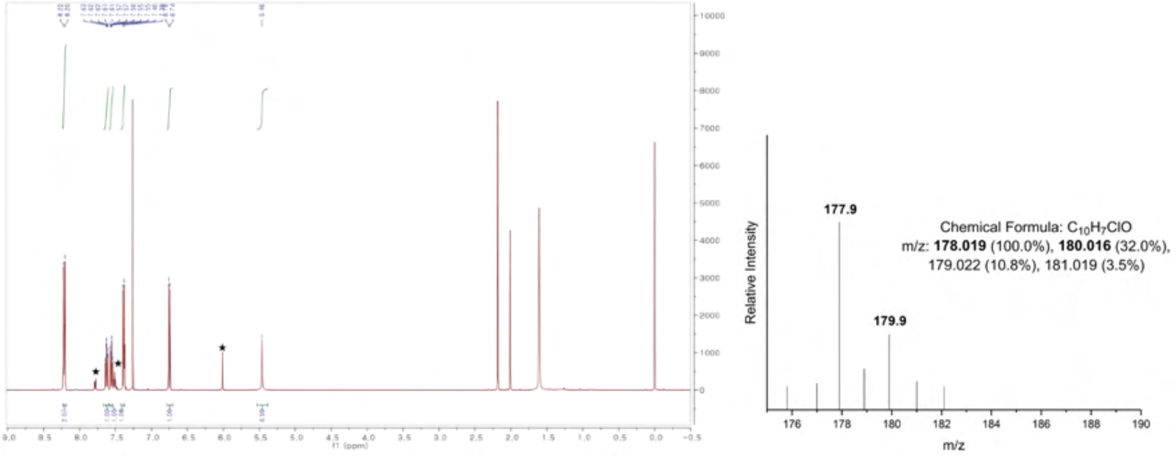

f1 (ppm)
177.9
179.9
Relative Intensity
Chemical Formula: C10H7ClO
m/z: 178.019 (100.0%), 180.016 (32.0%),
179.022 (10.8%), 181.019 (3.5%)
m/z

(B16)

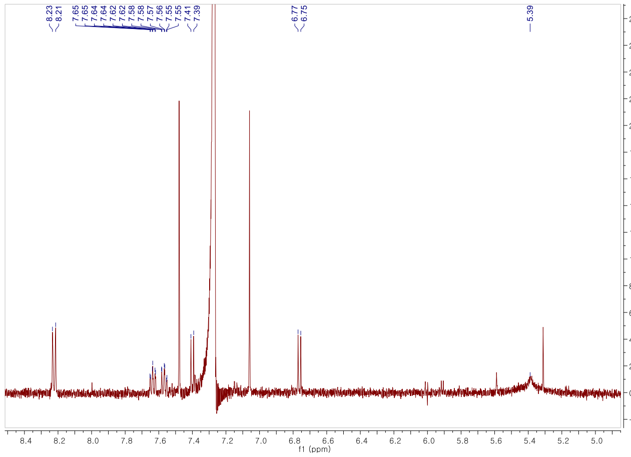

f1 (ppm)

(B17)

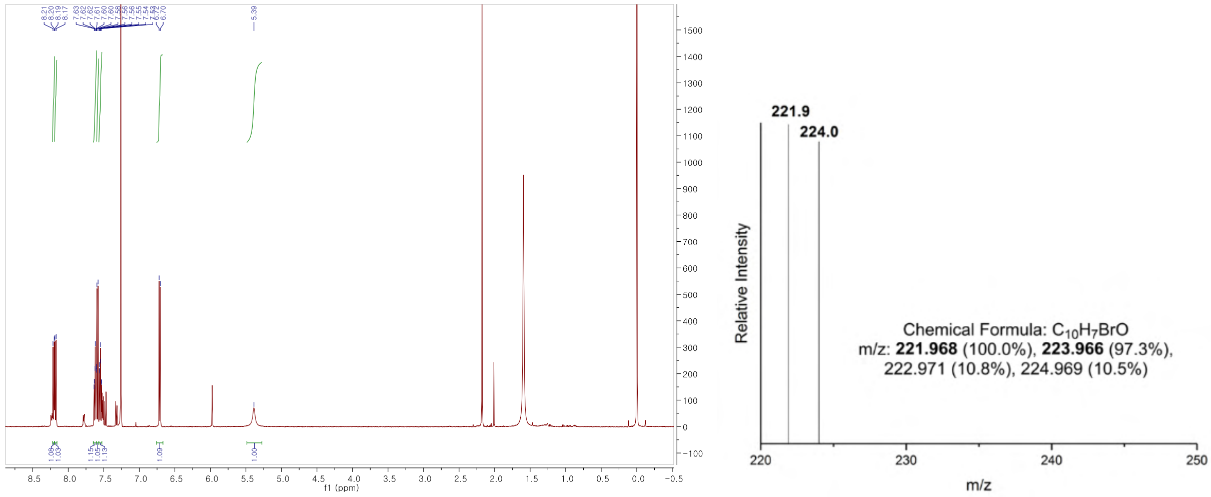

f1 (ppm)
221.9
224.0
Relative Intensity
Chemical Formula: C10H7BrO
m/z: 221.968 (100.0%), 223.966 (97.3%),
222.971 (10.8%), 224.969 (10.5%)
m/z

(B18)

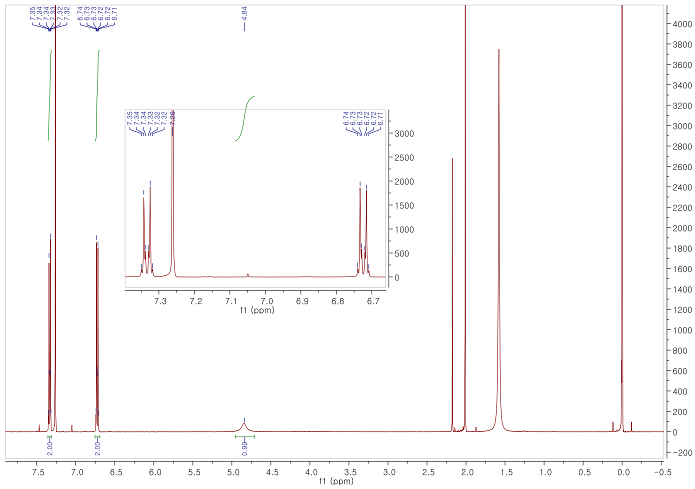

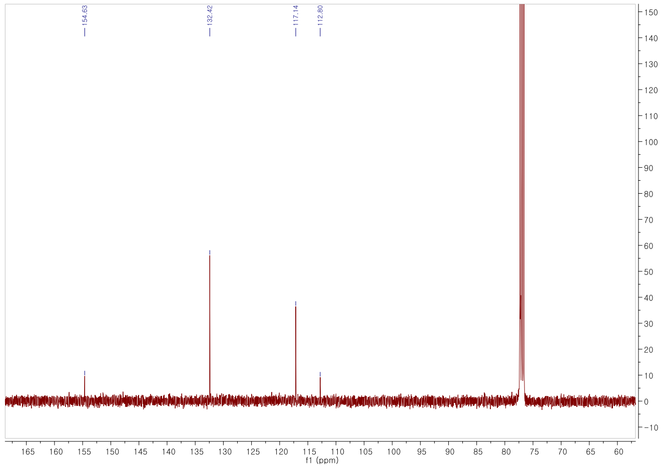

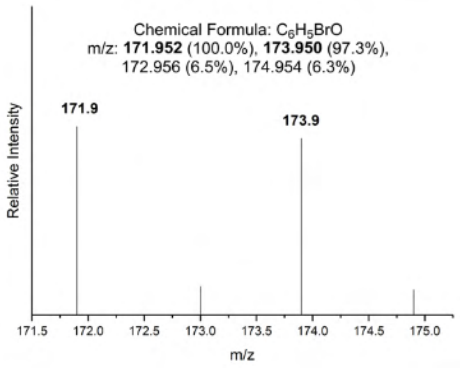
Chemical Formula: C6H5BrO
m/z: 171.952 (100.0%), 173.950 (97.3%),
172.956 (6.5%), 174.954 (6.3%)
171.9
173.9
Relative Intensity
m/z
