# Supplementary material for: Genomic Determinants Encode the Reactivity and Regioselectivity of Flavin-Dependent Halogenases in Bacterial Genomes and Metagenomes
Source: mSystems. 2021 May 27;6(3):e00053-21. doi: 10.1128/mSystems.00053-21 (PMC8269204; doi:10.1128/mSystems.00053-21)
Supplement: TABLE S3 [file msystems.00053-21-st003.docx]

**Table S3**. Conversion and yield in the halogenation of L-tryptophan (Trp) with Hal 1–7, MHal1-4, and the variants. Numbers in parenthesis indicate standard deviation. Asterisks (*) indicate inserted amino acids. The enzyme activities of Hal2 might have been underestimated due to protein instability.

|  | **Conversion (%)** | |  | **Yield (%)** | | | | | |  |
| --- | --- | --- | --- | --- | --- | --- | --- | --- | --- | --- |
|  | **+ NaCl** | **+ NaBr** | **5-Cl-Trp** | **6-Cl-Trp** | **7-Cl-Trp** | **6,7-Di-Cl-Trp** | **5-Br-Trp** | **6-Br-Trp** | **7-Br-Trp** | **6,7-Di-Br-Trp** |
| Genomic FDHs | | | | | | | | | |  |
| Hal1 | 50.2 (4.4) | 51.3 (0.6) |  |  | 58.3 (1.4) |  |  |  | 55.4 (0.4) |  |
| Hal2 | 33.6 (1.4) | 25.8 (2.1) |  | 29.9 (1.1) |  |  |  | 26 (1.7) |  |  |
| Hal3 | 40.1(1.5) | 55.1 (3.2) |  | 39.3 (2.1) |  |  |  | 57.1 (3.2) |  |  |
| Hal4 | 100 (0) | 63.3 (2.3) |  | 98.6 (0.6) |  |  | 16.7 (0.6) | 43.7 (1.7) |  |  |
| Hal5 | 97.5 (1.5) | 100 (0) |  | 95.4 (1.8) |  |  | 15.5 (0.2) | 83.7 (0.5) |  |  |
| Hal6 | 100 (0) | 90.8 (4.2) | 87 (2) |  |  |  | 84.3 (0.7) |  |  |  |
| Hal7 | 100 | 100 |  |  | 86.8 (0.2) | 15.0 (1.3) |  |  | 82.5 (0.1) | 21.1 (0.5) |
| Metagenomic FDHs | | | | | | | | | |  |
| MHal1 | Inactive | Inactive |  |  |  |  |  |  |  |  |
| MHal2 | Inactive | Inactive |  |  |  |  |  |  |  |  |
| MHal3 | Inactive | Inactive |  |  |  |  |  |  |  |  |
| MHal4 | Inactive | Inactive |  |  |  |  |  |  |  |  |
| Hal1 variants (**motifs**) | | | | | | | | | |  |
| Y443A/Y444A (**Trp4**) | 81.1 (1.7) | 90.7 (6.6) |  |  | 82.2 (1.4) |  |  |  | 91.9 (6.4) |  |
| Y443A/Y444A/E450A/N459A (**Trp4**) | Inactive | Inactive |  |  |  |  |  |  |  |  |
| Hal2 variants (**motifs**) | | | | | | | | | |  |
| V52I (**Trp1**) | 50.5 (3.5) | 50.5 (0.5) |  | 48.8 (4) |  |  |  | 50.2 (0.3) |  |  |
| P110L (**Trp2**) | 100 (0) | 100 (0) |  | 88.7 (0.8) |  |  |  | 95.2 (1.1) |  |  |
| S471N (**Trp4**) | 53.5 (0.9) | 52.2 (13.5) |  | 31.9 (0.6) | 19.6 (0.3) |  |  | 38.8 (1.4) | 13.5 (0.5) |  |
| V52I/P110L (**Trp1/Trp2**) | 80.1 (5.4) | 95.5 (2.6) |  | 73.8 (4.2) | 1.2 (0.6) |  |  | 92.7 (2) | 1.4 (0.3) |  |
| V52I/S471N (**Trp1/Trp4**) | 13.3 (1.9) | 12 (3.3) |  | 2.8 (0.1) | 10.5 (0.2) |  |  | 6.5 (0.4) | 6.5 (0.4) |  |
| P110L/S471N (**Trp2/Trp4**) | 22.4 (0.8) | 50.6 (1) |  | 3.8 (0.1) | 15.2 (0.1) |  |  | 16.2 (0.2) | 38 (0.5) |  |
| V52I/P110L/S471N (**Trp1/Trp2/Trp3**) | 39.3 (1.3) | 79.4 (2.5) |  | 1.7 (0) | 36.3 (1.1) |  |  | 11.5 (0.3) | 69.2 (1.8) |  |
| Hal6 variants (**motifs**) | | | | | | | | | |  |
| T51D/V52I (**Trp1**) | 22.6 (1.8) | 16.1 (2) | 16 (0.5) | 3.1 (0) |  |  | 2.6 (0.3) | 11.9 (0.1) |  |  |
| R96Q (**Trp2**) | 91.1 (2.4) | 24.5 (1.9) | 79 (3.1) |  |  |  | 18.2 (0.3) | 1.3 (0) |  |  |
| R161G/*K/A162T (**Trp3**) | 100 (0) | 48.4 (2.2) | 85.4 (0.7) |  |  |  | 39.3 (0.3) |  |  |  |
| Q160A/Q163A (**Trp3**) | 60.1 (4.4) | 9.2 (0.3) | 57.8 (3.9) |  |  |  | 4.2 (0.1) | 2.3 (0.1) |  |  |
| F451L/E452P/S453P (**Trp4**) | Inactive | Inactive |  |  |  |  |  |  |  |  |
| F451L/E452P/S453A (**Trp4**) | Inactive | Inactive |  |  |  |  |  |  |  |  |
| T51D/V52I/R96Q (**Trp1/Trp2**) | 14.3 (1.8) | 9.5 (0) | 8 (0.5) | 2.3 (0) |  |  | 0.9 (0) | 4.2 (0.1) |  |  |
| R161G/*K/A162T/F451L/E452P/S453P  (**Trp3/Trp4**) | 100 (0) | 51.3 (0.8) | 87.5 (1) |  |  |  | 45 (0.3) |  |  |  |
| T51D/V52I/R96Q/F451L/E452P/S453P (**Trp1/Trp2/Trp4**) | Inactive | Inactive |  |  |  |  |  |  |  |  |
| R96Q/R161G/*K/A162T/  F451L/E452P/S453P  (**Trp2/Trp3/Trp4**) | Inactive | Inactive |  |  |  |  |  |  |  |  |
| T51D/V52I/R96Q/ R161G/*K  /A162T/F451L/E452P/S453P  (**Trp1/Trp2/Trp3/Trp4**) | Inactive | Inactive |  |  |  |  |  |  |  |  |
| Hal4 variants (**motifs**) | | | | | | | | | |  |
| D51T/I52V (**Trp1**) | 24.1 (0.1) | 10.0 (0.2) |  | 22.7 (0) |  |  | 3.9 (0.1) | 4.1 (0.1) |  |  |
| Q96R (**Trp2**) | 73.3 (2.4) | 45.2 (3.1) |  | 73.2 (1.4) |  |  | 13.6 (0.6) | 31.0 (1.4) |  |  |
| 456 LPA to FES (**Trp4**) | 100 (0) | 54.2 (0.5) | 22.8 (0.3) | 60.2 (1.4) |  |  | 23.9 (0.4) | 28.5 (0.4) |  |  |
| D51T/I52V/L456F/P457E/A458S  (**Trp1/Trp4**) | 44.3 (0.7) | 50.5 (0.9) | 34.8 (0.4) | 1.4 (0) |  |  | 38.7 (1.2) | 4.1 (0.1) |  |  |
| Q96R/L456F/P457E/A458S (**Trp2/Trp4**) | 76.4 (0.5) | 82.3 (1.5) | 24 (0.6) | 49.6 (1) |  |  | 32.6 (0.3) | 47.7 (0.4) |  |  |
| D51T/I52V/Q96R/L456F/P457E/A458S (**Trp1/Trp2/Trp4**) | 75.6 (2.5) | 78.7 (2.7) | 64 (2.7) | 1.9 (0.2) |  |  | 66.2 (3.3) | 7.5 (0.4) |  |  |
| Hal7 variants (**motifs**) | | | | | | | | | |  |
| I53V (**Trp1**) | 96.2 (1.9) | 99.7 (0.5) |  |  | 92.7 (1.1) | 6.4 (2.7) |  |  | 95.9 (2.4) | 6.7 (0.9) |
| L111P (**Trp2)** | 97.6 (2.1) | 100 (0) |  |  | 96 (0.6) | 4.6 (1.5) |  |  | 86.8 (1) | 18.5 (0.8) |
| N470S (**Trp4**) | 82 (2.2) | 88.3 (5.3) |  | 2.3 (0.1) | 29.4 (0.7) | 59.7 (3.4) |  | 10.5 (0.1) | 41.8 (3.4) | 47.8 (6.6) |
| I53V/ L111P (**Trp1**/**Trp2**) | 99.7 (0.4) | 100 (0) |  |  | 91.4 (1) | 10.2 (0.8) |  | 1.1 (0.6) | 90.3 (0.4) | 11.6 (0.4) |
| I53V/ N470S (**Trp1**/**Trp4**) | 53.3 (1.6) | 78.9 (0.4) |  | 9.4 (0.4) | 8.4 (0.2) | 40.6 (1.7) |  | 32.7 (0.1) | 19.9 (0.2) | 33.1 (0.3) |
| L111P/ N470S (**Trp2**/**Trp4**) | 96.1 (2.2) | 100 (0) |  | 3.1 (0.4) | 81.4 (1.2) | 15.6 (3.4) |  | 8.9 (0.1) | 56.9 (1.6) | 41 (2) |
| I53V/ L111P/ N470S (**Trp1**/**Trp2**/**Trp4**) | 91.1 (3.7) | 99.3 (0.6) |  | 22.5 (0.9) | 49.9 (1.2) | 22.6 (4.5) |  | 48.6 (0.7) | 39.4 (5.5) | 18.3 (5.9) |
