# Supplementary material for: Genomic Determinants Encode the Reactivity and Regioselectivity of Flavin-Dependent Halogenases in Bacterial Genomes and Metagenomes
Source: mSystems. 2021 May 27;6(3):e00053-21. doi: 10.1128/mSystems.00053-21 (PMC8269204; doi:10.1128/mSystems.00053-21)
Supplement: TEXT S1 [file msystems.00053-21-sd001.docx]

**Text S1 Materials and Methods**

**Preparation and biochemical characterization of putative halogenases from genomic and metagenomic sequences**

Cells were grown overnight at 37.5 ^o^C with constant shaking at 170 rpm in 10 mL of terrific broth (TB). For the cell growth with pET-28a(+) vector, 34 μg/mL chloramphenicol and 50 μg/mL kanamycin were used as antibiotics. For protein expression with pET22b(+) vector, 34 μg/mL chloramphenicol and 100 μg/mL ampicillin were added to the growth media. The overnight culture was inoculated to 1 L of TB media including the appropriate antibiotics as described above. Cells were grown at 37.5 ^o^C with 170 rpm shaking until OD_600_ value reached 0.6~0.8. Then, the culture flasks were cooled down to 15 ^o^C and *L*-arabinose and isopropyl *β*-*D*-1-thiogalactopyranoside (IPTG) were added to the final concentration of 2 mg/mL and 0.1 mM, respectively. After constant shaking at 170 rpm for ~19 h at 15 ^o^C, the cells were harvested by centrifugation at 4715 g at 4 ^o^C. The cell pellets were stored at -80 ^o^C until further usage.

The cell pellets of putative FDHs and MR were suspended in 25 mM 4-(2-hydroxyethyl)-1-piperazineethanesulfonic acid (HEPES) pH 7.4 buffer (30 mL buffer per cell pellets from 1L culture) and were lysed using ultrasonic processors (US/VCS750) while kept in ice water. The setting for sonication was 33 % amplitude, pulse on/off = 3.3/3.3 s for 1 h. Soluble fractions were collected by centrifugation at 18800 g at 4 ^o^C for 45 min. After syringe filtration, the supernatants were loaded to 5 mL HisTrap FF affinity column using ÄKTA pure chromatography system (GE Healthcare) at 4 ^o^C. Prior to the protein loading, the column was equilibrated with the lysate buffer, 25 mM HEPES pH 7.4, and the protein was eluted by applying a linear gradient of imidazole concentration increasing from 25 mM to 500 mM. MHal4 was further purified with size exclusion chromatography (HiLoad 16/600 Superdex 200 pg from GE Healthcare) equilibrated with the lysate buffer. Purity of the proteins were identified by 20% SDS-PAGE analysis (Fig. S4A–D). Pure fractions are concentrated using centrifugal filter 30 K or 10 K (Amicon) for FDH or MR, respectively. The purified samples were stored in 25 mM HEPES pH 7.4 buffer at -80 ^o^C.

As a reference for MR, Fre protein was isolated. The cell pellets were suspended, lysed, and filtered as described above. Soluble fractions were loaded to 5 mL HiTrap Q HP column using ÄKTA pure chromatography system (GE Healthcare) at 4 ^o^C. Prior to the protein loading, the column was equilibrated with the lysate buffer, 25 mM HEPES pH 7.4, and the protein was eluted by applying a linear gradient of 0–1 M NaCl concentration. Fractions containing Fre were identified by measuring the consumption rates of NADH. In 96 well plate, 10 μL of 1 mM NADH, 10 μL of 1 mM FAD, and 90 μL of eluted fraction were mixed, and consumption rates of NADH were monitored at 340 nm using a microplate reader (BioTek SYNERGY H1). The fraction containing Fre was further purified with HiLoad 16/600 Superdex 75 pg (GE Healthcare) equilibrated with the lysate buffer at 4 ^o^C. Purity of the proteins were identified by 20% SDS-PAGE analysis (Fig. S4E–K). Pure fractions were concentrated using centrifugal filter 10 K (Amicon). The purified samples were stored in 25 mM HEPES pH 7.4 buffer at -80 ^o^C.

Protein concentration was determined by measuring the absorption at 280 nm using micro-volume spectrophotometer (NanoDrop from Thermofisher). The extinction coefficients of the proteins other than Hal1 and MR were estimated from the amino acid sequences using ProtParam tool on the ExPASy Server. Hal1 and MR were co-isolated with FAD and FMN cofactor, respectively, so that protein concentrations were calculated using the absorbance values at 280 nm and 459 nm. The concentrations of the cofactors were measured with the absorbance at 459 nm using UV-Vis spectrophotometer (Agilent Technologies Cary 8454 UV). The concentration of the proteins was estimated from the absorbance at 280 nm after subtracting those of the cofactors. Extinction coefficients of FAD or FMN at 280 and 459 nm were calculated from standard FAD or FMN solutions.
