# Supplementary material for: Genomic Determinants Encode the Reactivity and Regioselectivity of Flavin-Dependent Halogenases in Bacterial Genomes and Metagenomes
Source: mSystems. 2021 May 27;6(3):e00053-21. doi: 10.1128/mSystems.00053-21 (PMC8269204; doi:10.1128/mSystems.00053-21)
Supplement: TABLE S4 [file msystems.00053-21-st004.docx]

**Table S4.** Substrate scopes of the selected genomic and metagenomic FDHs. Chemical structures of (a) chlorinated and (c) brominated products. Conversion (%) and yield (%) in the chlorination of indole and other aromatic substrates with (b) NaCl and (d) NaBr. Numbers in parenthesis indicate standard deviation. No conversion of (e) benzothiazole, benzimidazole, phenylalanine, and tyrosine was observed for all putative FDHs. Due to poor stability of Hal2, conversion and yield of other than indole were not determined. ND; not determined

(a)


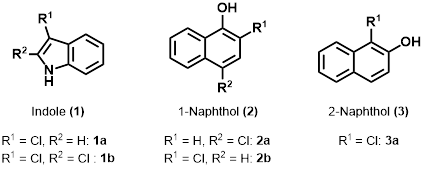


(b)

|  | **Indole (1)** | | **1-Naphthol (2)** | | **2-Naphthol (3)** | | **Phenol (4)** | |
| --- | --- | --- | --- | --- | --- | --- | --- | --- |
|  | Conv | Yield | Conv | Yield | Conv | Yield | Conv | Yield |
| Genomic FDHs | | | | | | | |  |
| Hal1 | 69.5 (1.7) | **1a/**29.8 (0.8), **1b**/33.5 (0.2) | 69.3 (2.4) | **2a**/22.3 (1.5), **2b**/19.1 (0.7) | 56.8 (0.4) | **3a**/50.8 (0.5) | Inactive |  |
| Hal2 | 19.6 (0.6) | **1a**/24.9 (0.3) | ND |  | ND |  | ND |  |
| Hal3 | 47.5 (1.3) | **1a**/47.8 (0.5) | 35.7 | **2a**/23.2 (0.7) | Inactive |  | Inactive |  |
| Hal4 | 46.6 (0.8) | **1a**/44.8 (0.7) | 10.7 (1.6) | **2a**/3.2 (0.5), | Inactive |  | Inactive |  |
| Hal5 | 71.8 (0.8) | **1a**/69.4 (0.8) | 12.1 (0.4) | **2a**/5.5 (0.5) | Inactive |  | Inactive |  |
| Hal6 | 83.4 (0.8) | **1a**/64.8 (1.1), **1b**/18.1 (0.7) | 41.9 (0.4) | **2a**/18.2 (0.1), **2b**/1.3 (0.0) | Inactive |  | Inactive |  |
| Hal7 | 93.1 (0.4) | **1a**/26.4 (1.2), **1b**/60.4 (3.9) | 82.9 (0.9) | **2a**/25.0 (0.8), **2b**/12.5 (0.1) | 37.0 (1.2) | **3a**/36.9 (2.1) | Inactive |  |
| Metagenomic FDHs | | | | | | | |  |
| MHal1 | Inactive |  | Inactive |  | Inactive |  | Inactive |  |
| MHal2 | 7.3 (0.5) | **1a**/5.4 (0) | Inactive |  | Inactive |  | Inactive |  |
| MHal3 | Inactive |  | Inactive |  | Inactive |  | Inactive |  |
| MHal4 | Inactive |  | Inactive |  | Inactive |  | Inactive |  |

(c)


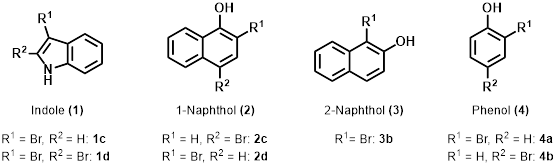


(d)

|  | **Indole (1)** | | **1-Naphthol (2)** | | **2-Naphthol (3)** | | **Phenol (4)** | |
| --- | --- | --- | --- | --- | --- | --- | --- | --- |
|  | Conv | Yield | Conv | Yield | Conv | Yield | Conv | Yield |
| Genomic FDHs | | | | | | | | |
| Hal1 | 70.6 (0.4) | **1c**/54.0 (0.4),  **1d**/9.6 (0.2) | 47.8 (2.3) | **2c**/16.3 (0.5),**2d**/12.0 (0.5) | 84.1 (0.3) | **3b**/80.6 (1.3) | 69.2 (12.3) | **4a**/2.2 (0.3),  **4b**/67.1 (7.1) |
| Hal2 | 27.1 (0.7) | **1c**/28.2 (0.6) | ND |  | ND |  | ND |  |
| Hal3 | 54 (1.9) | **1c**/56.3 (0.5) | 47.9 (0.8) | **2c**/7.1 (0.5), **2d**/22.4 (0.5) | 54.3 (2.1) | **3b**/66.4 (4.5) | 54.4 (14.3) | **4b**/54.4 (2.8) |
| Hal4 | 51 (0.9) | **1c**/49.2 (1) | 20.6 (0.9) | **2c**/3.1 (0.6), **2d**/5.8 (0.6) | 25.2 (1.2) | **3b**/20.9 (0.3) | 22.6 (2.3) | **4b**/14.7 (2.9) |
| Hal5 | 99.3 (0) | **1c**/97.1 (0.4) | 95.5 (1.2) | **2c**/5.4 (0.1), **2d**/55.5 (0.1) | 93.7 (0.5) | **3b**/95.5 (1.2) | 79.9 (6.3) | **4b**/80 (8.3) |
| Hal6 | 100 (0) | **1c**/76.9 (1.4) | 30.3 (1.2) | **2c**/6.8 (0.5), **2d**/8.1 (0.5) | 73.3 (0.6) | **3b**/53.0 (1.3) | 69.8 (5.7) | **4b**/70.1 (8.3) |
| Hal7 | 98.5 (0.3) | **1c**/44.3 (0.7)  **1d**/55.5 (0.6) | 72.3 (0.3) | **2c**/15.7 (0.2),**2d**/13.4 (0.8) | 100 (0) | **3b**/85.7 (1.6) | 100 (0) | **4b**/97.4 (0.9) |
| Metagenomic FDHs | | | | | | | | |
| MHal1 | Inactive |  | Inactive |  | Inactive |  | Inactive |  |
| MHal2 | 65.5 (1) | **1c**/60.7 (1.8) | 31.4 (1.5) | **2d**/ 22.5(0.4) | 36.5 (1.5) | **3b**/25.6 (0.5) | Inactive |  |
| MHal3 | 23.2 (1.6) | **1c**/10.6 (0.6) | 3.9 (1.5) | **2d** /6.9 (0.2) | 24.5 (0.3) | **3b**/12.9 (0.2) | 30.4 (3.5) | **4a**/22.2 (0.6) |
| MHal4 | 47.1 (0.6) | **1c**/36.3 (0.4) | 19.0 (1.5) | **2d** /13.6 (0.3) | 68.7 (2.4) | **3b**/67.1 (1.8) | 6.7 (0.1) | **4a**/7.1 (0.3) |

(e)
